# Yi-Shen-Hua-Shi Granule Alleviates Adriamycin-Induced Glomerular Fibrosis by Suppressing the BMP2/Smad Signaling Pathway

**DOI:** 10.3389/fphar.2022.917428

**Published:** 2022-06-15

**Authors:** Zhuojing Tan, Yachen Si, Yan Yu, Jiarong Ding, Linxi Huang, Ying Xu, Hongxia Zhang, Yihan Lu, Chao Wang, Bing Yu, Li Yuan

**Affiliations:** ^1^ Department of Nephrology, Affiliated Hospital of Nantong University, Nantong, China; ^2^ Department of Cell Biology, Naval Medical University (Second Military Medical University), Shanghai, China; ^3^ Department of Internal Medicine, No. 944 Hospital of Joint Logistics Support Force, Jiuquan, China; ^4^ Department of Nephrology, Changhai Hospital, Naval Medical University (Second Military Medical University), Shanghai, China; ^5^ Nanjing Medical University, Nanjing, China

**Keywords:** renal fibrosis (RF), focal segmental glomerulosclerosis (FSGS), Yi-Shen-Hua-Shi granule, BMP2, adriamycin

## Abstract

Focal segmental glomerulosclerosis (FSGS) is a common clinical condition with manifestations of nephrotic syndrome and fibrosis of the glomeruli and interstitium. Yi-Shen-Hua-Shi (YSHS) granule has been shown to have a good effect in alleviating nephrotic syndrome (NS) in clinical and in animal models of FSGS, but whether it can alleviate renal fibrosis in FSGS and its mechanism and targets are not clear. In this study, we explored the anti-fibrotic effect and the targets of the YSHS granule in an adriamycin (ADR)-induced FSGS model and found that the YSHS granule significantly improved the renal function of ADR-induced FSGS model mice and also significantly reduced the deposition of collagen fibers and the expression of mesenchymal cell markers FN, vimentin, and α-SMA in the glomeruli of ADR-induced FSGS mice, suggesting that the YSHS granule inhibited the fibrosis of sclerotic glomeruli. Subsequently, a network pharmacology-based approach was used to identify the potential targets of the YSHS granule for the alleviation of glomerulosclerosis in FSGS, and the results showed that the YSHS granule down-regulated the expressions of BMP2, GSTA1, GATS3, BST1, and S100A9 and up-regulated the expressions of TTR and GATM in ADR-induced FSGS model mice. We also proved that the YSHS granule inhibited the fibrosis in the glomeruli of ADR-induced FSGS model mice through the suppression of the BMP2/Smad signaling pathway.

## Introduction

FSGS accounts for 20% of NS in children and 40% in adults, with an increasing incidence rate (about seven patients per million). FSGS is one of the common causes of steroid-resistant nephrotic syndrome (SRNS) and end-stage renal disease (ESRD) in adults and children worldwide (Kopp, 2018; Hodson et al., 2022). The main clinical manifestations of patients with FSGS are NS (massive proteinuria, hypoproteinemia, edema, and hyperlipidemia), hematuria, hypertension, and the impairment of renal function, etc. The pathological changes of FSGS include glomerulosclerosis, tubular atrophy, and interstitial fibrosis in the affected segments of renal tissues (D'Agati et al., 2011; Agrawal et al., 2018).

Renal fibrosis, which is the pathological repair of the damaged renal tissue caused by various chronic renal injuries, plays an important role in the progression of FSGS to ESRD. The proliferation of glomerular mesangial cells and renal tubulointerstitial fibroblasts, the accumulation of the extracellular matrix (ECM), and epithelial–mesenchymal transition (EMT) are involved in the progression of renal fibrosis (Liu, 2011; Dongre and Weinberg, 2019). The severity of renal fibrosis is closely related to the progress of renal insufficiency; thus, it is extremely important to find a targeted drug or treatment strategy that can effectively prevent the progression of renal fibrosis in FSGS. The mechanism of renal fibrosis is sophisticated, and many kinds of signal pathways, such as the transforming growth factor-β (TGF-β)/Smad signaling pathway (Yang et al., 2019), Wnt/β-catenin pathway (Miao et al., 2019), and Notch pathway (Yu et al., 2021), are involved in the progression of renal fibrosis. Therefore, the treatment of renal fibrosis may require a synergistic strategy to act on multiple targets and multiple signaling pathways to achieve better efficacy outcomes. Traditional Chinese medicine (TCM), which has the characteristics of multi-components and multi-targets, has achieved good therapeutic effect in the treatment of renal fibrosis-related diseases (Shen et al., 2018). The ErHuang Formula attenuates renal fibrosis in streptozotocin-induced diabetic nephropathy rats by inhibiting the CXCL6/JAK/STAT3 signaling pathway (Shen et al., 2019). Chinese herbal compound Tongxinluo inhibits renal fibrosis in diabetic nephropathy by preventing the transfer of TGF-β1 from glomerular endothelial cells to glomerular mesangial cells via exosomes (Wu et al., 2017).

The YSHS granule is a modern patent TCM drug approved by China National Medical Products Administration. It was derived from a TCM formula, Sheng-Yang-Yi-Wei decoction, which was first documented in the TCM classic Nei-Wai-Shang-Bian-Huo-Lun issued in 1247 AD. The formula is able to tonify the yang and spleen (‘Sheng Yang Bu Pi’ in Chinese), replenish the kidney, and resolve dampness (‘Yi Shen Hua Shi’ in Chinese), and increase urine excretion to reduce edema (‘Li Shui Xiao Zhong’ in Chinese) (Li, 2018). YSHS granule is composed of 16 herbs, including Ginseng Radix et Rhizoma (GRR), Astragali Radix (ASR), Atractylodis Macrocephalae Rhizoma (AMR), Poria (POR), Alismatis Rhizoma (ALR), Pinelliae Rhizoma Praeparatum Cum Alumine (PRP), Notopterygii Rhizoma et Radix (NRR), Angelicae Pubescentis Radix (APR), Saposhnikoviae Radix (SAR), Bupleuri Radix (BUR), Coptidis Rhizoma (COR), Paeoniae Radix Alba (PRA), Citri Reticulatae Pericarpium (CRP), Glycyrrhizae Radix et Rhizoma Praeparata Cum Melle (GRP), Zingiberis Rhizoma Recens (ZRR), and Jujubae Fructus (JUF). The YSHS granule has achieved good therapeutic effect on treating chronic glomerulonephritis (CGN) in clinic and C-BSA-induced CGN rat models (Zhao et al., 2019). However, the specific mechanism and the potential targets of YSHS against nephropathy remain unclear.

Network pharmacology offers an effective approach for investigating the multiple pharmacological effects of TCM from a molecular perspective (Hopkins, 2007). It can clarify the complex interactions between genes and proteins related to drugs or diseases by integrating cheminformatics, bioinformatics, system biology, and other related research fields. The systematic and holistic characteristics of network pharmacology are consistent with the complex mechanism of “multi-component, multi-target, and multi-pathway” within TCM (Hopkins, 2008). The identification of active components of TCM, component-targeting proteins, and disease-specific molecules is used to establish the component–target-disease network. The component–target interaction network contributes to clarify the molecular mechanism of TCM. The disease–target interaction network can be utilized to explore the pathogenesis (Niu et al., 2018). At present, an integration of network pharmacology and experimental validation has become an important measure to understand the targets of active components and the potential mechanism of TCM.

The ADR-induced renal injury model is an ideal FSGS model, which is often accompanied by glomerular and tubular fibroses (Wang et al., 2000). In this study, the potential active components and targets of the YSHS granule against glomerular injury in FSGS were predicted with network pharmacology. Moreover, the key targets were further verified by experiments. On the basis of the results, the characteristics and functions of the key targets were analyzed and discussed, which laid the foundation of the molecular mechanism of the YSHS granule against ADR-induced FSGS.

## Materials and Methods

### Reagents

Doxorubicin hydrochloride (D807083, Macklin, China) was dissolved in 0.9% NaCl to make a 1 mg/ml solution. The YSHS granule was provided by Guangzhou Consun Pharmaceutical Co., Ltd., and was dissolved in deionized water to make a 200 mg/ml solution. Triglyceride assay kit (A110-1-1), total cholesterol assay kit (A111-1-1), creatinine assay kit (sarcosine oxidase) (C011-2-1), albumin assay kit (A028-2-1), and microalbumin assay kit (H127-1-2) were purchased from Nanjing Jiancheng Bioengineering Institute.

### Real-Time Polymerase Chain Reaction

Total RNA was purified with the RNAiso Plus reagent (9,108, Takara). Then, cDNA was synthesized by the MMLV Reverse Transcriptase (M530A, Promega) according to the manufacturer’s protocols. A real-time PCR analysis was performed on the LightCycler^®^ 96 System (Roche) in the 20 μL reaction mixture containing 10 μL 2 × ChamQ SYBR qPCR Master Mix (Q321-02, Vazyme), 0.2 mM forward primer, 0.2 mM reverse primer, and 0.5 μL cDNA. The PCR amplification conditions were as follows: annealing at 95°C for 10 s, amplification at 60°C for 30 s, and the number of cycles was set to 40. The primers were summarized in Table 1. All samples were examined thrice. The fold changes of each target gene were calculated using the 2^-△△Ct^ method relative to GAPDH.

**TABLE 1:** Primers used in the real-time PCR analysis.

Gene symbol	Forward primer (5′→3′)	Reverse primer (5′→3′)
Gapdh	TGC​GAC​TTC​AAC​AGC​AAC​TCC	TGC​TGT​AGC​CGT​ATT​CAT​TGT​C
Bmp2	TCT​TCC​GGG​AAC​AGA​TAC​AGG	TGG​TGT​CCA​ATA​GTC​TGG​TCA
Gsta1	GCA​AGG​AAG​GCT​TTC​AAG​ATT​CA	TTG​CAA​AAT​AGC​CAG​GAT​CAA​CA
Gsta3	GGG​CTG​ATA​TTG​CCC​TGG​TT	TGG​CTG​CCA​GGT​TGA​AGA​AA
Bst1	TGC​TCG​TTA​TGA​GCT​ATG​GGG	TCA​AGT​CCA​GAG​GCA​TTT​TCC
S100a9	ATA​CTC​TAG​GAA​GGA​AGG​ACA​CC	TCC​ATG​ATG​TCA​TTT​ATG​AGG​GC
Ttr	TTG​CCT​CGC​TGG​ACT​GGT​A	TTA​CAG​CCA​CGT​CTA​CAG​CAG
Gatm	TTG​AGT​ACC​GAG​CGT​ACA​GGT	TCC​ACG​GAA​TGG​ATG​GGA​TAA​T
Igf1	CAC​ATC​ATG​TCG​TCT​TCA​CAC​C	GGA​AGC​AAC​ACT​CAT​CCA​CAA​TG
Ren	ACG​GGT​CCG​ACT​TCA​CCA​T	TGC​CTA​GAA​CAC​CGT​CAA​ACT
Pck1	AGC​ATT​CAA​CGC​CAG​GTT​C	CGA​GTC​TGT​CAG​TTC​AAT​ACC​AA
Alb	TGC​TTT​TTC​CAG​GGG​TGT​GTT	TTA​CTT​CCT​GCA​CTA​ATT​TGG​CA
Pah	GAG​CCT​GAG​GAA​CGA​CAT​TGG	CTG​ATT​GGC​GAA​TCT​GTC​CAG
Ctsv	ATCGCCACCAGAAGCACA	AAACGCCCAACAAGACCC
Dcxr	ACT​GTG​CTG​GCG​TTG​AAG​G	CGG​GTC​CCA​CAT​TGC​TTA​GG
Tnnc1	GCG​GTA​GAA​CAG​TTG​ACA​GAG	CCA​GCT​CCT​TGG​TGC​TGA​T

### Western Blot

Western blots were performed as described previously (Li et al., 2022). In brief, the total protein was prepared with RIPA buffer (89,901, ThermoFisher), quantified by BCA kit (23,225, ThermoFisher), separated by SDS-PAGE electrophoresis, and was transferred to PVDF membranes. Then, the membranes were blocked with 5% skimmed milk powder for 30 min, and incubated with a primary antibody at 4°C overnight. The membranes were washed with TBST for three times, and incubated with an HRP-conjugated secondary antibody at 37°C for 1 h. The immune complexes were visualized by using Pierce™ ECL Western Blotting Substrate (32,132, ThermoFisher). The primary antibodies are listed as follows: E-cadherin (#3195, diluted 1:2000), N-cadherin (#4061, diluted 1:2000), Vimentin (#5741, diluted 1:2000), and α-SMA (#19245, diluted 1:2000) from Cell Signaling Technology, MMP9(PAA553Mu01, diluted 1:2000) from Cloud-Clone Corp, BMP2(66,383-1-lg, diluted 1:2000), beta actin (66,009-1-lg, diluted 1:5,000), and Alpha Tubulin (66,031-1-lg, diluted 1:5,000) from Proteintech.

### Histopathological Analyses

Hematoxylin and Eosin (H&E), immunohistochemistry (IHC), and immunoflurosence (IF) staining were performed as our previous report (Yu et al., 2020). For immunostaining, the rehydrated sections were immersed in Tris-EDTA buffer (pH 9.0) and heated at 121°C for 2 min to repair the antigen. The endogenous peroxidase activity was blocked with 3% H_2_O_2_ for 10 min at room temperature. The non-specific binding site was blocked with 1% BSA for 20 min at room temperature. The sections were incubated with primary antibodies at 4 °C overnight. For IHC staining, the sections were incubated with HRP-conjugated secondary antibodies at 37°C for 30 min, and visualized with DAB Substrate (34,002, ThermoFisher). For IF staining, the sections were incubated with fluorescein-conjugated secondary antibodies at 37°C for 30 min, then the nuclei were counter stained with DAPI. The primary antibodies are listed as follows: rabbit anti-NPHS2 (20384-1-AP, diluted 1:200), mouse anti-BMP2 (66,383-1-lg, diluted 1:200) and rabbit anti-Fibronectin (15613-1-AP, diluted 1:200) from ProteinTech Group, rabbit anti-Vimentin (#5741, diluted 1:200) and rabbit anti-α-SMA (#19245, diluted 1:200) from Cell Signaling Technology. The secondary antibodies are listed as follows: Peroxidase AffiniPure goat anti-rabbit IgG (H+L) (111-035-003, diluted 1:400), Peroxidase AffiniPure goat anti-mouse IgG (H+L) (115-035-003, diluted 1:400), and Cy™3 AffiniPure goat anti-rabbit IgG (H+L) (111-165-003, diluted 1:400) from Jackson ImmunoResearch.

For Sirius Red staining, the hydrated sections were stained with a Sirius Red staining kit (SJ1207, Shuangjian Biotech, China) according the manufacturer’s instruction. For Masson staining, the hydrated sections were stained with Masson’s Trichrome Stain Kit (G1340) from Solarbio (Beijing China) according the manufacturer’s instruction. For PAS staining, the hydrated sections were stained by the Glycogen Periodic Acid Schiff (PAS/Hematoxylin) Stain Kit (G1281) from Solarbio (Beijing China) according to the manufacturer-provided user manual.

The extent of glomerular sclerosis was assessed by examining all glomeruli on a kidney cross-section and calculating the percentage involved (Angeletti et al., 2020).

### Identification of Active Components and Targets of the Yi-Shen-Hua-Shi Granule

The chemical components of the YSHS granule were obtained from the reported literature (Chan et al., 2021). The Traditional Chinese Medicine Systems Pharmacology database (TCMSP, https://tcmspw.com/tcmsp.php) was used to assess the pharmacokinetics of these components (Ru et al., 2014). Active components with an oral bioavailability (OB) ≥ 30% and drug similarity (DL) ≥ 0.18 were selected for subsequent analysis (Xu et al., 2012). The target proteins of the active components in the YSHS granule were achieved from PharmMapper (http://www.lilab-ecust.cn/pharmmapper/) (Liu et al., 2010).

### Screening for Differentially Expressed Genes of Focal Segmental Glomerulosclerosis

Expression profiling data from GSE129973 were obtained from the GEO database (http://www.ncbi.nlm.nih.gov/geo/). The data platform was GPL17586 HTA-2_0 Affymetrix Human Transcriptome 2.0 Array, consisting of 20 normal and 20 FSGS samples. Transcriptome comparison of glomeruli from kidneys with FSGS and glomeruli from the unaffected portion of tumor nephrectomies was progressed. The GEO2R web tool (http://www.ncbi.nlm.nih.gov/geo/geo2r/) using the limma package (version 3.26.8) based on the R language (version 3.2.3) was utilized to screen differentially expressed genes (DEGs) with the criteria of adjusted *p*-value < 0.05 and |Log2FC| > 1.

### Construction of the Component–Target–Disease Network

The targets of the YSHS granule against FSGS were achieved by taking the intersection of the target proteins of active components in the YSHS granule and the DEGs of FSGS. A component–target–disease network was constructed based on the targets of the YSHS granule against FSGS with the Cytoscape (http://cytoscape.org) software (Shannon et al., 2003).

### Gene Ontology and Kyoto Encyclopedia of Genes and Genomes Pathway Enrichment Analysis

ClusterProfiler software package in R platform was adopted to conduct gene ontology (GO) and kyoto encyclopedia of genes and genomes (KEGG) pathway enrichment analyses based on the targets of the YSHS granule against FSGS (Yu et al., 2012). The process and pathway with *p* < 0.05 were considered significant.

### Animal Experiments

Male BALB/c mice were provided by Shanghai SLAC Lab Animal Co., Ltd., and were kept in standard pathogen-free conditions. All animal experiments were met with the Guidance for the Care and Use of Laboratory Animals and were approved by the Institutional Ethics Committee of Naval Medical University. The mice were randomly divided into three groups: The first group was the control group treated with saline by gavage administration. The second group was treated with doxorubicin hydrochloride (10 mg/kg body weight) by single intravenous injection to establish the mouse FSGS model, and then was randomly divided into two groups: the ADR group and the ADR + YSHS group, which were separately treated with saline and indicated dosage of the YSHS granule by gavage administration after ADR injection for 12 h. The third group was the YSHS group only treated with the indicated dosage of the YSHS granule by gavage administration. Each group was given saline or YSHS granule by gavage administration once a day for 28 days. Urine was collected on the last day. On the 29th day, all the mice were anesthetized by intraperitoneal injection of 1.25% tribromoethanol, the whole blood was collected by eyeball extraction and set up at room temperature for 2 h, then, the serum was collected by the centrifugation at 4,000 rpm for 10 min. The levels of ALB, Cr, TG, and TC in the serum were measured according the manufacturer’s instruction. The right kidney was used for RNA and protein extraction. The left kidney was fixed with 4% paraformaldehyde, and then was paraffin-emdedded and sectioned at 3–7 μm.

### Urine Albumin and Creatinine Quantification

12 h-urine samples were collected in metabolic cages from individual mice on the last day of molding. Urinary creatinine and urinary albumin were determined using commercial assay kits from Nanjing Jiancheng Bioengineering Institute. Urinary albumin excretion was expressed as the ratio of urinary albumin to creatinine.

### Statistical Analysis

All experimental data were shown as mean ± SD and analyzed by one-way analysis of variance (ANOVA) using Prism, version 8, for Windows (GraphPad Software Inc.). *P* < 0.05 was considered statistically significant.

## Results

### Yi-Shen-Hua-Shi Granule Alleviates Renal Injury in the Adriamycin-Induced Focal Segmental Glomerulosclerosis Model

**FIGURE 1:**
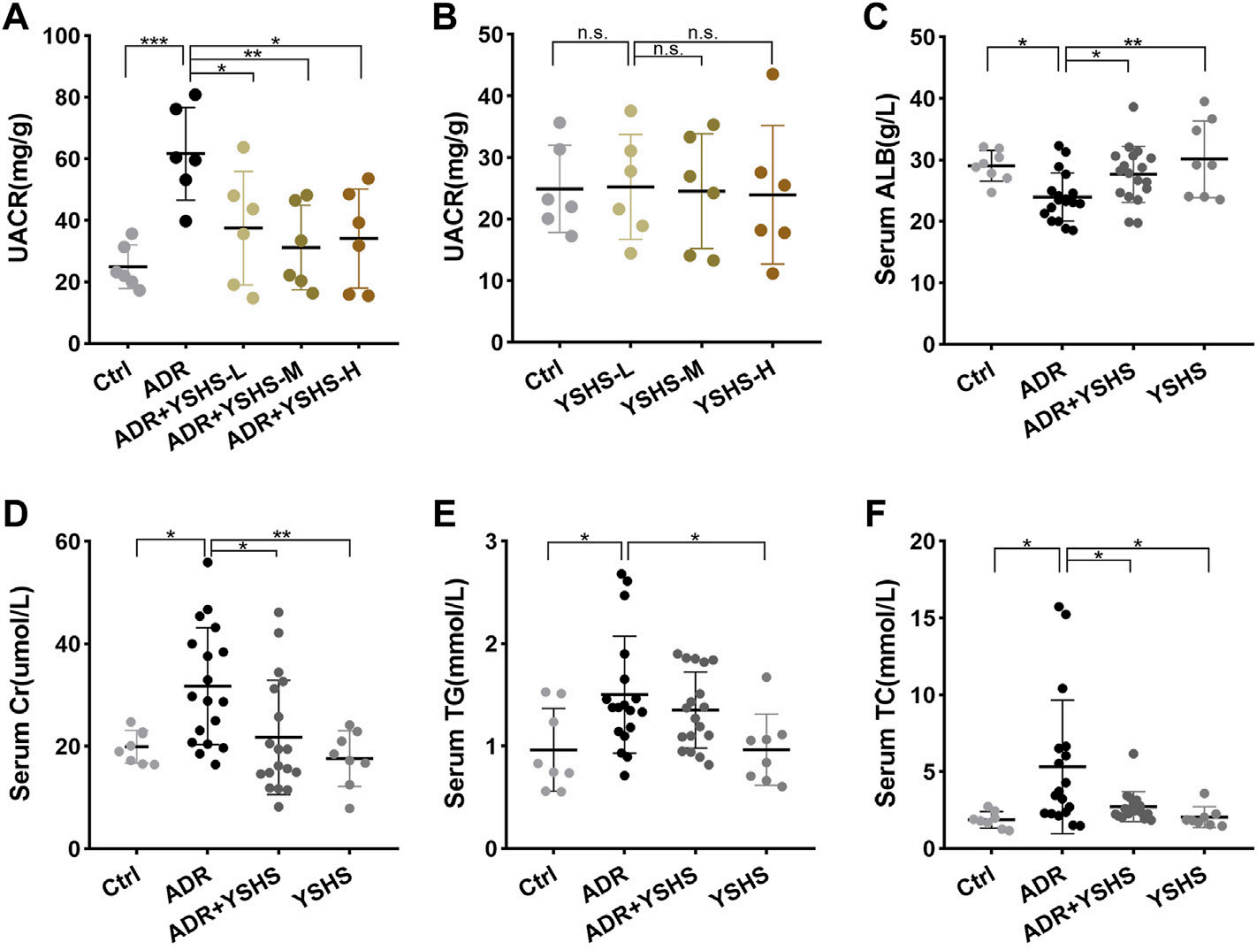
YSHS granule mitigates kidney injury and proteinuria in ADR-induced nephropathy. **(A)** At the end of the fourth week of intragastric administration, 12 h-urine samples were collected. The UACR (mg/g) was evaluated from mice in Ctrl, ADR, and ADR with three different concentrations of YSHS treatment groups. **(B)** Three different doses of YSHS did not cause significant changes in UACR in healthy mice. **(C–F)** Serum albumin (ALB) **(C)**, creatinine (Cr) **(D)**, triglyceride (TG) **(E)** , and total cholesterol (TC) **(F)** were evaluated in the mice of the indicated groups. The data represent mean ± SD, one-way ANOVA analysis followed by Tukey post-hoc tests as appropriate for multiple comparisons. *p < 0.05, **p < 0.01, ***p < 0.001, n. s., no significant.

To determine the optimal therapeutic dose of the YSHS granule for the alleviation of renal injury in the ADR-induced FSGS mouse model, the doses of 2,000, 4,000 and 8,000 mg/kg body weight of the YSHS granule were administered by gavage once daily, and the urinary albumin/creatinine ratio (UACR), which is one of the most sensitive and reliable response indicators of early renal injury (Basset et al., 2022), was measured after 4 weeks of intragastric administration of the YSHS granule. The results showed that the UACR decreased after intragastric administration of three doses of the YSHS granule, but the therapeutic effects of 4,000 and 8,000 mg/kg body weight were better than that of 2,000 mg/kg body weight. Moreover, there was no significant difference between the therapeutic effects of 4,000 and 8,000 mg/kg body weight (Figure 1A). Therefore, the dosage of 4,000 mg/kg body weight was used as the optimal treatment dose for the following study. In addition, the intragastric administration of the YSHS granule at doses of 2,000, 4,000, and 8,000 mg/kg body weight to healthy mice alone did not cause obviously change in the UACR (Figure 1B), indicating that YSHS had no significant toxicity to the kidney. To further clarify the therapeutic effect of the YSHS granule, we performed biochemical assays on mouse serum specimens, and the results showed that the YSHS granule elevated serum albumin (Figure 1C) and reduced serum creatinine (Figure 1D), triglyceride (Figure 1E), and total cholesterol (Figure 1F), which met the criteria for an effective treatment of FSGS.

**FIGURE 2:**
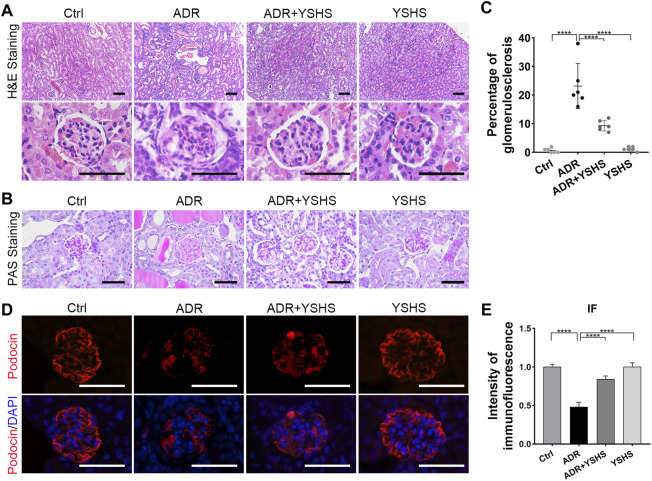
YSHS granule ameliorates glomerular injury in the FSGS model. **(A)** Representative H&E staining of the renal sections in the indicated groups. **(B)** Representative PAS staining of the renal sections in the indicated groups. **(C)** The percentage of glomerulosclerosis in each group. **(D)** Representative IF staining of podocin (red) in the indicated groups. DAPI was used to visualize the cell nucleus (blue). **(E)** The quantification of the intensity of podocin revealed that the YSHS granule significantly increased the expression of podocin in the glomeruli of the FSGS model. All glomeruli from one section of 6 mice in each group were analyzed and the data were shown as mean ± SD, one-way ANOVA analysis followed by Tukey post-hoc tests as appropriate for multiple comparisons, ****p < 0.0001. scale bar: 50 µm.

To clarify the effect of the YSHS granule on the morphological structure of glomeruli in the ADR-induced FSGS mouse model, a series of pathological tests were performed. The results of HE and PAS staining showed that compared with the control group, ADR caused obvious glomerular segmental sclerosis, the formation of undifferentiated cells on the surface of the basement membrane adhering to the wall of Bowman’s capsule, the dilatation and structural disorder of proximal tubules, a large number of protein casts in the tubular lumen, and extensive interstitial infiltration of inflammatory cells (Figures 2A,B). In contrast, the incidence and extent of glomerulosclerosis were significantly reduced in FSGS model mice after treatment with the YSHS granule (Figure 2C). Podocytes are an important component of the glomerular filtration membrane, and the damage to the filtration membrane is a key factor in causing proteinuria (Angeletti et al., 2020). The results of immunofluorescence staining showed that the number of podocytes in the glomeruli of the FSGS mouse model induced by ADR was significantly reduced, indicating that ADR had a significant damaging effect on podocytes, while the treatment with the YSHS granule significantly increased the number of podocytes in the glomeruli of the FSGS model (Figures 2D,E), suggesting that YSHS granule could reduce the damage of ADR on podocytes. These data suggest that YSHS granule has a promising therapeutic effect on ADR-induced focal segmental glomerulosclerosis.

### Yi-Shen-Hua-Shi Alleviates Glomerular Fibrosis in the Focal Segmental Glomerulosclerosis Model

**FIGURE 3:**
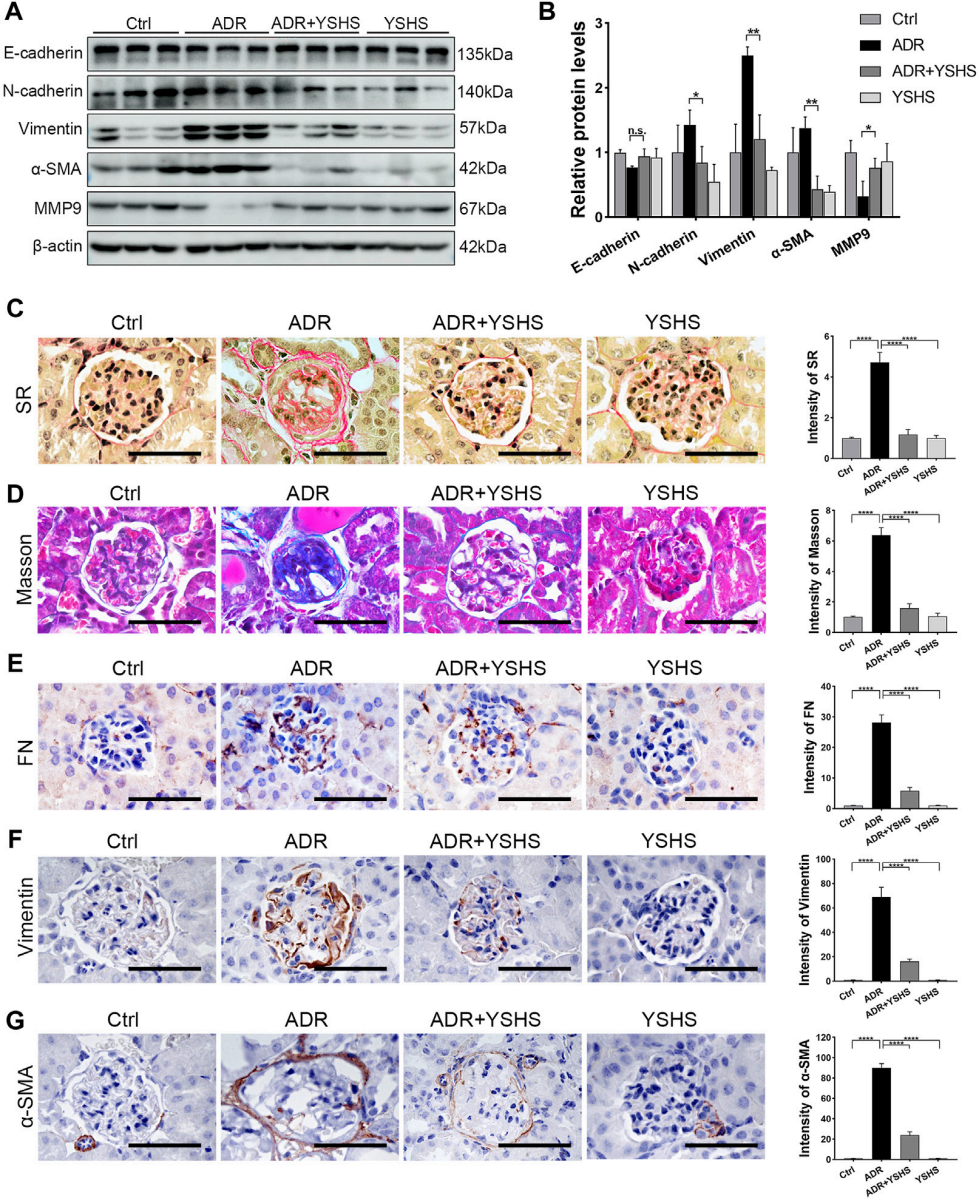
YSHS granule relieves glomerular fibrosis in the FSGS model. **(A)** Western blot analysis shows the expression levels of the indicated proteins extracted from kidney tissues in each group. **(B)** The quantification of the relative intensities of blots showed that the YSHS granule reduced the expression of N-cadherin, Vimentin, and α-SMA in ADR-induced FSGS models. *N* = 3 in each group, the data were shown as mean ± SD, one-way ANOVA analysis followed by Tukey post-hoc tests as appropriate for multiple comparisons, *p < 0.05, **p < 0.01. **(C–D)** The results of Sirius Red (SR) staining **(C)** and Masson staining **(D)** showed that the YSHS granule obviously decreased the accumulation of collagen fibers in glomerulus of ADR-induced FSGS model mice. **(E–G)** The results of IHC staining FN **(E)**, Vimentin **(F),** and α-SMA **(G)** showed that the YSHS granule obviously decreased the expression of FN, α-SMA, and Vimentin in the glomerulus of ADR-induced FSGS model mice. *N* = 6 in each group, the data were shown as mean ± SD, one-way ANOVA analysis followed by Tukey post-hoc tests as appropriate for multiple comparisons, ****p < 0.0001. scale bar: 50 µm.

Renal fibrosis is a fundamental pathological change in the development of a chronic kidney disease to the end stage, and it is involved in the glomerular sclerosis of FSGS model mice induced by ADR (Djudjaj and Boor, 2019). Therefore, we detected the expression of fibrosis-related markers in the kidneys of ADR-induced FSGS model mice treated with the YSHS granule by using Western blot, and the results showed that the expression of epithelial cell marker E-cadherin was up-regulated, the expression of mesenchymal cell markers N-cadherin, Vimentin, and α-SMA were obviously down-regulated, and the level of matrix metalloproteinase MMP9, which functions to degrade the extracellular matrix, was increased in the kidney, of YSHS granule-treated ADR-induced FSGS model mice compared with the FSGS model group (Figures 3A,B).

To further clarify whether the fibrosis of sclerotic glomeruli in ADR-induced FSGS model can be attenuated by the YSHS granule, we performed Sirius Red and Masson staining and the results showed that the deposition of collagen fibers in the glomeruli of ADR-induced FSGS model mice were obviously increased, while the deposition of collagen fibers in the glomeruli of FSGS model mice were significantly decreased by the YSHS granule treatment via intragastric administration (Figures 3C,D). In addition, the results of immunohistochemical staining showed that the YSHS granule not only significantly reduced the expression of Fibronectin (FN) (Figure 3E) and Vimentin (Figure 3F) in the glomeruli of FSGS model mice, but also significantly reduced the expression of α-SMA (Figure 3G) on the renal capsule wrapped outside the glomeruli.

These results indicated that the YSHS granule not only reduced the deposition of extracellular matrix, but also promoted the degradation of extracellular matrix by up-regulating the expression of MMP9, thus alleviating glomerular fibrosis induced by ADR, and effectively delaying the progress of chronic kidney diseases.

### Identification of the Targets of the Yi-Shen-Hua-Shi Granule Against Focal Segmental Glomerulosclerosis With Network Pharmacology

**FIGURE 4:**
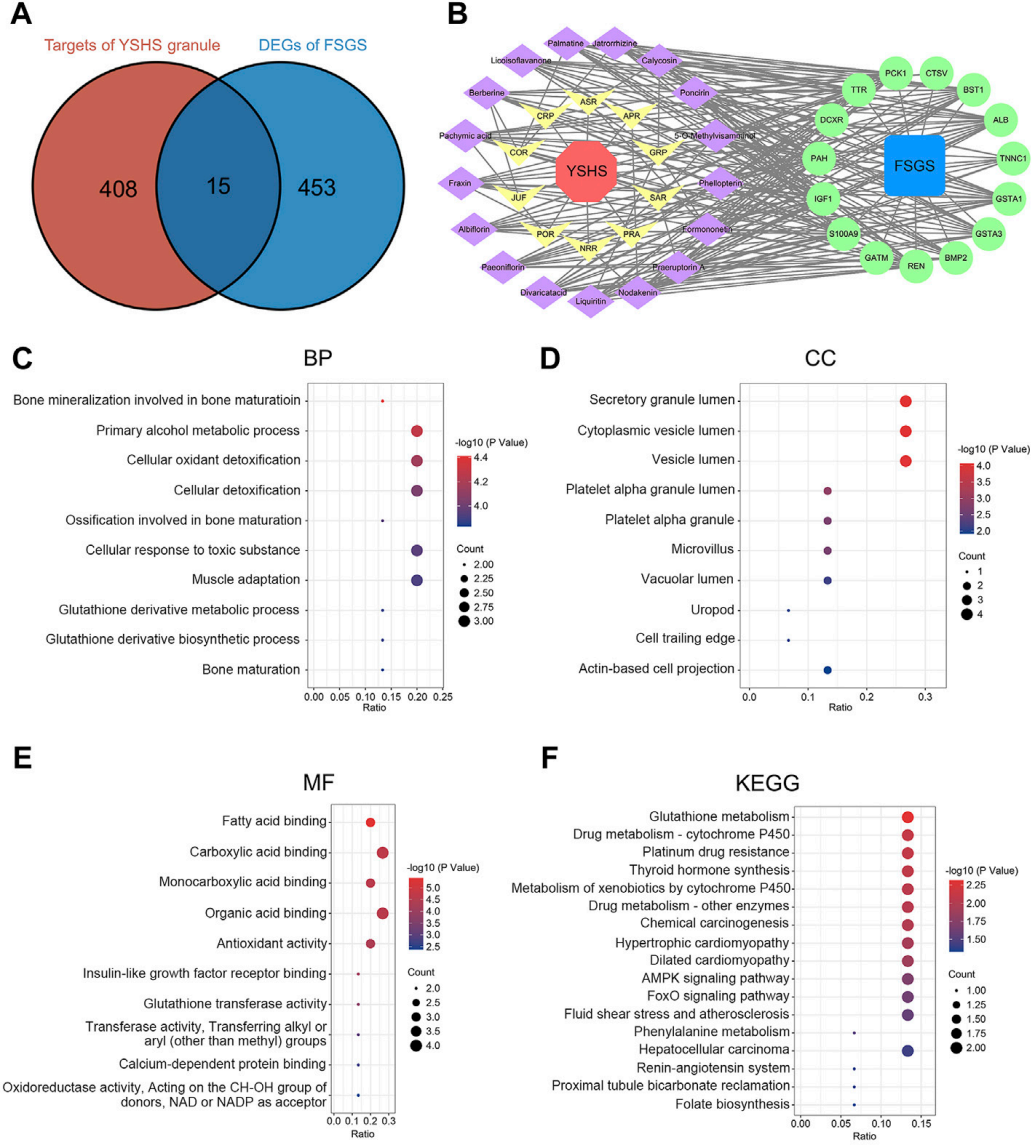
Identification and analysis of the targets of the YSHS granule against FSGS. **(A)** Venn diagram of the targets of the YSHS granule and DEGs of glomeruli from kidneys with FSGS. **(B)** A network of the active components of the YSHS granule and the targets of the YSHS granule against FSGS, which was constructed by using Cytoscape. **(C–E)** The top 10 items of BP **(C)**, CC **(D)** and MF **(E)** in GO enrichment of the targets of the YSHS granule against FSGS. **(F)** KEGG pathway enrichment of the targets of the YSHS granule against FSGS.

According to the reported literature, a total of 105 chemical components were detected in the YSHS granule by using high performance liquid chromatography coupled with electrospray ionization tandem quadrupole time-of-fight mass spectrometry (HPLC-Q-TOF/MS) (Chan et al., 2021). Then, these components were searched in the TCMSP platform and 17 active components were obtained with the criteria of OB ≥ 30% and DL ≥ 0.18, as shown in Table 2. The 17 active components were respectively entered into the PharmMapper server and 423 target proteins that interact with these components were found (Supplementary Table S1). According to the expression profiling data of glomeruli from kidneys with FSGS from GSE129973, a total of 468 DEGs were identified, which consist of 197 up-regulated and 271 down-regulated genes (Supplementary Table S2). A Venn analysis between 423 target proteins of the YSHS granule and 468 DEGs of FSGS revealed 15 overlapping targets, which may play crucial roles in the protective effects of YSHS on FSGS (Figure 4A). To visualize the relationship between the active components of the YSHS granule and the targets of the YSHS granule against FSGS, a component-target-disease network was constructed by using Cytoscape (Figure 4B). In the network, the red node (1) represents YSHS; the yellow nodes (10) represent Chinese herbal medicinal ingredients; the purple nodes (17) represent active chemical components; the blue node (1) represents FSGS; and the green nodes (Hopkins, 2007) represent the targets of the YSHS granule against FSGS.

**TABLE 2:** Active components of the YSHS granule.

Mol Id	Component	Formula	OB (%)	DL	Source
MOL006710	Fraxin	C_16_H_18_O_10_	36.76	0.42	NRR
MOL007004	Albiflorin	C_23_H_28_O_11_	30.25	0.77	PRA
MOL001924	Paeoniflorin	C_23_H_28_O_11_	53.87	0.79	PRA
MOL011737	Divaricatacid	C_16_H_16_O_7_	87.00	0.32	SAR
MOL004903	Liquiritin	C_21_H_22_O_9_	65.69	0.74	GRP
MOL004792	Nodakenin	C_20_H_24_O_9_	57.12	0.69	NRR, APR
MOL013079	Praeruptorin A	C_21_H_22_O_7_	46.46	0.53	SAR
MOL000392	Formononetin	C_16_H_12_O_4_	69.67	0.21	ASR, GRP
MOL002644	Phellopterin	C_17_H_16_O_5_	40.19	0.28	NRR, SAR
MOL011753	5-O-methylvisamminol	C_16_H_18_O_5_	37.99	0.25	SAR
MOL013276	Poncirin	C_28_H_34_O_14_	36.55	0.74	CRP
MOL000417	Calycosin	C_16_H_12_O_5_	47.75	0.24	ASR, GRP
MOL006397	Jatrorrhizine	C_20_H_20_NO_4_	30.44	0.75	COR
MOL000785	Palmatine	C_21_H_22_NO_4_	64.60	0.65	COR
MOL004885	Licoisoflavanone	C_20_H_18_O_6_	52.47	0.54	GRP
MOL001454	Berberine	C_20_H_18_NO_4_	36.86	0.78	COR, JUF
MOL000289	Pachymic acid	C_33_H_52_O_5_	33.63	0.81	POR

### The Characteristics and Functions of Prediction Targets

To investigate the biological functions of the predicted targets, GO and KEGG pathway enrichment analyses were conducted. The results of the GO enrichment analysis include three categories: biological process (BP), cellular component (CC), and molecular function (MF) (Supplementary Tables S3–S5). The top 10 items of BP, CC, and MF in GO enrichment were visualized via bubble charts (Figures 4C–E). These results indicated that the YSHS granule might be involved in cellular oxidant detoxification, antioxidant activity, fatty acid binding, and insulin-like growth factor receptor binding to play protective roles in FSGS. The KEGG enrichment analysis identified 17 pathways (Supplementary Table S6) and the results were visualized by bubble charts (Figure 4F). A previous study found that impaired glutathione metabolism might be related to the acceleration of renal fibrosis progression (You et al., 2020). It can be inferred that the YSHS granule might alleviate renal fibrosis in FSGS by restoring glutathione metabolism disturbance. Taken together, the results of the GO and KEGG pathway enrichment analyses supported that the 15 predicted targets had the characteristics and functions as the therapeutic targets of the YSHS granule for treating FSGS.

### The Expression Changes of the Predicted Targets in Focal Segmental Glomerulosclerosis Model Mice Treated With the Yi-Shen-Hua-Shi Granule

**FIGURE 5:**
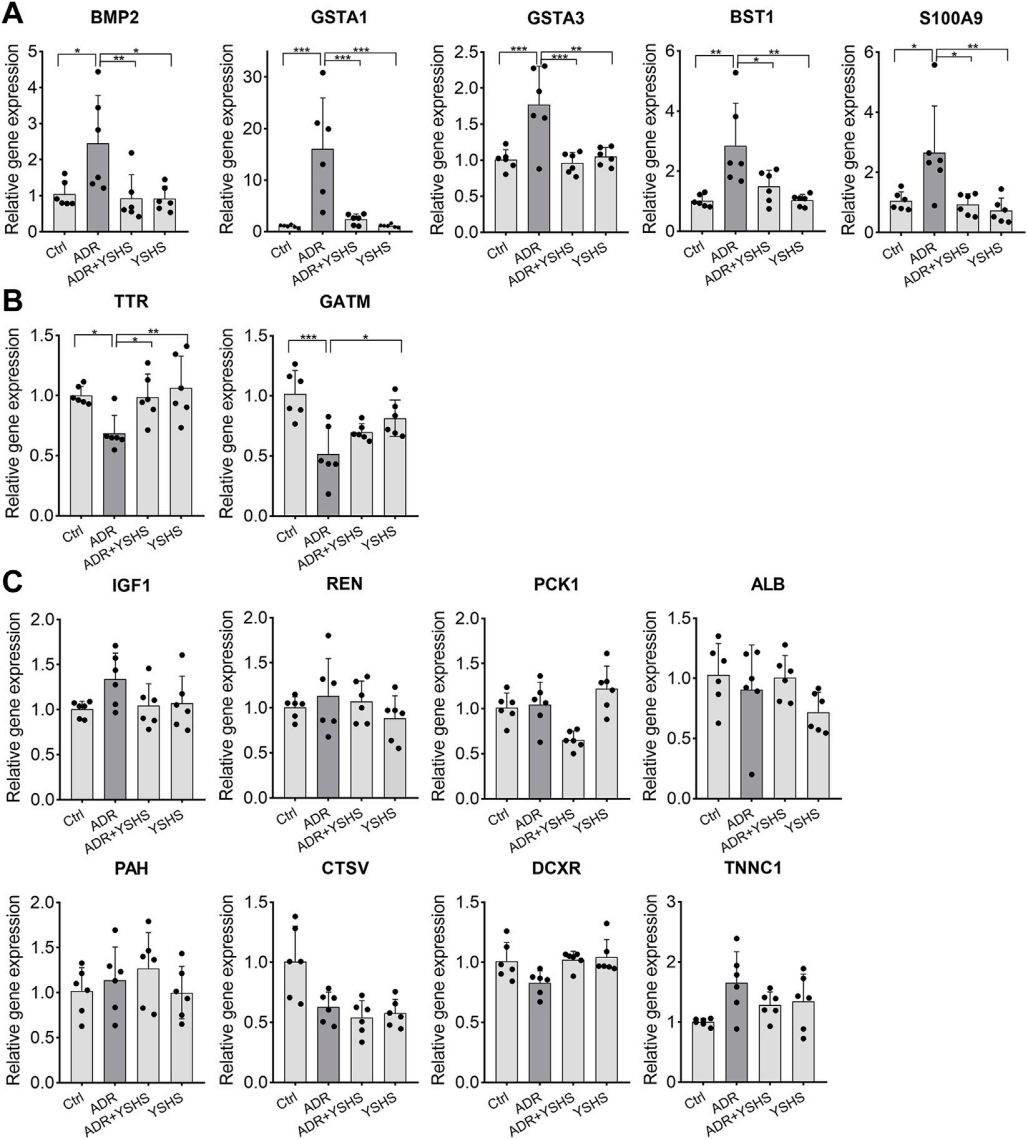
Effects of the YSHS granule on the expression of predicted target genes in the ADR-induced FSGS model. **(A–C)** Real-Time PCR was performed to analyze the expression of these predicted targets. YSHS down-regulated the expressions of Bmp2, Gsta1, Gsta3, Bst1, and S100a9 **(A)**, and up-regulated the expression of Ttr and Gatm **(B)**, while it did not affect the expressions of Igf1, Ren, Pck1, Alb, Pah, Ctsv, Dcxr, and Tnnc1 **(C)** in the ADR-induced FSGS model. *N* = 6 in each group, the data were shown as mean ± SD, one-way ANOVA analysis followed by Tukey post-hoc tests as appropriate for multiple comparisons, *p < 0.05, **p < 0.01, ***p < 0.001.

The expression changes of these 15 predicted target genes in FSGS model mice treated with the YSHS granule were evaluated by using real-time PCR assay. Results showed that the YSHS granule down-regulated the expressions of Bmp2, Gsta1, Gsta3, Bst1, and S100a9 (Figure 5A), and up-regulated the expressions of Ttr and Gatm (Figure 5B) in the kidney tissues of ADR-induced FSGS model mice, while there were no significant changes on the expressions of Igf1, Ren, Pck1, Alb, Pah, Ctsv, Dcxr, and Tnnc1. (Figure 5C).

### Yi-Shen-Hua-Shi Granule Inhibits the Activity of the BMP2/Smad Signaling Pathway

**FIGURE 6:**
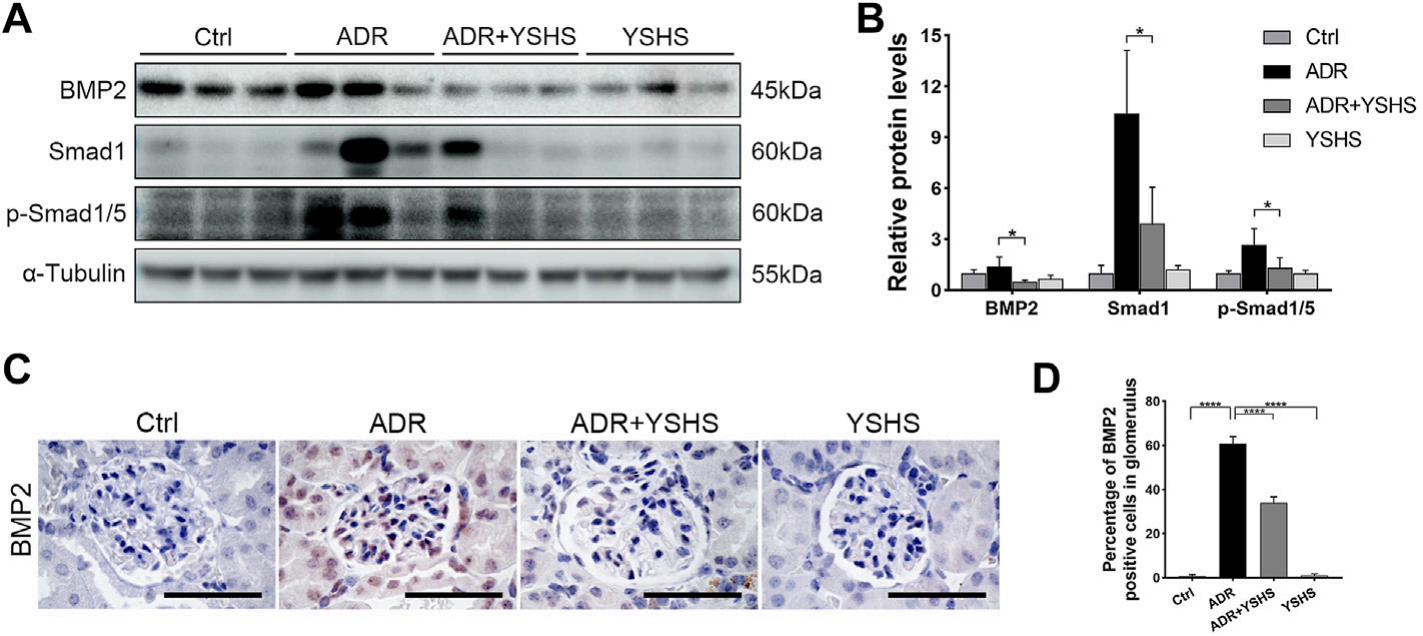
YSHS granule inhibits the BMP2/Smad signaling pathway. **(A)** The expression of BMP2, Smad1, and p-Smad1/5 were determined by Western blot. α-Tubulin was used as a loading control. **(B)** The quantification of the relative intensities of blots showed that the YSHS granule reduced the protein expression of BMP2, Smad1, and p-Smad1/5 in the ADR-induced FSGS model. **(C)** Representative IHC staining of BMP2 in the indicated groups. **(D)** The quantification of the BMP2 positive cells in glomerulus revealed that the YSHS granule obviously decreased the expression of BMP2 in ADR-induced FSGS model. *N* = 6 in each group, the data were shown as mean ± SD, one-way ANOVA analysis followed by Tukey post-hoc tests as appropriate for multiple comparisons, *p < 0.05, ****p < 0.0001. scale bar: 50 µm.

It is well known that BMP2, a member of the TGF-β superfamily, is involved in the fibrosis process in a variety of tissues and cells through the Smad signaling pathway (Wang et al., 2012; Wang and Wu, 2018; Hong et al., 2020; Su et al., 2020). The protein expression of BMP2 was up-regulated in the kidney tissues of ADR-induced FSGS model, while the YSHS granule significantly decreased the expression of BMP2 in the ADR-induced FSGS model (Figures 6A,B), which was consistent with the expression trend of Bmp2 mRNA detected by Real-time PCR (Figure 5A). Simultaneously, the YSHS granule also decreased the expressions of Smad1 and p-Smad1/5 in the kidney of the FSGS model (Figures 6A,B). In addition, the results of immunohistochemistry further demonstrated that the YSHS granule reduced the expression of BMP2 in glomeruli of the ADR-induced FSGS model (Figures 6C,D). These results suggest that the YSHS granule attenuates glomerular fibrosis in the ADR-induced FSGS model by inhibiting the activity of the BMP2/Smad signaling pathway.

## Discussion

ADR is a potent cytotoxic antitumor drug, which can simultaneously inhibit the synthesis of cellular DNA and RNA, induce cell apoptosis, and have an obvious toxic damage to the glomeruli and renal tubules, eventually leading to FSGS with proteinuria, hyperlipidemia, and other clinical manifestations (Qiao et al., 2018). The occurrence of proteinuria is caused by the destruction of the glomerular filtration membrane. When the kidney is damaged by ADR, podocytes, the most important part of the filtration membrane, will fall off after being stimulated. Then, the exposed basement membrane adheres to the wall of Bowman’s cyst and exudes plasma proteins. With the deposition of extracellular matrix, glomerulosclerosis is gradually formed (Wharram et al., 2005). In this study, we used the ADR-induced FSGS model in BALB/c mice to demonstrate that the YSHS granule can ameliorate renal injury in ADR-induced nephropathy, and found that both ADR-induced proteinuria and hyperlipidemia were significantly reduced after the intragastric administration of the YSHS granule to ADR-induced FSGS model mice.

Excessive deposition of extracellular matrix (ECM) is the core pathological change of renal fibrosis (Liu, 2006). Our results show that the YSHS granule reduced the accumulation of collagen fibers and FN in glomeruli caused by ADR, which are the main components of ECM. Meanwhile, the YSHS granule down-regulated the expression of α-SMA and Vimentin which were expressed in the activated myofibroblasts transformed from fibroblasts or epithelial cells (Mack and Yanagita, 2015; Djudjaj and Boor, 2019), and up-regulated matrix metalloproteinase MMP9 that degrades ECM (Yabluchanskiy et al., 2013). Here, we found that α-SMA was localized in the parietal layer of the renal capsule which is composed of a single layer of squamous epithelial cells, while Vimentin was mainly distributed in the glomerular interstitium, indicating that different types of epithelial cells underwent epithelial–mesenchymal transition under the stimulation of ADR. Therefore, the reduced expression of α-SMA and Vimentin in the ADR-induced FSGS model by the YSHS granule further suggested that the YSHS granule may prevent the progress of renal fibrosis in ADR-induced FSGS through the inhibition of EMT. However, which types of epithelial cells undergo EMT in the ADR-induced FSGS model and the mechanism of the YSHS granule inhibiting EMT still need to be further studied.

The most important feature of TCM is that it is composed of a variety of herbs and contains a variety of active ingredients. In this study, we identified 17 active ingredients from YSHS which is composed of 16 herbs. Some of these active ingredients have been reported to have anti-fibrotic effects. For example, paeoniflorin inhibited the epithelial–mesenchymal transition by downregulating the TGF-β/Smad signaling pathway, thereby improving pulmonary fibrosis and renal interstitial fibrosis (Zeng et al., 2013; Ji et al., 2016). Liquiritin prevented high fructose-induced myocardial fibrosis by inhibiting the NF-κB and MAPK signaling pathways (Zhang et al., 2016). Nodakenin inhibited UUO-induced renal fibrosis in mice and TGF-β1-treated renal epithelial cells, a classic model of cell fibrosis *in vitro*, by down-regulating the expression of Snail1 (Li et al., 2020). Formononetin activated the Nrf2/ARE signaling pathway through Sirt1, thereby ameliorating diabetic renal fibrosis (Zhuang et al., 2020). Calycosin ameliorated glomerulosclerosis and interstitial fibrosis in diabetic nephropathy by downregulating the IL33/ST2 signaling pathway (Elsherbiny et al., 2020). Berberine attenuated mesangial cell fibrosis via activating G protein-coupled bile acid receptor TGR5 and inhibiting the S1P2/MAPK signaling pathway (Yang et al., 2016). Although most of the active ingredients in the YSHS granule have been shown to have an anti-fibrotic effect in a variety of tissues, the anti-fibrotic effect and targets of these active ingredients in ADR-induced FSGS model remain unclear.

In this study, fifteen genes were predicted as the targets of the YSHS granule in the treatment of FSGS from the targets of the YSHS granule predicted from TCMSP and PharmMapper databases and the deferentially expressed genes from human glomeruli in kidneys with FSGS expression profiles from the GSE129973 dataset based on the network pharmacological analysis, which is an innovative method to predict the targets of TCM based on the interaction of “disease-gene-target-drug”. Then, seven genes, including BMP2, GSTA1, GSTA3, BST1, S100A9, TTR, and GATM, were identified as the targets of the YSHS granule in the treatment of ADR-induced FSGS by Real-time PCR analysis. Among these validated targets of YSHS, BMP2 belongs to the TGF-β superfamily, has a similar ligand structure, similar receptor-binding proteins, and similar downstream signaling cascades to TGF-β1 (Aashaq et al., 2022). Many researches demonstrated that BMP2 plays an important role in the occurrence, development, and outcome of renal interstitial fibrosis (Yang et al., 2009; Yang et al., 2011; Simone et al., 2012). Simone S et al. showed that BMP2 induced a profibrotic phenotype in adult human renal progenitor cells, such as increasing the expression of α-SMA, collagen I, and fibronectin protein (Simone et al., 2012), while, Yang YL et al. showed that BMP2 suppresses renal interstitial fibrosis (Yang et al., 2009; Yang et al., 2011). The role of BMP2 in renal fibrosis seems to be controversial. In our study, the expression of BMP2 was up-regulated in the ADR-induced FSGS model, while the expression of BMP2 was significantly inhibited after the intragastric administration of the YSHS granule. These data not only suggest that BMP2 is involved in the regulation of the fibrosis in the FSGS mouse model, but also suggest that BMP2 is one of the therapeutic targets in the treatment of fibrosis in the FSGS mouse model. However, the specific role of BMP2 in the regulation of fibrosis in the FSGS mouse model remains to be further clarified.

GSTA3, which is one of the most important members of the glutathione transferase family and is involved in the detoxification and cellular defense (Hayes et al., 2005), it was reported to attenuate renal interstitial fibrosis *in vivo* and *in vitro* by inhibiting the activity of the TGF-β1 signaling pathway (Xiao et al., 2016; Xiao et al., 2019) and inhibited liver fibrosis through the suppression of the MAPK and GSK-3β signaling pathways (Chen et al., 2019), suggesting that GSTA3 is an effective anti-fibrosis target. S100A9 was not only identified as a marker for idiopathic pulmonary fibrosis (Hara et al., 2012; Yamashita et al., 2021), but also aggravated dermal fibrosis induced by bleomycin in mice via activation of ERK1/2 MAPK and NF-κB pathways (Xu et al., 2018). In our study, we also found that the expression of S100A9 was up-regulated in the ADR-induced FSGS mouse model, indicating that S100A9 may promote glomerular fibrosis in the FSGS model. After intragastric administration of the YSHS granule, the expression of S100A9 decreased significantly, suggesting that it may be one of the effective targets for the YSHS granule to alleviate glomerular fibrosis in the FSGS model. GATM, which encodes the mitochondrial enzyme glycine amidinotransferase, is involved in creatine biosynthesis (Carney, 2018). Reichold, M. et al. showed that the accumulation of mutant GATM in the mitochondria of the proximal tubule, resulted in mitochondrial enlargement and elongation, eventually leading to renal tubular injury, renal Fanconi syndrome, and later in life, to fibrosis and progressive loss of renal function (Reichold et al., 2018), while another research showed that GATM knockout only caused neurological symptoms due to creatine deficiency but did not caused dysfunctions in renal and renal fibroses (Choe et al., 2013). Hence, whether GATM has an anti-fibrotic effect remains to be elucidated. The other three target genes (GSTA1, TTR, and BST1) have not been reported to be associated with fibrosis. Therefore, the role of these three targets in the treatment of renal fibrosis needs to be further clarified.

In summary, our research showed that the YSHS granule significantly improved the renal function and reduced the fibrosis in the glomeruli of ADR-induced FSGS model mice via suppressing of the BMP2/Smad signaling pathway. Our results indicated that the YSHS granule may be an effective drug to alleviate glomerular fibrosis in FSGS and BMP2 may be served as an effective therapeutic target in the treatment of renal fibrosis and FSGS.

## Data Availability

The datasets presented in this study can be found in online repositories. The names of the repository/repositories and accession number(s) can be found in the article/Supplementary Material.
