# Supplementary material for: Yi-Shen-Hua-Shi Granule Alleviates Adriamycin-Induced Glomerular Fibrosis by Suppressing the BMP2/Smad Signaling Pathway
Source: Front Pharmacol. 2022 Jun 15;13:917428. doi: 10.3389/fphar.2022.917428 (PMC9240271; doi:10.3389/fphar.2022.917428)
Supplement: Supplementary file 1 [file DataSheet1.DOCX]

**Yi-Shen-Hua-Shi** **granule alleviates adriamycin-induced glomerular fibrosis by suppressing BMP2/Smad signaling pathway**

Zhuojing Tan, Yachen Si, Yan Yu, Jiarong Ding, Linxi Huang, Ying Xu, HongXia Zhang, Yihan Lu, Chao Wang, Li Yuan and Bing Yu

**Supplementary Table1. Target proteins of active components in YSHS granule.**

| **Component** | **Target proteins** | | | | | | | | | |
| --- | --- | --- | --- | --- | --- | --- | --- | --- | --- | --- |
| Fraxin | BCHE | PGR | MIF | CES1 | IGF1R | ELANE | ADH5 | FNTA | F11 | EPHX2 |
|  | NUDT9 | AMY1A | APCS | MAPK14 | TNK2 | ARHGAP1 | PADI4 | GSTA1 | CTSG | PTK2 |
|  | CDK2 | BACE1 | AZGP1 | PTPN1 | BIRC7 | C8G | LYZ | KIT | BRAF | TPSB2 |
|  | GBA | CHEK1 | SRC | IMPA1 | CPB1 | PARP1 | CCL5 | SETD7 | STAT1 | APRT |
|  | PPIA | PNP | MMP8 | RHOA | HMGCR | FABP4 | HEXB | GSTT2B | CSNK2A1 | HPRT1 |
|  | DDX6 | LGALS7 | BAG1 | LGALS2 | RNASE4 | ITK | FKBP1B | SULT2B1 | GCK | AKT2 |
|  | TGFBR2 | F2 | CRAT | SYK | AR | TGM3 | HRAS | FABP6 | CD209 | RAN |
|  | GSTP1 | RTN4R | ATOX1 | LDHB | NCS1 | MET | NOS2 | CLEC4M | EEA1 | MMP1 |
|  | CCNA2 | AKT1 | PNPO | CASP7 | IMPDH2 | MTHFD1 | ADAM33 | MMP2 | FECH | ACE |
|  | CMA1 | QPCT | PDPK1 | PDE5A | ADAM17 | PAK6 | RAB11A | ARG2 | ACADM | PKLR |
|  | CHEK1 | DHFR | IGLV2-8 | CBR1 | ME2 | TGM2 | GSR | IL2 | CHIT1 | DPEP1 |
|  | CA12 | KDR | GALK1 | NMNAT3 | XIAP | GPI | CLK1 | NR1H4 | BLVRB | MAOB |
|  | FAP | CFD | LSS | ADK | OTC | ABO | SULT2A1 | C1S | TK1 | PPCDC |
|  | NR1H2 | F10 | SORD | REN | AMY2A | IGF1 | OAT | UMPS | FDPS | HMOX1 |
|  | PDE4B | BMP7 | PPARG | BCAT2 | CASP3 | ACP3 | S100A9 | ST14 | NOS3 | GSTA3 |
|  | CA1 | PLA2G10 | PDE3B | EPHB4 | REG1A | ESR2 | RAB5A | CTSF | KYAT1 | NDST1 |
|  | METAP1 | HK1 | KIF11 | TYMS | SHBG | MAPK8 | GP1BA | PLA2G2A | LTA4H | ARG1 |
|  | PIM1 | HSP90AA1 | CTNNA1 | SHMT1 | METAP2 | GNPDA1 | B3GAT1 | CTSS | ABL1 | CDC42 |
|  | CTSD | ANG | ALDH2 | ISG20 | ALB | TPH1 | HSPA1A | SERPINA1 | RNASE2 | HSP90AB1 |
|  | AURKA | PDE4D | PCK1 | LCK | DCK | IMPDH1 | NR1I2 | ZAP70 | KAT2B | GSTM2 |
|  | EGFR | CTSB | CDA | PLAU | TYMP | CDK5R1 | LGALS3 | TGFB2 | MME | GSTM1 |
|  | HCK | HSD11B1 | AHCY | BST1 | RHEB | TPI1 | F7 | GALE | NT5M | WARS1 |
|  | ICAM2 | MMP13 | ESRRG | RAC2 | MMP7 | SRM | MAPKAPK2 | PRKCQ | NMNAT1 | CDK7 |
|  | TREM1 | PAH | FGFR1 | FKBP1A | PKIA | HEXB | AMY1A | CBS | MMP16 | PRKACA |
|  | HSPA8 | CFB | PAK5 | MAN1B1 | CD1A | ABO | UCK2 | SDS | BHMT | ALAD |
|  | FCAR | CSNK1G2 | MMP12 | ANXA5 | DHODH | EPHA2 | JAK2 | MAPK1 | PLEKHA4 | PITPNA |
|  | PYGL | AKR1B1 | SOD2 | RNASE3 | HDAC8 | FHIT | NR3C2 | GART | HINT1 | GMPR |
|  | CLC | CTSK | ESR1 | GSK3B | CYP2C9 | PPARA | ALDOA | AGXT | PAPSS1 | NR3C1 |
|  | CA2 | DAPK1 | AMD1 | DPP4 | JAK3 | NQO1 | SELP | THRA | HAGH | VDR |
|  | MTAP | MMP3 | CDK6 | AKR1C3 | AKR1C2 | MMP9 | DTYMK | CSK | EIF4E |  |
| Albiflorin | PPARG | SULT2A1 | CCNA2 | ADH1B | RXRA | APCS | TNK2 | CTSF | RHEB | ACP3 |
|  | CA2 | SRC | REN | NQO2 | IGF1R | DAPK1 | PNMT | XIAP | C8G | HMOX1 |
|  | PPIA | RORA | F10 | AMY1A | ADH5 | LTA4H | ABO | IGF1 | GPI | ARSA |
|  | TTR | DHFR | MMP13 | TNNC1 | NR1H3 | ELANE | TGM3 | SULT2B1 | CCNT1 | HNMT |
|  | CFB | HSP90AA1 | DDX6 | CSNK2A1 | CDK6 | LCK | BLVRB | THRA | AKR1C1 | CLK1 |
|  | CA12 | FKBP1A | PLK1 | SORD | MMP7 | NR1I3 | JAK3 | F11 | S100A9 | KYAT1 |
|  | PNP | CA1 | ALB | PTPN1 | IGLV2-8 | NCOA5 | LSS | DPEP1 | RBP4 | C1S |
|  | TGFBR1 | ESRRG | ADH1C | PDE3B | BCAT2 | ATOX1 | BST1 | GSTT2B | ARG1 | GLO1 |
|  | MAPK14 | MAOB | CTSB | PAK5 | YARS1 | FNTA | BIRC7 | ZAP70 | ACADM | KIT |
|  | BACE1 | IMPA1 | EPHX2 | PAH | AMD1 | SETD7 | B3GAT1 | LDHB | RARA | INSR |
|  | HSP90AA1 | NQO1 | MMP8 | SYK | MDM2 | TYMS | RNASE3 | PADI4 | F7 | ABL1 |
|  | PDPK1 | CTSD | LGALS7 | MMP12 | PYGL | CD1A | CTSG | REG1A | HAGH | SELP |
|  | MAPK8 | ESR1 | CMA1 | DHODH | HMGCR | RHOA | AMY2A | MAPK1 | AGXT | TPI1 |
|  | MAPK10 | PDE4B | HSD17B1 | BMP7 | DPP4 | AHCY | CTNNA1 | MMP2 | PRKCQ | VDR |
|  | MMP3 | EGFR | GSK3B | MTHFD1 | CHIT1 | AKR1C2 | PCK1 | GNPDA1 | CSK | DTYMK |
|  | GBA | PIM1 | AKR1C3 | CASP3 | ISG20 | GSTA1 | LGALS2 | FHIT | ACE | AMY1A |
|  | BCHE | FAP | FGFR1 | CYP2C9 | CDA | PPARA | SDS | GM2A | ALDOA | OAT |
|  | GSTP1 | PDE4D | WAS | PDE5A | MAPKAPK2 | HCK | RNASE4 | FABP6 | OTC | SERPINA1 |
|  | TREM1 | PPARD | SEC14L2 | FGFR2 | PKIA | UCK2 | PDK2 | GALE | TPH1 | FDPS |
|  | NR1H2 | AR | PARP1 | ADAM17 | ST14 | TRAPPC3 | IMPDH2 | CBS | HPN | HRAS |
|  | RTN4R | CLPP | PIK3CG | NR3C2 | PLAU | RAB11A | ME2 | HSPA1A | MMP9 | CRABP2 |
|  | AURKA | HSPA8 | KDR | PNPO | CSNK1G2 | NR1I2 | SHMT1 | PRKACA | RAB5A | LGALS3 |
|  | CHEK1 | AKR1B1 | DCK | CRAT | CTSS | PAK6 | ALDH2 | PLAT | NOS2 | CCL5 |
|  | MTAP | SOD2 | ANG | HDAC8 | CTSK | HK1 | JAK2 | CALM1 | CPB1 | SRM |
|  | KIF11 | CDK2 | MIF | AKT1 | ITK | ERBB4 | CYP2C8 | RARG | HEXB | UMPS |
|  | TGFBR2 | F2 | CBR1 | C1R | SHBG | PLA2G2A | NCS1 | MAN1B1 | HEXB | LCN2 |
|  | CES1 | FCAR | METAP2 | ADK | PTPN11 | CASP7 | RAC2 | ABO | ARHGAP1 | LYZ |
|  | PGR | PLA2G10 | EPHB4 | AZGP1 | MET | FABP4 | HNF4G | GCK | MME | PIK3R1 |
|  | CLC | ANXA5 | METAP1 | BAG1 | HPGDS | THRB | FABP3 | FECH | NR3C1 | BHMT |
|  | CFD | HSD11B1 | CDK5R1 | NOS3 | NR1H4 | BRAF | IL2 | SULT1E1 | IMPDH1 |  |
| Paeoniflorin | KIF11 | CHEK1 | MIF | CYP2C9 | CDA | PDK2 | HPGDS | CSNK2A1 | REG1A | ARG1 |
|  | RORA | PDE4D | HSD11B1 | TTPA | CDK5R1 | C1R | NR1I2 | AGXT | PADI4 | TEK |
|  | AKR1C2 | SULT2A1 | CASP7 | ADK | PLA2G2A | AZGP1 | SHMT1 | SULT1E1 | ADAM17 | HEXB |
|  | MAOB | PIK3CG | NQO1 | PDE3B | ABO | HSPA8 | FECH | UCK2 | BHMT | PPP1CC |
|  | PIM1 | AKR1C3 | METAP2 | ESR2 | PTPN11 | DCK | CTSF | NR1I3 | HMOX1 | PDHB |
|  | BMP2 | HSP90AA1 | SYK | GSTA1 | THRB | DAPK1 | RARG | TNK2 | PPP5C | MAP2K1 |
|  | MMP3 | RTN4R | SOD2 | REN | AKR1C1 | BCAT2 | CSNK1G2 | RBP4 | BST1 | TPI1 |
|  | PPARD | CDK2 | AKR1B1 | ADH1C | CD1A | CSK | PAH | HK1 | FHIT | ALDH2 |
|  | AR | ESRRG | GBA | MMP8 | IMPA1 | HCK | BLVRB | RARA | BIRC7 | TPH1 |
|  | TTR | ALB | FKBP1A | PAK5 | FGFR2 | ADH5 | THRA | RNASE3 | NCS1 | GRB2 |
|  | CA2 | ESR1 | EPHX2 | CMA1 | ADH1B | MAPK1 | PYGL | ITGAL | HPN | KIT |
|  | CFD | KDR | FAP | FGFR1 | JAK3 | CYP2C8 | TYMP | RHEB | VDR | OAT |
|  | MAPK14 | PDPK1 | ANXA5 | ELANE | ERBB4 | FABP6 | XIAP | NOS3 | ACP3 | RARB |
|  | TGFBR1 | CCNA2 | LCK | CBR1 | AMY1A | DPP4 | SERPINA1 | TGM3 | B3GAT1 | GSTT2B |
|  | NR1H2 | GSTP1 | MET | PLK1 | ANG | CTSK | ATOX1 | HNMT | CRABP2 | LDHB |
|  | PPIA | CA1 | EPHB4 | PTPN1 | LGALS7 | DUSP6 | AHCY | F11 | MME | NOS2 |
|  | PPARG | MET | F10 | THRB | PDE5A | MTHFD1 | PRKACA | PLAU | ACADM | CLK1 |
|  | PGR | CLPP | TREM1 | HMGCR | CTSB | IGLV2-8 | CTSS | ABL1 | MMP7 | AKT2 |
|  | MAPKAPK2 | CTNNA1 | MDM2 | PRKACA | BMP7 | JAK2 | LTA4H | HNF4G | GCK | EIF4E |
|  | MAPK8 | PNP | NR1H3 | QPCT | TYMS | PRKCQ | LGALS2 | FABP7 | PLAT | HAGH |
|  | PDE4B | ADAM17 | LSS | PPARA | PNPO | ZAP70 | S100A9 | CTSG | OTC | GALE |
|  | DDX6 | HSD17B1 | CASP3 | YARS1 | ST14 | CALM1 | DPEP1 | PIK3R1 | RAC2 | HEXB |
|  | CA12 | AURKA | GSK3B | TNNC1 | CRAT | APCS | SULT2B1 | PAK6 | CBS | EPHA2 |
|  | BACE1 | DHFR | ITK | MMP12 | IGF1R | MMP2 | IGF1 | RNASE4 | SDS | MAN1B1 |
|  | SRC | CTSD | HDAC8 | F7 | DHODH | IL2 | AMY2A | PNMT | ABO | MMP9 |
|  | F2 | MTAP | PLA2G10 | PARP1 | AKT1 | AMD1 | GPI | PCK1 | RHOA | FABP5 |
|  | CFB | WAS | METAP1 | CES1 | BAG1 | BRAF | GM2A | RAB11A | ACE | SRM |
|  | BCHE | NQO2 | SHBG | NR1H4 | CDK6 | SETD7 | LCN2 | IMPDH2 | ME2 | ALDOA |
|  | EGFR | SEC14L2 | MMP13 | SORD | CHIT1 | FNTA | FABP4 | NR3C1 | TGFB2 | HSPA1A |
|  | MAPK10 | RXRA | NR3C2 | TRAPPC3 | PGF | FABP3 | ISG20 | CCNT1 | BCL2L1 |  |
| Divaricatacid | BMP2 | ANXA5 | MAPK8 | DHODH | UMPS | MET | CCL5 | THRA | AKT2 | HSP90AB1 |
|  | CA2 | ADAM17 | ARF1 | PTPN1 | ADH5 | NQO1 | PDK2 | LGALS2 | SETD7 | DPEP1 |
|  | CCNA2 | SRC | LGALS7 | MMP12 | PNMT | ACP3 | NR1H2 | BIRC7 | NMNAT3 | ARG2 |
|  | PPIA | KDR | MMP3 | HDAC8 | NMNAT1 | MAPK10 | LYZ | NOS2 | RAB5A | HEXB |
|  | PGR | GATM | F2 | NR3C2 | HK1 | TGFBR1 | PPARA | BHMT | FABP6 | S100A9 |
|  | AR | HSD17B1 | PIM1 | RAC2 | AHCY | ELANE | IL2 | MMP9 | FABP7 | SELP |
|  | CHEK1 | ANG | NQO2 | F10 | LDHB | CYP2C9 | ADK | CTSS | PPCDC | CDK7 |
|  | DDX6 | BMP7 | IGF1R | RHOA | MMP7 | LSS | MTHFD1 | PRKCQ | HAGH | PKLR |
|  | SNRPA | BCAT2 | PNP | RNASE3 | IMPDH2 | MAPKAPK2 | PLA2G2A | SRM | ACADM | STAT1 |
|  | GSTP1 | PPARG | PLA2G10 | CBR1 | TNK2 | ARHGAP1 | MAN1B1 | HINT1 | KYAT1 | GLO1 |
|  | CDK2 | FKBP1A | ADH1B | RNASE4 | C8G | TGM3 | PAK6 | PAPSS1 | SDS | GCDH |
|  | GBA | SHBG | PSPH | SULT2A1 | APCS | RARA | HCK | ABO | CHIT1 | HMGCR |
|  | BCHE | HSP90AA1 | RXRA | METAP2 | HPN | ITK | GPI | KIT | IMPDH1 | MAPK1 |
|  | CYP19A1 | B3GAT1 | ISG20 | HPGDS | EPHX2 | GALE | FDPS | ATIC | ZAP70 | EIF4E |
|  | CHEK1 | DAPK1 | CASP7 | CPB1 | CASP3 | FABP4 | HSPA1A | IGF1 | CRABP2 | NR3C1 |
|  | AURKA | ME2 | YARS1 | MMP8 | TPI1 | INSR | ADAM17 | CTSF | OTC | GSTO1 |
|  | RTN4R | CFB | AKT1 | DHFR | C1S | JAK3 | HRAS | FABP3 | DTYMK | RAF1 |
|  | GSR | CTNNA1 | MMP13 | PDPK1 | CBS | PTK2 | REN | TPH1 | HNF4G | CDC42 |
|  | PDE4D | MIF | IMPA1 | NCOA5 | GSTT2B | NR1I2 | AMY1A | NR1H4 | APRT | CTSG |
|  | EGFR | CES1 | CFD | PAH | LPA | NR1H3 | GSTA1 | TK1 | GSTA3 | OAT |
|  | ESRRG | C1R | CSNK2A1 | HSPA8 | BST1 | FGFR1 | RAB11A | ACE | XIAP | MMP16 |
|  | BACE1 | CTSB | TTR | PDHB | NCS1 | UCK2 | ALDOA | PLEKHA4 | MMP2 | DDX39B |
|  | AKR1C2 | PDE3B | KIF5B | CDK6 | LCK | TYMS | CDK5R1 | BRAF | CD1A | PRKACA |
|  | PDE4B | CSNK1G2 | MTAP | PLAU | F7 | FHIT | ARG1 | SULT1E1 | SULT2B1 | FECH |
|  | CA1 | PDE5A | PYGL | HSD11B1 | KIF11 | GCK | ABO | AGXT | TPSB2 | VDR |
|  | NOS3 | AMY1A | MAPK14 | DPP4 | CLK1 | THRB | PITPNA | EPHA2 | TEK | ARL5A |
|  | AKR1B1 | DUSP6 | AKR1C1 | CDA | PCK1 | PADI4 | HEXB | RAB9A | MAOB | GSTM1 |
|  | PPP5C | SOD2 | ALB | REG1A | SYK | AMY2A | MME | CANT1 | ERBB4 | ARF4 |
|  | AMD1 | EPHB4 | AKR1C3 | GSK3B | PKIA | HSPA1A | DCK | GNPDA1 | GP1BA | ARSA |
|  | SORD | ESR1 | PGF | CTSK | TTPA | SHMT1 | RHEB | JAK2 | KAT2B |  |
| Liquiritin | NUDT9 | PNP | AMD1 | TYMS | MAOB | PKIA | EPHX2 | NMNAT1 | KYAT1 | THRB |
|  | TTR | ALB | MAPK14 | CTSB | PDPK1 | HDAC8 | IMPDH2 | IL2 | PAK5 | LGALS3 |
|  | BCHE | APOA2 | PPARG | IGLV2-8 | HEXB | MET | RHOA | GP1BA | NR1I2 | JAK3 |
|  | CASP3 | STS | DHFR | NOS3 | MAN1B1 | ADAM17 | CSNK2A1 | TPI1 | TGM2 | NR3C1 |
|  | GSTP1 | CHIT1 | KDR | GPI | MMP12 | YARS1 | GNPDA1 | TK1 | ARHGAP1 | S100A9 |
|  | CA12 | CTSS | LGALS7 | EPHB4 | DCK | REN | HSD11B1 | AMY1A | ADK | ACAT1 |
|  | MTAP | NR3C2 | FKBP1A | SORD | CDA | ATOX1 | ABO | CD209 | BHMT | EIF4E |
|  | ESR1 | ESR2 | PDE4D | C1R | REG1A | AKR1C1 | RAC2 | NOS2 | PADI4 | MMP9 |
|  | CHEK1 | PAH | CTSK | LTA4H | IGF1 | SYK | AKR1C2 | CCL5 | GALE | FECH |
|  | CA2 | CHEK1 | MMP3 | F10 | F7 | TNK2 | RAB11A | CD1A | CLEC4M | ACADM |
|  | CDK2 | CFB | AZGP1 | DUSP6 | HSD17B1 | PTPN11 | HPN | TGFBR1 | CMA1 | HNMT |
|  | AR | BACE1 | BRAF | CES1 | HEXB | ALDOA | TYMP | THRA | MAPKAPK2 | PDHB |
|  | TGFBR2 | ANG | TNNC1 | ESRRG | DPP4 | CPB1 | PCK1 | GSTA1 | CDK5R1 | HINT1 |
|  | CA1 | DDX6 | MIF | AKT1 | ISG20 | PTPN1 | PARP1 | UCK2 | DTYMK | GSTO1 |
|  | PIM1 | EGFR | HSD17B11 | AURKA | SHMT1 | B3GAT1 | FABP5 | ITK | INSR | GM2A |
|  | CTSD | HK1 | NQO1 | PDE5A | RNASE4 | NCS1 | HCK | ABO | TPH1 | ZAP70 |
|  | CLC | MAPK10 | ALDH2 | PNPO | GSK3B | CCNT1 | ACP3 | PIK3CG | ST14 | SULT2B1 |
|  | AMY1A | METAP1 | BCAT2 | PPARD | OTC | CTNNA1 | NR1H4 | ELANE | OAT | PPCDC |
|  | FCAR | GALK1 | AKR1C3 | ANXA5 | MTHFD1 | BST1 | PAPSS1 | FABP4 | RAB5A | NT5M |
|  | F2 | PDE4B | FHIT | ADH5 | ME2 | ARF1 | HSPA1A | TREM1 | NR1H2 | CASP1 |
|  | CCNA2 | IMPA1 | IGF1R | GC | RHEB | CBR1 | HRAS | ERBB4 | BIRC7 | HAGH |
|  | HSP90AA1 | FAP | BAG1 | MMP8 | PLAU | C8G | CYP2C9 | UMPS | C1S | MMP2 |
|  | BMP7 | RTN4R | PDE3B | LDHB | MMP7 | NQO2 | LYZ | RBP4 | PKLR | FABP6 |
|  | GBA | AKR1B1 | CSNK1G2 | SRC | SOD2 | ADH1C | TRAPPC3 | AKT2 | HSP90AB1 | CDC42 |
|  | PYGL | LSS | SULT2A1 | GLO1 | PLA2G2A | LGALS2 | SEC14L2 | BLVRB | SELE | AGXT |
|  | CDK6 | FNTA | ADAM17 | KIF11 | LCK | PAK6 | SRM | PDK2 | ARF4 | EPHA2 |
|  | PPIA | CYP19A1 | DAPK1 | APCS | AMY2A | CFD | GCK | SELP | FDPS | GSTT2B |
|  | GSR | MAPK8 | CRAT | METAP2 | AHCY | HPGDS | SDS | PPARA | ABL1 | PLEKHA4 |
|  | HSPA8 | PGR | NMNAT3 | RNASE3 | PLK1 | TGM3 | MDM2 | HSPA1A | CLK1 | PRKCQ |
|  | SHBG | DHODH | MMP13 | FGFR1 | SERPINA1 | XIAP | HMGCR | RXRA | VDR |  |
| Nodakenin | ALB | FAP | NOS3 | C1R | PRKACA | PARP1 | REG1A | OTC | SULT1E1 | KIT |
|  | STS | MTAP | GSR | AKR1C3 | GSTA1 | ALDH2 | GPI | ELANE | FABP6 | EPHA2 |
|  | APOA2 | MAPK1 | CDA | HMGCR | HK1 | RHOA | PAK6 | METAP2 | SULT2B1 | FECH |
|  | TTR | AKR1B1 | PDPK1 | PPARD | SEC14L2 | MDM2 | CYP2C9 | TGM2 | FABP4 | GSTT2B |
|  | CLC | GBA | ADH1B | HPGDS | AMD1 | ADK | PPARA | CCNT1 | BHMT | GLO1 |
|  | HCK | FKBP1A | KDR | CHIT1 | REN | AHCY | ME2 | CDK5R1 | IGF1 | NMNAT1 |
|  | PIM1 | CA1 | LGALS7 | FNTA | CASP7 | LDHB | AKR1C2 | NOS2 | LGALS3 | APRT |
|  | GSTP1 | PIK3CG | DPP4 | TYMP | ATOX1 | FABP5 | FGFR2 | UCK2 | MAPKAPK2 | MAP2K1 |
|  | TREM1 | PGR | BAG1 | MMP3 | CSNK1G2 | HPN | RHEB | CRABP2 | PAPSS1 | GSTA3 |
|  | PPIA | HSD17B11 | CRAT | TNNC1 | BST1 | ALDOA | NCS1 | IL2 | F7 | HSPA1A |
|  | BCHE | FCAR | SRC | PTPN1 | PDE5A | CDK6 | ABO | HEXB | FDPS | AGXT |
|  | EPHB4 | GC | HSP90AA1 | MMP8 | YARS1 | RAB11A | DCK | TGM3 | CTSG | CBS |
|  | AR | CA12 | CSNK2A1 | DHODH | LCK | SYK | WAS | TPI1 | HNF4G | GP1BA |
|  | CA2 | ESR1 | EPHX2 | MAPK14 | PTPN11 | TPH1 | MTHFD1 | BRAF | CCL5 | PLEKHA4 |
|  | NR1H2 | CTSD | PNP | MMP13 | CTNNA1 | XIAP | RNASE4 | BIRC7 | CYP2C8 | PRKCQ |
|  | PDE4D | DAPK1 | MIF | APCS | HSD17B1 | AMY2A | JAK2 | GCK | MME | CD1A |
|  | ESR2 | ADAM17 | ESRRG | NR3C2 | PCK1 | TNK2 | FHIT | LGALS2 | RBP4 | THRB |
|  | NQO2 | LSS | F10 | HSD11B1 | TTPA | PAK5 | PADI4 | LYZ | MMP9 | SRM |
|  | PDE4B | EGFR | CTSB | IGF1R | PLA2G2A | AKR1C1 | ITK | CLK1 | BLVRB | HRAS |
|  | CDK2 | MAPK8 | PDE3B | SORD | ADH5 | TRAPPC3 | SHMT1 | THRA | DTYMK | GSTM2 |
|  | CHEK1 | SHBG | ST14 | CTSK | ISG20 | AKT1 | PDK2 | FABP3 | ZAP70 | IMPDH1 |
|  | PPARG | ANG | PYGL | CFB | PLAU | B3GAT1 | HEXB | ABO | ERBB4 | S100A9 |
|  | AURKA | ADH1C | RORA | F2 | MMP12 | RNASE3 | JAK3 | KYAT1 | AMY1A | NMNAT3 |
|  | TGFBR2 | ANXA5 | AZGP1 | SULT2A1 | MAOB | IMPDH2 | RXRA | CSK | DPEP1 | SETD7 |
|  | CASP3 | PAH | GALK1 | NQO1 | MMP7 | ADAM17 | GM2A | SERPINA1 | PPCDC | TK1 |
|  | CHEK1 | TYMS | CYP19A1 | MAN1B1 | CBR1 | MET | ACP3 | EIF4E | ITGAL | SDS |
|  | CTSV | AMY1A | BACE1 | GSK3B | BMP7 | CES1 | NR1H3 | NR1I2 | AKT2 | GALE |
|  | CCNA2 | DHFR | KIF11 | FGFR1 | SOD2 | HDAC8 | SELP | MMP2 | HAGH | HINT1 |
|  | METAP1 | CFD | PLA2G10 | MAPK10 | BCAT2 | NR1H4 | RAB5A | C1S | CTSF | ARG2 |
|  | IMPA1 | HSPA8 | CTSS | TGFBR1 | C8G | RAC2 | UMPS | ACADM | GNPDA1 |  |
| Praeruptorin A | MMP13 | MAPKAPK2 | EGFR | LCK | SORD | CYP2C9 | HCK | MMP2 | CHIT1 | SRM |
|  | STS | CMA1 | MIF | CHEK1 | PLAU | RBP4 | CSK | FHIT | GSTT2B | PIK3R1 |
|  | MAPK1 | NQO2 | IGF1R | ESRRG | WAS | DUSP6 | REG1A | SERPINA1 | CLK1 | ARG1 |
|  | FKBP1A | AR | CFD | HSD17B1 | XIAP | ISG20 | IGF1 | AMY1A | IMPDH2 | OTC |
|  | TREM1 | MAOB | RXRA | PTPN11 | FABP4 | ACP3 | MMP7 | ADAM17 | MME | C1S |
|  | ALB | CCNA2 | PDE5A | ESRRA | AHCY | EPHX2 | FABP7 | LCN2 | LGALS2 | EPHA2 |
|  | PIM1 | PDE4D | NQO1 | ADH1C | ME2 | TRAPPC3 | PGF | HNF4G | PITPNA | LYZ |
|  | MAPK10 | GC | PDPK1 | MET | TTPA | ACE | DHFR | GM2A | RXRB | LGALS3 |
|  | CFB | PPARG | ANG | KDR | ERBB4 | FGFR1 | RNASE3 | RARG | VDR | PPP1CC |
|  | PPIA | ANXA5 | CDK5R1 | PCK1 | NOS3 | FABP5 | PLAT | DCK | ALAD | CDK7 |
|  | PNP | CHEK1 | PDE4B | CALM1 | NR1H4 | PRKCQ | PADI4 | THRA | GCK | TPSB2 |
|  | CASP7 | BACE1 | GSK3B | REN | ADK | ELANE | FECH | SULT2B1 | PLEKHA4 | ATIC |
|  | CES1 | MAPK14 | BCAT2 | MDM2 | GSR | PNMT | BIRC7 | SDS | F11 | PCTP |
|  | GSTP1 | ADAM17 | PTPN1 | FGFR2 | HPGDS | YARS1 | IL2 | DPEP1 | NOS2 | TGFB2 |
|  | TTR | SRC | PDE3B | SEC14L2 | MTHFD1 | SULT1E1 | ITGAL | CTSF | PRKACA | FOLH1 |
|  | CA2 | SHBG | METAP2 | HDAC8 | ESR2 | THRB | CCL5 | S100A9 | AGXT | RAB5A |
|  | KIF11 | CDK2 | PPARD | ADH5 | CBR1 | JAK2 | RARA | GPI | RARB | ARF4 |
|  | NR1H2 | ESR1 | HSD11B1 | PLA2G2A | NCS1 | C8G | ABO | HNMT | KIT | HINT1 |
|  | F2 | PGR | HSPA8 | PRKACA | SOD2 | PAK5 | FABP3 | SETD7 | HSP90AB1 | PKLR |
|  | APOA2 | APCS | CTNNA1 | PAH | AURKA | CDK6 | TGM3 | ACADM | FKBP1B | GSTM1 |
|  | THRB | DHODH | F10 | SYK | PPP5C | RNASE4 | HEXB | MAP2K1 | PAK6 | NT5M |
|  | BMP2 | AKR1B1 | MMP8 | F7 | LDHB | NR1I2 | BST1 | PAPSS1 | ADAM33 | NMNAT3 |
|  | MAPK8 | NR3C2 | ITK | PARP1 | MMP12 | PPARA | CYP2C8 | NR3C1 | BHMT | WARS1 |
|  | AKR1C2 | PLK1 | JAK3 | HMGCR | LSS | MMP9 | TPH1 | NMNAT1 | STAT1 | BCL2L1 |
|  | BCHE | TGFBR1 | FNTA | EPHB4 | ZAP70 | CBS | ALDOA | AKT2 | FDPS | HPRT1 |
|  | PIK3CG | IGLV2-8 | AMD1 | MTAP | NR1H3 | BLVRB | PSAP | DTYMK | HMOX1 | PFKFB1 |
|  | CASP3 | LGALS7 | HSP90AA1 | CDA | PYGL | ABO | CRABP2 | TEK | OAT | GSTA3 |
|  | MMP3 | SULT2A1 | DPP4 | CTSK | PDK2 | NR1I3 | SHMT1 | ALDH2 | CDC42 | EIF4E |
|  | DDX6 | PLA2G10 | TYMS | CTSG | BRAF | CTSS | UCK2 | MAN1B1 | ABL1 | HRAS |
|  | CTSB | RORA | AKR1C3 | AKR1C1 | GSTA1 | TPI1 | FABP6 | LTA4H | HAGH |  |
| Formononetin | ESR1 | PDPK1 | HPGDS | CASP3 | RXRA | FABP6 | FABP3 | BIRC7 | RAB9A | RXRB |
|  | PIM1 | GSR | CDK6 | TNK2 | GCK | CBS | HMGCR | NOS2 | HNMT | UAP1 |
|  | ESR2 | ESRRG | AKR1C3 | CBR1 | SULT1E1 | AURKA | LYZ | TPH1 | ERI1 | HINT1 |
|  | CES1 | PNP | SULT2A1 | AMD1 | CRABP2 | REN | DPEP1 | KAT2B | INSR | BCAT2 |
|  | CDK2 | FGFR1 | ADH5 | MET | RAB11A | XIAP | ABL1 | RBP4 | ADAM33 | TPSB2 |
|  | AR | EGFR | MAPK8 | NQO1 | FABP4 | MME | HAGH | TGFB2 | EEA1 | BTK |
|  | CA2 | PDE4B | AKR1C1 | IMPA1 | GPI | PAH | FABP7 | SELE | EIF4E | DAPK1 |
|  | ALB | SHBG | SOD2 | HDAC8 | SULT2B1 | NMNAT1 | OTC | NT5M | CASP1 | ATIC |
|  | STS | HSD17B1 | PLAU | NR1H3 | PRKACA | MMP12 | RARG | IMPDH2 | MMP1 | MAP2K1 |
|  | APOA2 | HSD11B1 | BACE1 | ELANE | THRA | GSK3B | FECH | CDC42 | DDX39B | AKT1 |
|  | PGR | NR3C2 | NQO2 | METAP2 | GSTT2B | MMP2 | HPRT1 | HCK | FKBP1B | PFKFB1 |
|  | CFB | GC | CTSK | RNASE3 | CTSS | CCL5 | MMP9 | PAPSS1 | DUT | RAC1 |
|  | MMP13 | TTR | PTPN11 | UMPS | ABO | CDK7 | OAT | GSTM2 | GLO1 | AK1 |
|  | HSPA8 | PCK1 | REG1A | NR1H2 | MAOB | ARG1 | ADK | F11 | ARL5A | TRDMT1 |
|  | F2 | MIF | KDR | SEC14L2 | FKBP1A | TGM3 | GSTA1 | CTSB | RARB | TTPA |
|  | DUSP6 | DCK | NR1H4 | F10 | THRB | RAB5A | GSTP1 | BRAF | HADH | FKBP3 |
|  | ANG | NOS3 | DHODH | PADI4 | DTYMK | GCDH | MTAP | GSTA3 | GART | RFK |
|  | MMP8 | CCNA2 | KIF11 | TPI1 | HSPA1A | SRC | HNF4G | PLA2G2A | FOLH1 | TAP1 |
|  | HSP90AA1 | HSP90AA1 | AKR1B1 | MAPK10 | SERPINA1 | RARA | MAPKAPK2 | PPP1CC | FGG | PROCR |
|  | FNTA | ALDH2 | ERBB4 | DHFR | LCK | BHMT | PDK2 | LTA4H | PNMT | CRYZ |
|  | ADAM17 | PDE4D | PTPN1 | PRKACA | SDS | SYK | HEXB | RNASE2 | WARS1 | F7 |
|  | DPP4 | HK1 | UCK2 | TGFBR1 | IGF1 | NR3C1 | KIT | BST1 | GP1BA | RAP2A |
|  | CHEK1 | HPN | SHMT1 | PDE5A | JAK2 | ACP3 | PPARG | GSTM1 | GMPR | SULT1A1 |
|  | MAPK14 | CTNNA1 | MMP3 | TRAPPC3 | JAK3 | VDR | TEK | AMY2A | APAF1 | SIRT5 |
|  | PYGL | AHCY | CYP2C9 | NR1I2 | CHIT1 | S100A9 | HRAS | RORA | ARL5B |  |
| Phellopterin | KIF11 | TTR | CDK2 | CDA | WAS | RBP4 | GCK | CTSF | PIK3R1 | WARS1 |
|  | MAPKAPK2 | APOA2 | PDE3B | MET | HPN | LGALS3 | ADH1C | ARG1 | CASP3 | FKBP1B |
|  | MMP3 | NR3C2 | METAP2 | FABP4 | XIAP | MTAP | AKT2 | TK1 | RARA | GSTA3 |
|  | MAPK10 | CTSD | MIF | NQO1 | MMP12 | RARG | HCK | BRAF | OTC | APAF1 |
|  | MAPK1 | CCNA2 | RXRA | HSD11B1 | SORD | PYGL | PRKCQ | MMP2 | MMP9 | NOS2 |
|  | PIK3CG | PDE4B | HSPA8 | PTPN1 | EPHX2 | ESRRA | RNASE3 | PITPNA | TPH1 | PKLR |
|  | AR | GC | IGLV2-8 | HMGCR | MMP7 | PPARA | ACADM | PLEKHA4 | TPSB2 | NT5M |
|  | F2 | ADAM17 | TGFBR1 | KDR | CCNT1 | IMPDH2 | KIT | AKR1C2 | HMOX1 | AMY2A |
|  | ESR1 | SULT2A1 | PKIA | PCK1 | LSS | DCK | NR3C1 | CBS | ALAD | GSTM2 |
|  | TREM1 | ESRRG | MMP13 | CALM1 | DPEP1 | CSK | ISG20 | GPI | RXRB | RNASE2 |
|  | CES1 | PGR | PDE5A | DHODH | SULT2B1 | TEK | TGM3 | IMPDH1 | PAK6 | KAT2B |
|  | PPIA | FKBP1A | ANXA5 | GSTA1 | LTA4H | FABP7 | BLVRB | RARB | ARHGAP1 | RAB9A |
|  | CHEK1 | MAPK14 | CTNNA1 | GSTP1 | CBR1 | PADI4 | SDS | LCN2 | AMD1 | HPRT1 |
|  | MAPK14 | MAOB | BCAT2 | CYP2C9 | PTPN11 | ELANE | HNF4G | AGXT | HEXB | HINT1 |
|  | PIM1 | HSD17B1 | PPARD | CDK6 | ERBB4 | NR1H3 | TPI1 | F7 | STAT1 | ADAM33 |
|  | CA2 | IGF1R | FNTA | HDAC8 | ADH5 | ACP3 | SERPINA1 | CLK1 | CHIT1 | OAT |
|  | NQO2 | SHBG | REN | SOD2 | YARS1 | UCK2 | S100A9 | CRABP2 | FDPS | NMNAT3 |
|  | CYP19A1 | PLA2G10 | LCK | ADK | LGALS7 | ABO | FECH | ITGAL | ABO | ARL5A |
|  | CFB | NOS3 | AKR1C1 | GSK3B | FABP3 | PRKACA | HNMT | ACE | PCTP | GRB2 |
|  | PDE4D | SRC | CTSB | TTPA | SEC14L2 | DHFR | THRA | F11 | CCL5 | BTK |
|  | CMA1 | CDK5R1 | PLK1 | ITK | NR1H4 | NR1I2 | IGF1 | BHMT | BCL2L1 | INSR |
|  | THRB | HSP90AA1 | FGFR1 | FGFR2 | PLAT | CD1A | DTYMK | PPP1CC | CYP2C8 | HADH |
|  | BCHE | CFD | MTHFD1 | PLAU | FABP6 | BIRC7 | CTSG | ABL1 | RAB5A | DUT |
|  | MAPK8 | PDPK1 | ANG | JAK3 | PDK2 | ST14 | VDR | IL2 | EIF4E | ERI1 |
|  | CASP7 | GSR | AKR1C3 | MDM2 | FABP5 | MAP2K1 | GSTT2B | MAN1B1 | LYZ | TRDMT1 |
|  | NR1H2 | NCOA5 | PPARG | PARP1 | NR1I3 | CTSS | NMNAT1 | UMPS | CD209 | DCXR |
|  | STS | EGFR | RORA | TYMS | ADAM17 | AMY1A | MME | TGFB2 | PAPSS1 | RND3 |
|  | ALB | DPP4 | AURKA | SYK | PAH | ZAP70 | PAK5 | FOLH1 | CASP1 | GMPR2 |
|  | EPHB4 | MMP8 | BACE1 | HPGDS | SULT1E1 | TRAPPC3 | JAK2 | HSP90AB1 | GLO1 | FKBP3 |
|  | AKR1B1 | F10 | CTSK | PLA2G2A | SHMT1 | BST1 | PSAP | SETD7 | CDK7 |  |
| 5-O-Methylvisamminol | F2 | DAPK1 | DUSP6 | SRC | ADH5 | BST1 | PITPNA | RAB9A | CHIT1 | DDX39B |
|  | CA2 | SHBG | MTAP | F10 | GSK3B | BHMT | MAPK1 | SULT2B1 | PADI4 | CDK7 |
|  | BCHE | BCAT2 | LSS | HSD17B1 | CBS | LCK | ARG1 | LGALS3 | GSTA3 | NT5M |
|  | CES1 | CHEK1 | AMD1 | PGF | MMP7 | TGFBR1 | THRA | MMP12 | PPP1CC | GSTM1 |
|  | BMP2 | PPP5C | HPGDS | ESR2 | NR1H3 | HSPA1A | JAK3 | MAN1B1 | ITK | GSTM2 |
|  | PPIA | DHFR | EPHB4 | AKR1C2 | TRAPPC3 | GPI | AMY1A | HEXB | HEXB | ERI1 |
|  | MAPK10 | C1R | ESRRG | ADH1C | ADK | IL2 | SRM | SELP | RARA | TGFB2 |
|  | BACE1 | PPARD | PYGL | PTPN11 | PKIA | LYZ | NMNAT1 | GLO1 | ABL1 | HSP90AB1 |
|  | GBA | ADH1B | YARS1 | PDE3B | ABO | THRB | FGFR1 | FOLH1 | GCDH | SELE |
|  | MAPK8 | KDR | MAPK14 | BMP7 | PDE5A | BIRC7 | FABP6 | ARHGAP1 | FDPS | BCL2L1 |
|  | PNP | CTSB | AKR1B1 | NR3C2 | PARP1 | CCL5 | XIAP | IMPDH1 | FECH | TK1 |
|  | PIK3CG | PDE4B | PTPN1 | AURKA | GSTA1 | WAS | TTPA | HNMT | ARL5A | WARS1 |
|  | PLK1 | TYMS | EPHX2 | HSPA8 | ACP3 | TGM3 | ACADM | SDS | F11 | CTSF |
|  | AR | AKR1C3 | HSP90AA1 | HK1 | CYP2C9 | TPI1 | HRAS | PLA2G2A | RNASE2 | ZAP70 |
|  | MMP3 | PPARG | SORD | AHCY | JAK2 | RBP4 | CTSS | DPEP1 | GRB2 | CASP3 |
|  | CCNA2 | ADAM17 | F7 | NQO2 | HCK | SHMT1 | SULT1E1 | NOS2 | LCN2 | INSR |
|  | CFB | PGR | RORA | PAH | AMY1A | CRABP2 | RAB5A | NR3C1 | KIT | RARB |
|  | PDE4D | DHODH | CTSK | HDAC8 | DPP4 | HMGCR | REN | TPH1 | FGG | DUT |
|  | CYP19A1 | TTR | NR1H4 | FGFR2 | DCK | RAB11A | GNPDA1 | OTC | MAP2K1 | CASP1 |
|  | GSTP1 | PIM1 | KIF11 | NQO1 | MET | BRAF | PPCDC | PPARA | FKBP1B | PRKCQ |
|  | CDK2 | LGALS7 | PDPK1 | ACE | UCK2 | CYP2C8 | LTA4H | DTYMK | MMP16 | RXRB |
|  | ESR1 | ANXA5 | SEC14L2 | METAP2 | PCK1 | ATOX1 | ERBB4 | RARG | MAPKAPK2 | EIF4E |
|  | ANG | RXRA | SOD2 | CBR1 | ELANE | MMP2 | PLEKHA4 | AKT2 | KAT2B | TRDMT1 |
|  | GSR | PDPK1 | ISG20 | MMP8 | GCK | FABP3 | HAGH | EPHA2 | VDR | ADAM33 |
|  | EGFR | CTNNA1 | HSD11B1 | SYK | MTHFD1 | MME | SERPINA1 | TEK | STAT1 | MMP1 |
|  | MMP13 | SULT2A1 | CASP7 | GSTT2B | PLAU | PDK2 | MMP9 | FABP7 | PNMT | ITPKA |
|  | MAOB | ALB | AKR1C1 | NR1I2 | B3GAT1 | FKBP1A | RHEB | ARG2 | NMNAT3 | GMPR2 |
|  | NOS3 | FNTA | CDK6 | IMPDH2 | NR1H2 | IGF1 | CLK1 | ATIC | HINT1 | RAP2A |
|  | CFD | PLA2G10 | RHOA | CTSG | RNASE3 | HPN | ABO | PRKACA | HPRT1 | PKLR |
|  | MIF | IGF1R | CDA | REG1A | UMPS | HNF4G | SETD7 | AMY2A | HADH |  |
| Poncirin | TTR | HSPA8 | SOD2 | AMD1 | PAK6 | OTC | FABP5 | MET | IL2 | DTYMK |
|  | HCK | PDE4B | IMPA1 | CHIT1 | NQO1 | CBR1 | AKR1C1 | PPARA | AKR1C2 | MMP2 |
|  | CLC | APOA2 | ANXA5 | HSD17B1 | PLAU | RNASE4 | HSD11B1 | PAPSS1 | KYAT1 | MMP9 |
|  | CA2 | ICAM2 | ANG | ADAM17 | PKIA | ATOX1 | ABO | AGXT | BIRC7 | EPHA2 |
|  | DDX6 | PIM1 | CMA1 | ALDH2 | B3GAT1 | CCL5 | NQO2 | SEC14L2 | TK1 | CDK5R1 |
|  | CA1 | CDK2 | C1R | AZGP1 | TGFBR1 | RNASE3 | UCK2 | IMPDH1 | RBP4 | BHMT |
|  | BCHE | MAPK14 | FABP4 | ESRRG | EPHB4 | PLK1 | ARF1 | F7 | FDPS | KPYR |
|  | TREM1 | FCAR | SRC | RXRA | ITK | HMGCR | HRAS | CCNT1 | BLVRB | NR1H3 |
|  | MTAP | AKR1B1 | TNNC1 | FNTA | F10 | LCK | LYZ | HPGDS | GALE | SULT1E1 |
|  | PNP | BMP7 | CCNA2 | TYMP | TPI1 | XIAP | LDHB | ADAM17 | HEXB | MDM2 |
|  | F2 | MAPK1 | SHBG | CDA | HEXB | GSK3B | PLAT | GSTM1 | UMPS | KAT2B |
|  | FKBP1A | NR1H2 | MTHFD1 | PPARG | ELANE | FGFR1 | ZAP70 | HAGH | CTSS | CTSG |
|  | CTSV | CFB | PLA2G10 | CTSK | ABO | LSS | ADH5 | ARG2 | ARSA | CDC42 |
|  | CHEK1 | AR | AMY1A | KIF11 | SERPINA1 | RHOA | TGM3 | PDK2 | THRB | NR1I2 |
|  | PPIA | ESR2 | BAG1 | CTSB | CSNK2A1 | SYK | MMP7 | CYP2C9 | FABP6 | KIT |
|  | TGFBR2 | AKR1C3 | PDE4D | DPP4 | AKT1 | HSPA1A | EPHX2 | IGF1 | NMNAT1 | PRKCQ |
|  | FAP | BACE1 | DAPK1 | SULT2A1 | TNK2 | PTPN11 | ADK | HDAC8 | THRB | RNASE2 |
|  | GSTP1 | MAPK10 | CDK6 | GC | SORD | RAC2 | CBS | WAS | NR1H4 | PSAP |
|  | RTN4R | CES1 | CASP3 | PDE5A | AHCY | RAB5A | GSTT2B | AMY1A | NT5M | SELE |
|  | HSP90AA1 | PGR | CRAT | DHODH | TYMS | IMPDH2 | GPI | ITGAL | GSTA1 | MMP1 |
|  | GBA | HSD17B11 | PNPO | NCS1 | METAP2 | PPARD | CPB1 | SELP | GART | STAT1 |
|  | AURKA | QPCT | AMY2A | REN | BST1 | NOS2 | ME2 | ADH1C | HINT1 | OAT |
|  | LGALS7 | CTSD | IGLV2-8 | REG1A | LGALS2 | MMP12 | PARP1 | GALK1 | GSTA3 | GSTM2 |
|  | EGFR | GSR | MIF | DUSP6 | ISG20 | PCK1 | LGALS3 | FGFR2 | ARHGAP1 | SRM |
|  | CA12 | NOS3 | PDPK1 | FHIT | NR3C2 | PLA2G2A | RHEB | C1S | JAK2 | CLEC4M |
|  | ESR1 | MAPK8 | KDR | MMP13 | PTPN1 | CFD | SDS | CLK1 | ERBB4 | S100A9 |
|  | ALB | PYGL | APCS | ADH1B | MAN1B1 | PIK3CG | SHMT1 | TRAPPC3 | APRT | FABP3 |
|  | METAP1 | PAH | DHFR | MMP3 | RAB11A | CTNNA1 | GNPDA1 | ALDOA | TPH1 | PLEKHA4 |
|  | STS | HK1 | AMD1 | MMP8 | IGF1R | YARS1 | MAPKAPK2 | ACP3 | GP1BA | PPCDC |
|  | MAOB | BCAT2 | CSNK1G2 | PDE3B | JAK3 | DCK | THRA | PADI4 | HNF4G |  |
| Calycosin | ESR1 | RXRA | PDE4B | MMP12 | RHOA | ARHGAP1 | PDK2 | PAPSS1 | PPARA | SELE |
|  | MMP3 | SHBG | HSD11B1 | HPGDS | TNK2 | GALE | HEXB | HINT1 | RARG | GNPDA1 |
|  | FAP | ANG | SRC | CYP2C9 | MET | ACP3 | SULT2B1 | BLVRB | BIRC7 | WARS1 |
|  | ESR2 | HSP90AA1 | AKT1 | AKR1C3 | NR1H3 | CD1A | JAK2 | B3GAT1 | F11 | NDST1 |
|  | GBA | DUSP6 | FGFR1 | GSK3B | CASP3 | GSTA1 | TK1 | FECH | TEK | PNMT |
|  | PIM1 | F2 | GC | PTPN11 | SEC14L2 | BST1 | HAGH | NT5M | GSTO1 | DAPK1 |
|  | BACE1 | MIF | PDPK1 | NR1H4 | JAK3 | BHMT | CLK1 | VDR | DDX39B | NMNAT3 |
|  | CA2 | CDK6 | TYMS | ITK | NR1H2 | CBS | BRAF | CDK7 | TPSB2 | CTSB |
|  | AR | NQO1 | FNTA | METAP2 | CRABP2 | ALDOA | NR3C1 | TGFB2 | GSTM2 | MAPK12 |
|  | CFB | LTA4H | SORD | DHODH | ABO | SULT1E1 | PRKCQ | GP1BA | HADH | ARL5A |
|  | MAPK8 | AMY1A | AHCY | NQO2 | IMPA1 | HPRT1 | IL2 | GCDH | PPARG | RAB9A |
|  | CYP19A1 | HSPA8 | PCK1 | LCK | HCK | GCK | CHIT1 | ARG1 | ADAM33 | ATIC |
|  | CES1 | KDR | ADH5 | ABO | PDE5A | CCNT1 | LGALS3 | OAT | PIK3R1 | HSP90AB1 |
|  | MAPK10 | MMP8 | AZGP1 | SERPINA1 | IGF1 | GSTT2B | THRB | HRAS | CDC42 | MMP1 |
|  | CCNA2 | ESRRG | DCK | NMNAT1 | UMPS | KIT | PTK2 | RAB5A | GSTA3 | GLO1 |
|  | PGR | CHEK1 | PLAU | SDS | KYAT1 | RAB11A | XIAP | FABP7 | LCN2 | FOLH1 |
|  | CDK2 | PKIA | DHFR | IGF1R | NR1I2 | FABP4 | MAN1B1 | ABL1 | GSTM1 | EEA1 |
|  | TTR | AKR1B1 | PTPN1 | SHMT1 | TPI1 | PARP1 | MMP9 | RBP4 | KAT2B | RAP2A |
|  | ADAM17 | MAPK14 | NR3C2 | REG1A | TGM3 | MAOB | EPHA2 | GART | CTSF | MAP2K1 |
|  | ALB | DPP4 | CTSK | FKBP1A | RNASE3 | FABP6 | TPH1 | MTHFD1 | ARF4 | RARB |
|  | STS | SULT2A1 | ISG20 | IMPDH2 | TGFBR1 | PRKACA | MMP2 | RAF1 | CMA1 | GMPR |
|  | AURKA | PYGL | ADH1C | ELANE | THRA | MME | PPCDC | RARA | AMY2A | GRB2 |
|  | APOA2 | EGFR | HK1 | UCK2 | FHIT | DTYMK | NOS2 | PKLR | SETD7 | RXRB |
|  | GSR | PPARD | AKR1C1 | AMD1 | GPI | CCL5 | S100A9 | CD209 | RNASE2 | DCXR |
|  | PAH | F10 | HPN | REN | THRB | MAPKAPK2 | OTC | MAPK1 | FGG | GMPR2 |
|  | HSD17B1 | NOS3 | ALDH2 | HDAC8 | GSTP1 | PLA2G2A | FABP3 | DPEP1 | PPP1CC | GSTZ1 |
|  | PDE4D | PNP | SOD2 | ADK | INSR | CTSS | SRM | HEXB | GLTP | RORA |
|  | EPHB4 | CBR1 | CTNNA1 | SYK | LYZ | ACADM | CDK5R1 | EIF4E | MMP16 | ITPKA |
|  | MTAP | KIF11 | HMGCR | RHEB | TRAPPC3 | ZAP70 | AGXT | BCAT2 | ERI1 | DUT |
|  | MMP13 | HSP90AA1 | LSS | ERBB4 | HSPA1A | F7 | HNF4G | CLEC4M | FKBP1B |  |
| Jatrorrhizine | ESR1 | NOS3 | PTPN1 | PDK2 | LSS | ACADM | TPH1 | RXRB | NDST1 | TAP1 |
|  | TTR | SHBG | GC | BACE1 | FKBP1A | TEK | CTSF | UCK2 | GSTM2 | RAC1 |
|  | AR | PDE4D | DUSP6 | CDK6 | CBR1 | DCK | ABL1 | STAT1 | OAT | MMP1 |
|  | CA2 | CCNA2 | MMP12 | ALDH2 | ACP3 | F11 | CYP2C8 | CTSB | RAB11A | DCPS |
|  | GSTP1 | GSR | AKR1B1 | NR1H3 | THRB | NR3C1 | HMGCR | FABP7 | CDK7 | HADH |
|  | ESR2 | PAH | AKR1C3 | EPHX2 | FABP3 | TGFBR1 | RARB | DTYMK | PTK2 | WARS1 |
|  | QPCT | SRC | HSPA8 | ITK | MAPK8 | RARA | PPP1CC | INSR | RAF1 | APAF1 |
|  | AKR1C2 | PKIA | PDE4B | HPGDS | PPARG | FABP6 | FECH | FKBP1B | RORA | RFK |
|  | STS | AKT1 | PDE3B | MMP7 | MTHFD1 | SDS | FDPS | LYZ | HNMT | PNMT |
|  | APOA2 | PGR | PDPK1 | JAK3 | B3GAT1 | IGF1R | RARG | CASP3 | TRDMT1 | ARL5B |
|  | PPIA | ALB | PTPN11 | MTAP | ERBB4 | MMP9 | CCL5 | CMA1 | SELE | TGFB2 |
|  | CDK2 | PDPK1 | RBP4 | LCK | GBA | ADAM17 | MMP16 | GSTM1 | BCL2L1 | IVD |
|  | BCHE | HSD17B1 | PPP5C | ADK | MAPKAPK2 | PAPSS1 | CTSS | HPRT1 | RNASE2 | RAP2A |
|  | BMP2 | MMP13 | CASP7 | CSNK1G2 | IGF1 | FABP4 | VDR | HMOX1 | GLTP | GMPR2 |
|  | NR1H2 | F2 | HDAC8 | DHODH | SYK | PPCDC | MMP2 | NT5M | EIF4E | PCK1 |
|  | RXRA | SULT2A1 | TTPA | NMNAT1 | GSTA1 | HCK | RHOA | GCK | CASP1 | KIT |
|  | MAPK14 | SORD | MMP3 | METAP2 | ZAP70 | CRABP2 | BST1 | GPI | RND3 | UAP1 |
|  | CYP19A1 | ANXA5 | SOD2 | DHFR | THRA | CTSG | HSPA1A | HINT1 | ARL5A | PROCR |
|  | CFD | MET | ADH5 | NR1I2 | SULT1E1 | S100A9 | JAK2 | HSP90AB1 | SEC14L2 | GSTT2B |
|  | EPHB4 | AKR1C1 | XIAP | NQO1 | REN | ARHGAP1 | SETD7 | CDC42 | DUT | FKBP3 |
|  | KDR | HSD11B1 | PDE5A | FGFR1 | ABO | TRAPPC3 | PPARA | RAB5A | BCAT2 | DOT1L |
|  | BRAF | ADH1C | CTNNA1 | AURKA | CTSK | GSTA3 | CBS | AMD1 | ADAM33 | PFKFB1 |
|  | ADAM17 | EGFR | F10 | PGF | PRKCQ | DPEP1 | OTC | CYP2C9 | GMPR | GART |
|  | NQO2 | MMP8 | KIF11 | F7 | PLA2G2A | SERPINA1 | MAP2K1 | AMY2A | DAPK1 | SIRT5 |
|  | ESRRG | TYMS | GSK3B | BHMT | HNF4G | TGM3 | LCN2 | HEXB | HRAS | SPR |
|  | PIM1 | HSP90AA1 | MAPK10 | MAOB | MME | SULT2B1 | BIRC7 | TPSB2 | FOLH1 | AK1 |
|  | CHEK1 | NR3C2 | DPP4 | NR1H4 | IL2 | ELANE | LTA4H | KAT2B | FGG | RAN |
| Palmatine | PPIA | NOS3 | TYMS | MMP3 | PGF | HNF4G | SERPINA1 | CCL5 | SELE | RAC1 |
|  | TTR | GSR | HSP90AA1 | CDK6 | DHODH | PRKCQ | PTK2 | SETD7 | HMOX1 | F11 |
|  | AR | PKIA | EPHX2 | BACE1 | METAP2 | PLA2G2A | NR1H4 | CBS | HPRT1 | DCPS |
|  | CA2 | CCNA2 | PPP5C | SOD2 | HDAC8 | ABO | ELANE | BCAT2 | GSTM2 | TAP1 |
|  | CYP19A1 | SRC | PTPN1 | GSK3B | BHMT | ADAM17 | TGM3 | OTC | RORA | CASP3 |
|  | GSTP1 | PDPK1 | GC | THRB | AURKA | SULT1E1 | DHFR | CTSS | CTSB | APAF1 |
|  | ESR1 | PGR | AKR1C3 | MAPK10 | MTHFD1 | IGF1R | HCK | STAT1 | OAT | GMPR2 |
|  | AKR1C2 | HSD17B1 | MMP12 | PDE5A | PPARG | MME | TPH1 | LTA4H | ABL1 | PNMT |
|  | STS | ALB | KDR | MAOB | FGFR1 | IL2 | THRA | INSR | CDK7 | ARL5B |
|  | APOA2 | ANXA5 | HSPA8 | NR1H3 | FKBP1A | RARA | DTYMK | MAP2K1 | TRDMT1 | SEC14L2 |
|  | BCHE | F2 | CASP7 | HPGDS | ERBB4 | NR3C1 | FECH | LCN2 | CHIT1 | TGFB2 |
|  | BMP2 | SULT2A1 | PDE4B | MAPK8 | TGFBR1 | TRAPPC3 | NMNAT1 | BIRC7 | ARL5A | DDX39B |
|  | NR1H2 | CDK2 | PDE3B | PDK2 | FABP3 | PARP1 | RARG | FABP7 | ADAM33 | PCK1 |
|  | ADAM17 | F10 | ADH5 | REG1A | ACP3 | PAPSS1 | CYP2C8 | RXRB | CASP1 | HRAS |
|  | RXRA | MET | PDPK1 | LCK | IGF1 | FABP6 | FDPS | NT5M | DUT | UAP1 |
|  | MAPK14 | SORD | MMP8 | ITK | MAPKAPK2 | TEK | RARB | CMA1 | RAB5A | PROCR |
|  | CFD | CHEK1 | PTPN11 | EPHB4 | RBP4 | CTSK | PPARA | GPI | DOT1L | GART |
|  | NQO2 | HSD11B1 | IGLV2-8 | DPP4 | CTSG | SDS | PPP1CC | KAT2B | HNMT | DAPK1 |
|  | PIM1 | NR3C2 | TTPA | MTAP | REN | ZAP70 | VDR | FKBP1B | FGG | RAP2A |
|  | BRAF | ESR2 | AKR1B1 | JAK3 | GSTA1 | CRABP2 | BST1 | GSTM1 | TPSB2 | SPR |
|  | MMP13 | ADH1C | XIAP | CDA | S100A9 | HMGCR | CYP2C9 | EIF4E | HADH | AK1 |
|  | ESRRG | AKR1C1 | KIF11 | NQO1 | ACADM | GSTA3 | MMP2 | HINT1 | RFK | SIRT5 |
|  | SHBG | EGFR | PAH | ADK | SULT2B1 | DPEP1 | CTSF | AMY2A | GMPR | FKBP3 |
|  | PDE4D | DUSP6 | CTNNA1 | NR1I2 | FABP4 | DCK | PYGL | UCK2 |  |  |
| Licoisoflavanone | BACE1 | MAPKAPK2 | CTSB | ADH1B | RBP4 | HPN | CCNT1 | HEXB | RAB5A | ARHGAP1 |
|  | MAPK1 | MAPK8 | ANG | FNTA | AKR1C3 | PPP5C | PCK1 | ITGAL | GLO1 | RAC2 |
|  | AR | MMP3 | PDE4D | SHBG | RXRA | FGFR2 | FKBP1A | TGM3 | MMP2 | GPI |
|  | CA2 | PNP | TYMS | MMP13 | SEC14L2 | HK1 | CYP2C9 | NOS2 | PRKACA | ST14 |
|  | THRB | CES1 | PPARG | PPARD | CALM1 | AMD1 | LCK | SULT1E1 | THRA | MMP9 |
|  | HCK | F2 | DCK | ESR2 | UCK2 | PAK5 | SULT2B1 | KYAT1 | PITPNA | C1S |
|  | CFB | GSR | SRC | DHODH | NR1H4 | ADH5 | HNF4G | ALDH2 | ACP3 | SETD7 |
|  | EGFR | TTR | BCAT2 | IGLV2-8 | NQO2 | PADI4 | ADK | RARA | JAK2 | AGXT |
|  | CASP3 | CLPP | NQO1 | CFD | YARS1 | DAPK1 | TTPA | RARG | ACADM | LYZ |
|  | STS | PIK3CG | FGFR1 | LGALS7 | HDAC8 | BST1 | CBS | PRKCQ | ACE | EPHA2 |
|  | TREM1 | CDK5R1 | HSPA8 | CTSK | CTNNA1 | XIAP | ERBB4 | RHOA | S100A9 | GART |
|  | MAPK14 | AKR1C2 | DHFR | CDK6 | PLAU | MDM2 | AKT2 | BIRC7 | DTYMK | HRAS |
|  | PLK1 | CCNA2 | MTAP | PDE3B | LSS | AMY1A | DPEP1 | RAB11A | LCN2 | APRT |
|  | ALB | BMP2 | DUSP6 | ICAM2 | WAS | FABP6 | IGF1 | PLA2G2A | HEXB | MAN1B1 |
|  | KIF11 | LTA4H | PTPN1 | TNNC1 | SORD | SHMT1 | ABO | UMPS | KIT | HAGH |
|  | APOA2 | PDE4B | HSD11B1 | HPGDS | REN | AURKA | PPARA | ZAP70 | GSTT2B | CD1A |
|  | GSTP1 | ADAM17 | ANXA5 | CSNK2A1 | SOD2 | FABP5 | GSTA1 | TGFB2 | CTSF | RARB |
|  | MAPK10 | EPHB4 | PARP1 | DPP4 | NR1I2 | REG1A | F7 | FABP3 | LGALS3 | ALAD |
|  | KDR | F10 | HSD17B1 | ELANE | CBR1 | JAK3 | GM2A | RNASE4 | VDR | TPH1 |
|  | BCHE | HSD17B11 | PLA2G10 | AKR1C1 | ADH1C | IL2 | PDK2 | THRB | HNMT | PPP1CC |
|  | ESRRG | PPIA | NR3C2 | IGF1R | ISG20 | BLVRB | MTHFD1 | PGF | CLK1 | HSP90AB1 |
|  | MAOB | CHEK1 | HSP90AA1 | PKIA | HMGCR | NR1I3 | FECH | MME | CYP2C8 | ARG1 |
|  | CASP7 | CTSS | TYMP | MMP8 | MMP12 | SDS | LGALS2 | AMY1A | IMPDH1 | RAB9A |
|  | PIM1 | SULT2A1 | NOS3 | BRAF | AHCY | ABL1 | FABP7 | TPI1 | SRM | GSTM1 |
|  | CDK2 | PGR | AKT1 | NR1H2 | ITK | ATOX1 | CSK | ESRRA | PLEKHA4 | BHMT |
|  | AKR1B1 | PDE5A | PYGL | MIF | CDA | MMP7 | B3GAT1 | ALDOA | CHIT1 | CDC42 |
|  | GBA | TGFBR1 | RORA | PTPN11 | PAH | ABO | NR3C1 | FHIT | NMNAT1 | FKBP1B |
|  | MAPK14 | PNMT | NR1H3 | GSK3B | EPHX2 | TRAPPC3 | RNASE3 | GCK | PSAP | OTC |
|  | CA1 | GC | PDPK1 | SERPINA1 | MET | TNK2 | FABP4 | HSPA1A | CRABP2 | EIF4E |
|  | ESR1 | METAP2 | CYP19A1 | AZGP1 | SYK | AMY2A | MAP2K1 | CCL5 | PPCDC |  |
| Berberine | ESR1 | SRC | AKR1C1 | CTNNA1 | METAP2 | ABO | CRABP2 | ZAP70 | HINT1 | DCPS |
|  | TTR | PKIA | PPP5C | SOD2 | GSTA1 | THRA | GSTP1 | LCN2 | CTSF | APAF1 |
|  | PPIA | CCNA2 | CDK6 | KIF11 | MTAP | PTK2 | RARA | CYP2C9 | FKBP1B | GMPR |
|  | CA2 | MAPK14 | DUSP6 | THRB | LCK | GSTA3 | TGM3 | CTSS | HPRT1 | RFK |
|  | AKR1C2 | PGR | PTPN1 | MMP8 | NR1H4 | IGF1R | PYGL | INSR | TPSB2 | PNMT |
|  | CYP19A1 | PDPK1 | GC | MAPK10 | MAOB | TRAPPC3 | DHFR | ELANE | RORA | HRAS |
|  | AR | ALB | EGFR | BACE1 | FKBP1A | DCK | MAP2K1 | STAT1 | GPI | RAP2A |
|  | STS | F2 | PDE3B | HDAC8 | NR1I2 | ADAM17 | TPH1 | LTA4H | HNMT | TAP1 |
|  | APOA2 | ANXA5 | PDPK1 | PDK2 | IGF1 | NR3C1 | FECH | HMGCR | FOLH1 | DOT1L |
|  | BMP2 | MTHFD1 | CASP7 | HPGDS | PPARG | S100A9 | RBP4 | EIF4E | OAT | SEC14L2 |
|  | RXRA | HSD17B1 | CHEK1 | AKR1C3 | BHMT | FABP6 | FDPS | CTSK | SELE | PPARD |
|  | NR1H2 | SORD | PDE4B | MMP3 | FGFR1 | HCK | CBS | GSTM1 | ARL5A | DAPK1 |
|  | PIM1 | AKR1B1 | F10 | NR1H3 | JAK3 | TEK | RARB | PRKCQ | CASP1 | PCK1 |
|  | BCHE | HSD11B1 | HSPA8 | GSK3B | ERBB4 | TGFBR1 | SETD7 | RXRB | TRDMT1 | CTSB |
|  | ADAM17 | NCOA5 | PTPN11 | PDE5A | SULT2B1 | NMNAT1 | PPP1CC | HMOX1 | DUT | PROCR |
|  | CFD | CDK2 | NQO2 | EPHX2 | REN | SDS | VDR | BIRC7 | BCAT2 | ARL5B |
|  | ESRRG | SULT2A1 | ADH5 | DPP4 | SULT1E1 | FABP4 | CYP2C8 | NT5M | RAB5A | PFKFB1 |
|  | BRAF | NR3C2 | TTPA | NQO1 | CTSG | RARG | MMP2 | CMA1 | FGG | SIRT5 |
|  | MMP13 | TYMS | MET | PGF | ACADM | DPEP1 | CCL5 | UCK2 | ADAM33 | SPR |
|  | PDE4D | HSP90AA1 | XIAP | ADK | PLA2G2A | IL2 | DTYMK | OTC | HADH | AMY2A |
|  | NOS3 | MMP12 | PAH | DHODH | FABP3 | SERPINA1 | ABL1 | KAT2B | RAC1 | GSTT2B |
|  | GSR | KDR | ITK | MAPKAPK2 | MME | PAPSS1 | FABP7 | GSTM2 | CASP3 | FKBP3 |
|  | SHBG | ADH1C | EPHB4 | AURKA | HNF4G |  |  |  |  |  |
| Pachymic acid | GC | PGR | DPP4 | ADH1B | GSR | ABO | AMD1 | CTSG | LTA4H | AGXT |
|  | THRB | MAOB | PPARG | CALM1 | PPARA | ME2 | KYAT1 | FECH | HEXB | F11 |
|  | ALB | CCNA2 | PPP5C | PLA2G10 | HMGCR | FABP6 | HNF4G | BIRC7 | ST14 | KIT |
|  | PIM1 | SEC14L2 | SRC | CSNK2A1 | PLK1 | IGF1 | PADI4 | FKBP1A | GRB2 | LGALS3 |
|  | BMP2 | BCHE | EGFR | CFD | ESRRA | PGF | IL2 | CYP2C9 | S100A9 | PPCDC |
|  | AKR1C2 | CA1 | PDE4D | WAS | MET | MMP12 | GLO1 | JAK2 | CBS | TGFB2 |
|  | APOA2 | MAPK1 | PTPN1 | MTHFD1 | AKR1C1 | PCK1 | PLAU | DPEP1 | RXRB | ARSA |
|  | STS | MMP13 | DHFR | PRKACA | MMP8 | FABP5 | FABP7 | PRKACA | PIK3R1 | HEXB |
|  | CA2 | KIF11 | SORD | DHODH | TTPA | PDE3B | RARG | CDK6 | ISG20 | GCK |
|  | HSD17B11 | EPHB4 | PDE4B | ESRRG | CHIT1 | REN | THRA | ABO | LDHB | HSPA1A |
|  | MAPK10 | CDK5R1 | FNTA | HDAC8 | BLVRB | F7 | ALDH2 | MMP7 | TNK2 | HAGH |
|  | TTR | AR | METAP2 | LCK | ITK | LGALS7 | NR1I3 | RARB | MME | OAT |
|  | GSTP1 | ANXA5 | SOD2 | RXRA | GSTA1 | PAK5 | FGF1 | GALE | PCTP | ACAT1 |
|  | NR1H2 | HSD11B1 | TYMS | PRDX5 | MAP2K1 | SULT2B1 | CPB1 | AMY1A | SETD7 | MMP16 |
|  | MAPK8 | AKR1B1 | PDPK1 | KIF5B | IGF1R | PIK3CG | ANG | ACP3 | PPP1CC | GSTM1 |
|  | PPIA | MAPK14 | BRAF | CSNK1G2 | RBP4 | FGFR1 | ZAP70 | RARA | LYZ | GCDH |
|  | KDR | TGFBR1 | ADH5 | ARF1 | NR1H3 | PDE5A | OTC | UCK2 | BST1 | STAT1 |
|  | CLPP | MAPK14 | QPCT | TREM1 | JAK3 | CBR1 | HCK | THRB | PYGL | HMOX1 |
|  | CASP3 | CDK2 | LCN2 | TRAPPC3 | PLA2G2A | CCNT1 | APCS | NR1I2 | MMP9 | RNASE3 |
|  | ESR2 | NR3C2 | AURKA | PARP1 | FGFR2 | CDA | HNMT | HPN | PAPSS1 | GPI |
|  | CFB | CMA1 | HSPA8 | MIF | SERPINA1 | PAH | GM2A | FDPS | HSP90AB1 | EIF4E |
|  | F2 | ESR1 | PNMT | BCAT2 | MTAP | YARS1 | ERBB4 | PSAP | SHMT1 | CLK1 |
|  | CHEK1 | CTSS | ADAM17 | NQO1 | ELANE | CSK | CRABP2 | CTSF | SDS | PROCR |
|  | MMP3 | SULT2A1 | NQO2 | IGLV2-8 | FABP4 | CTNNA1 | DCK | TGM3 | CD1A | FKBP1B |
|  | MAPKAPK2 | HSD17B1 | CES1 | ADK | SYK | MMP2 | NMNAT1 | BHMT | DTYMK | WARS1 |
|  | RORA | F10 | SHBG | CYP2C8 | CTSB | XIAP | VDR | ALDOA | TEK | GSTM2 |
|  | HSP90AA1 | NOS3 | PTPN11 | HPGDS | SULT1E1 | GATM | NR3C1 | CASP1 | TPH1 | NOS2 |
|  | BACE1 | NR1H4 | PPARD | PDK2 | LSS | EPHX2 | FHIT | TPSB2 | GSTT2B | AMY2A |
|  | ITGAL | TNNC1 | DAPK1 | ADH1C | DUSP6 | FABP3 | PRKCQ | ACADM | CDK7 | MAPK12 |
|  | CASP7 | GSK3B | AKR1C3 | MDM2 | C1R | CTSK | AKT2 | ABL1 | BCL2L1 |  |

**Supplementary Table 2. DEGs of FSGS.**

| **Gene ID** | **adj P-Value** | **logFC** | **SPOT ID** | **Regulation** |
| --- | --- | --- | --- | --- |
| TC17000132.hg.1 | 1.45E-10 | 2.17699 | chr17(+):8090263-8090322 | Up |
| TC06001447.hg.1 | 1.49E-10 | 1.89313 | chr6(-):28565117-28565198 | Up |
| TC15001239.hg.1 | 1.49E-10 | 1.89313 | chr15(-):40886023-40886104 | Up |
| TC06000239.hg.1 | 4.4E-10 | 1.671739 | chr6(+):27265775-27265856 | Up |
| TC06001356.hg.1 | 4.4E-10 | 1.650548 | chr6(-):26305718-26305801 | Up |
| TC14000061.hg.1 | 4.4E-10 | 1.212129 | chr14(+):21093529-21093610 | Up |
| TC06000222.hg.1 | 6.56E-10 | 1.665125 | chr6(+):27065085-27065166 | Up |
| TC17001102.hg.1 | 1.4E-09 | 1.053523 | chr17(-):8023632-8023713 | Up |
| TC11000663.hg.1 | 1.59E-09 | 1.589823 | chr11(+):66115591-66115672 | Up |
| TC12001104.hg.1 | 1.59E-09 | 1.585917 | chr12(-):2934512-2944221 | Up |
| TC06000278.hg.1 | 4.37E-09 | 1.665674 | chr6(+):28180815-28180896 | Up |
| TC02001640.hg.1 | 9.48E-09 | 2.602422 | chr2(-):23729913-23747214 | Up |
| TC02002325.hg.1 | 1.13E-08 | 2.291811 | chr2(-):131031334-131031399 | Up |
| TC02000848.hg.1 | 2.13E-08 | 1.275758 | chr2(+):132141814-132141886 | Up |
| 3686582_st | 4.07E-08 | 2.05373 | normgene->exon | Up |
| TC21000345.hg.1 | 5.88E-08 | 2.054699 | chr21(-):28208606-28217728 | Up |
| TC05000736.hg.1 | 6.21E-08 | 2.314614 | chr5(+):140105743-140105831 | Up |
| TC01002064.hg.1 | 6.52E-08 | 3.245755 | chr1(-):566187-566265 | Up |
| PSR17024061.hg.1 | 7.07E-08 | 1.5943 | 19503239 | Up |
| TC02004114.hg.1 | 0.000000106 | 2.20836 | chr2(-):23724795-23727929 | Up |
| TC17000120.hg.1 | 0.000000176 | 1.271489 | chr17(+):7788123-7816078 | Up |
| TC07000373.hg.1 | 0.000000176 | 1.291549 | chr7(+):63570705-63570776 | Up |
| PSR11017847.hg.1 | 0.00000021 | 1.03571 | 19376933 | Up |
| TC17001271.hg.1 | 0.000000342 | 1.564518 | chr17(-):22026512-22026577 | Up |
| TC11000459.hg.1 | 0.000000446 | 2.923783 | chr11(+):56949221-56959191 | Up |
| TC16000947.hg.1 | 0.000000589 | 1.85547 | chr16(-):22207032-22207113 | Up |
| TC17000843.hg.1 | 0.000000711 | 1.038792 | chr17(+):73031208-73031280 | Up |
| TC12001803.hg.1 | 0.00000074 | 1.409417 | chr12(-):91496406-91505608 | Up |
| TC16000232.hg.1 | 0.00000074 | 1.41386 | chr16(+):21414221-21414250 | Up |
| TC16000233.hg.1 | 0.00000074 | 1.41386 | chr16(+):21414990-21415019 | Up |
| TC16000318.hg.1 | 0.00000074 | 1.41386 | chr16(+):29393170-29393199 | Up |
| TC16000950.hg.1 | 0.00000074 | 1.41386 | chr16(-):22545696-22545725 | Up |
| TC16000951.hg.1 | 0.00000074 | 1.41386 | chr16(-):22545822-22545851 | Up |
| TC16000952.hg.1 | 0.00000074 | 1.41386 | chr16(-):22545936-22545965 | Up |
| TC16000953.hg.1 | 0.00000074 | 1.41386 | chr16(-):22546292-22546321 | Up |
| TC16000954.hg.1 | 0.00000074 | 1.41386 | chr16(-):22546544-22546573 | Up |
| TC16000955.hg.1 | 0.00000074 | 1.41386 | chr16(-):22546796-22546825 | Up |
| TC16000956.hg.1 | 0.00000074 | 1.41386 | chr16(-):22546935-22546964 | Up |
| TC16000957.hg.1 | 0.00000074 | 1.41386 | chr16(-):22547061-22547090 | Up |
| TC06001800.hg.1 | 0.00000074 | 1.364505 | chr6(-):52362200-52441862 | Up |
| TC03002693.hg.1 | 0.00000149 | 1.20537 | chr3(+):184033948-184038499 | Up |
| TC17001269.hg.1 | 0.00000153 | 1.543404 | chr17(-):22026337-22026405 | Up |
| TC14002154.hg.1 | 0.00000166 | 1.420934 | chr14(-):99966517-99968335 | Up |
| TC04001473.hg.1 | 0.00000169 | 1.187762 | chr4(-):111781738-111781803 | Up |
| TC21000747.hg.1 | 0.00000179 | 1.131176 | chr21(+):43719104-43720919 | Up |
| TC17001990.hg.1 | 0.00000179 | 2.070379 | chr17(+):56209-56601 | Up |
| TC16000323.hg.1 | 0.00000182 | 1.09203 | chr16(+):29495626-29495781 | Up |
| TC09002059.hg.1 | 0.00000197 | 3.96013 | chr9(+):89366554-89370049 | Up |
| TC11001415.hg.1 | 0.00000235 | 2.238078 | chr11(-):10578513-10633236 | Up |
| TC05000187.hg.1 | 0.00000263 | 2.083759 | chr5(+):40909354-40983042 | Up |
| TC11002676.hg.1 | 0.0000029 | 2.601192 | chr11(+):56956461-56959187 | Up |
| TC0X001843.hg.1 | 0.00000321 | 1.099342 | chrX(+):114957297-115021470 | Up |
| TC17002860.hg.1 | 0.00000325 | 1.064962 | chr17(+):66031848-66042970 | Up |
| TC10000398.hg.1 | 0.00000367 | 1.039369 | chr10(+):69524261-69524407 | Up |
| TC01000286.hg.1 | 0.00000372 | 1.130196 | chr1(+):22970118-22974603 | Up |
| TC03002372.hg.1 | 0.0000058 | 1.31004 | chr3(+):64563476-64563975 | Up |
| TC19000576.hg.1 | 0.00000621 | 1.43812 | chr19(+):41725108-41767671 | Up |
| TC05000672.hg.1 | 0.00000728 | 1.100755 | chr5(+):134259743-134259812 | Up |
| TC20000913.hg.1 | 0.00000763 | 1.250075 | chr20(-):46285092-46415360 | Up |
| PSR06002932.hg.1 | 0.00000778 | 1.027262 | 19214337 | Up |
| TC02004115.hg.1 | 0.00000784 | 2.700068 | chr2(-):23839388-23840857 | Up |
| TC02001641.hg.1 | 0.00000849 | 2.599021 | chr2(-):23839369-23840857 | Up |
| TC12000668.hg.1 | 0.00000864 | 1.134916 | chr12(+):80838126-81073968 | Up |
| TC11002097.hg.1 | 0.00000903 | 1.002995 | chr11(-):75282944-75283832 | Up |
| TC12002478.hg.1 | 0.00000907 | 1.575147 | chr12(+):81062891-81067044 | Up |
| TC10001535.hg.1 | 0.00000914 | 1.075259 | chr10(-):95066186-95242074 | Up |
| TC12002477.hg.1 | 0.0000096 | 1.152979 | chr12(+):80818224-80819003 | Up |
| TC14001443.hg.1 | 0.0000113 | 1.270866 | chr14(-):92335755-92414167 | Up |
| TC02002747.hg.1 | 0.0000122 | 1.602128 | chr2(-):216225163-216300895 | Up |
| TC12002476.hg.1 | 0.0000137 | 1.29157 | chr12(+):80799677-80815204 | Up |
| TC06001652.hg.1 | 0.0000148 | 1.296925 | chr6(-):34212964-34214008 | Up |
| 3597948_st | 0.0000167 | 1.297245 | normgene->exon | Up |
| TC10000727.hg.1 | 0.000017 | 1.206795 | chr10(+):102729275-102745628 | Up |
| TC19002460.hg.1 | 0.0000187 | 1.175341 | chr19(-):36245427-36246208 | Up |
| TC01003166.hg.1 | 0.000021 | 1.346968 | chr1(-):149294666-149294736 | Up |
| TC03002108.hg.1 | 0.0000233 | 1.206975 | chr3(-):190023490-190040264 | Up |
| TC13000872.hg.1 | 0.0000273 | 1.228107 | chr13(-):110801310-110959496 | Up |
| 3686581_st | 0.0000287 | 1.08207 | normgene->exon | Up |
| TC01003717.hg.1 | 0.0000296 | 1.606841 | chr1(-):203136939-203144942 | Up |
| TC16000461.hg.1 | 0.0000365 | 1.043539 | chr16(+):56225251-56391356 | Up |
| TC22000301.hg.1 | 0.0000407 | 1.602876 | chr22(+):38864067-38879452 | Up |
| TC22000010.hg.1 | 0.0000433 | 1.030711 | chr22(+):16564242-16564323 | Up |
| TC11003400.hg.1 | 0.0000437 | 1.772222 | chr11(-):125827345-125830001 | Up |
| TC0X000450.hg.1 | 0.0000439 | 1.375545 | chrX(+):89045334-89045450 | Up |
| TC15002743.hg.1 | 0.0000547 | 1.006774 | chr15(-):101087837-101099853 | Up |
| TC16001960.hg.1 | 0.0000634 | 1.049475 | chr16(-):74411388-74411717 | Up |
| TC07000965.hg.1 | 0.0000664 | 1.173225 | chr7(+):147626685-148043927 | Up |
| TC05000132.hg.1 | 0.000069 | 1.389363 | chr5(+):31193762-31329253 | Up |
| TC01006051.hg.1 | 0.0000716 | 2.598349 | chr1(-):204131143-204141704 | Up |
| TC01004845.hg.1 | 0.0000729 | 1.427739 | chr1(+):176432304-176525140 | Up |
| TC16001574.hg.1 | 0.0000762 | 1.574331 | chr16(+):56374758-56377888 | Up |
| TC01004129.hg.1 | 0.0000929 | 1.702135 | chr1(+):1317609-1318166 | Up |
| TC01003453.hg.1 | 0.0000942 | 1.653405 | chr1(-):162286642-162287584 | Up |
| TC19001925.hg.1 | 0.0000951 | 2.010948 | chr19(+):302198-304460 | Up |
| TC14000623.hg.1 | 0.00011 | 1.280104 | chr14(+):99978838-99979913 | Up |
| TC09001605.hg.1 | 0.00012 | 1.208857 | chr9(-):130452966-130453074 | Up |
| TC01002099.hg.1 | 0.000124 | 1.300061 | chr1(-):1363502-1365635 | Up |
| TC05001884.hg.1 | 0.000141 | 1.515871 | chr5(-):142005734-142005811 | Up |
| TC17000965.hg.1 | 0.000142 | 1.039026 | chr17(-):289769-295731 | Up |
| TC06001861.hg.1 | 0.000143 | 1.073015 | chr6(-):72086663-72086734 | Up |
| TC20000783.hg.1 | 0.000143 | 1.083932 | chr20(-):33114315-33114411 | Up |
| TC01005197.hg.1 | 0.000144 | 1.19378 | chr1(-):757786-759075 | Up |
| TC01004132.hg.1 | 0.000146 | 1.317914 | chr1(+):1363502-1365634 | Up |
| TC01003726.hg.1 | 0.00016 | 3.93157 | chr1(-):204123944-204135465 | Up |
| TC06003992.hg.1 | 0.000163 | 1.061427 | chr6(-):166253092-166253578 | Up |
| TC01001188.hg.1 | 0.000178 | 1.919955 | chr1(+):150480487-150486265 | Up |
| TC06002799.hg.1 | 0.000201 | 1.010472 | chr6(+):43737962-43739182 | Up |
| TC09000168.hg.1 | 0.000201 | 1.11659 | chr9(+):34133756-34133782 | Up |
| TC0X000187.hg.1 | 0.000201 | 1.11659 | chrX(+):40219040-40219066 | Up |
| TC05001977.hg.1 | 0.000332 | 1.321682 | chr5(-):156456424-156486130 | Up |
| TC11003279.hg.1 | 0.000358 | 1.25677 | chr11(-):85416009-85420543 | Up |
| TC17002615.hg.1 | 0.000392 | 2.380464 | chr17(-):38253597-38256549 | Up |
| TC11001962.hg.1 | 0.000405 | 1.00062 | chr11(-):66081958-66084515 | Up |
| TC12001770.hg.1 | 0.000428 | 1.24967 | chr12(-):80849275-80852604 | Up |
| TC01002995.hg.1 | 0.000479 | 1.249404 | chr1(-):113004392-113004455 | Up |
| TC16000276.hg.1 | 0.000496 | 1.05627 | chr16(+):25083457-25083487 | Up |
| TC02003144.hg.1 | 0.0005 | 1.012429 | chr2(+):31494729-31496423 | Up |
| TC07001401.hg.1 | 0.0005 | 1.058413 | chr7(-):55813825-55813853 | Up |
| TC01000039.hg.1 | 0.000591 | 1.160356 | chr1(+):1363502-1365634 | Up |
| TC0M000008.hg.1 | 0.000603 | 1.385382 | chrM(+):12208-12264 | Up |
| TC05002434.hg.1 | 0.000616 | 1.105555 | chr5(+):71403313-71491128 | Up |
| 3597914_st | 0.00064 | 1.131585 | normgene->exon | Up |
| TC05002180.hg.1 | 0.000722 | 1.084171 | chr5(+):1043539-1047475 | Up |
| TC09001045.hg.1 | 0.000732 | 1.084707 | chr9(-):34709002-34710147 | Up |
| TC20000265.hg.1 | 0.000771 | 1.190537 | chr20(+):34708884-34708994 | Up |
| TC15001679.hg.1 | 0.000806 | 1.07713 | chr15(-):76084070-76084100 | Up |
| 2909175_st | 0.000806 | 1.043706 | normgene->exon | Up |
| TC15000945.hg.1 | 0.000813 | 1.16702 | chr15(+):98462784-98517068 | Up |
| TC20000067.hg.1 | 0.000924 | 1.144918 | chr20(+):6748311-6760910 | Up |
| TC10001636.hg.1 | 0.000941 | 1.039557 | chr10(-):105637318-105678045 | Up |
| TC01001057.hg.1 | 0.00108 | 1.103024 | chr1(+):142656014-142656096 | Up |
| TC09001156.hg.1 | 0.00109 | 1.182378 | chr9(-):66858537-66858612 | Up |
| TC01005949.hg.1 | 0.00111 | 1.427762 | chr1(-):175126123-175161929 | Up |
| TC01003768.hg.1 | 0.00122 | 1.487348 | chr1(-):207101863-207119811 | Up |
| TC14000004.hg.1 | 0.00122 | 1.013431 | chr14(+):19253848-19253930 | Up |
| TC01005965.hg.1 | 0.0013 | 1.430337 | chr1(-):179512336-179518057 | Up |
| TC09001165.hg.1 | 0.00133 | 1.082312 | chr9(-):69001999-69002081 | Up |
| TC17001459.hg.1 | 0.00137 | 1.577867 | chr17(-):38249037-38256978 | Up |
| TC19001955.hg.1 | 0.0014 | 1.441379 | chr19(+):6680189-6720573 | Up |
| TC21000270.hg.1 | 0.00145 | 1.125896 | chr21(-):9937936-9938018 | Up |
| TC01004688.hg.1 | 0.00155 | 1.155381 | chr1(+):144166564-144167711 | Up |
| TC17002800.hg.1 | 0.00155 | 1.10504 | chr17(-):76220063-76220513 | Up |
| TC06001342.hg.1 | 0.00166 | 1.13395 | chr6(-):26031817-26032289 | Up |
| TC01000013.hg.1 | 0.00173 | 1.26541 | chr1(+):568844-568913 | Up |
| TC07000435.hg.1 | 0.00191 | 1.07395 | chr7(+):72491731-72491760 | Up |
| TC07001522.hg.1 | 0.00191 | 1.07395 | chr7(-):74917064-74917093 | Up |
| TC07001525.hg.1 | 0.00191 | 1.07395 | chr7(-):74945087-74945116 | Up |
| TC07001527.hg.1 | 0.00191 | 1.07395 | chr7(-):74973106-74973135 | Up |
| TC01003567.hg.1 | 0.002 | 1.388172 | chr1(-):179512336-179518057 | Up |
| TC10002814.hg.1 | 0.00201 | 1.128813 | chr10(-):105637328-105638836 | Up |
| TC01003546.hg.1 | 0.00207 | 1.023247 | chr1(-):175126123-175162229 | Up |
| 2909177_st | 0.00213 | 1.009895 | normgene->exon | Up |
| 47421982_st | 0.0022 | 1.172968 | normgene->intron | Up |
| 3597952_st | 0.00259 | 1.699777 | normgene->exon | Up |
| TC01003076.hg.1 | 0.0027 | 1.023461 | chr1(-):143437327-143437409 | Up |
| TC12001281.hg.1 | 0.00281 | 1.145908 | chr12(-):15499411-15501609 | Up |
| TC09001166.hg.1 | 0.00314 | 1.051999 | chr9(-):69002239-69002321 | Up |
| TC06001777.hg.1 | 0.00321 | 1.027893 | chr6(-):47199268-47277680 | Up |
| TC13000234.hg.1 | 0.00343 | 1.172184 | chr13(+):58205789-58303445 | Up |
| TC11002894.hg.1 | 0.00351 | 1.316851 | chr11(+):117073731-117075072 | Up |
| TC17001915.hg.1 | 0.00359 | 1.435188 | chr17(-):76220157-76220780 | Up |
| TC21000269.hg.1 | 0.00374 | 1.229917 | chr21(-):9937696-9937777 | Up |
| TC04002100.hg.1 | 0.0038 | 1.371475 | chr4(+):88896901-88901613 | Up |
| TC04000285.hg.1 | 0.00457 | 1.216379 | chr4(+):49200224-49200306 | Up |
| TC04000478.hg.1 | 0.00488 | 1.23868 | chr4(+):88896802-88904563 | Up |
| TC17001590.hg.1 | 0.00538 | 1.597801 | chr17(-):43037061-43045644 | Up |
| TC04000610.hg.1 | 0.00546 | 1.27618 | chr4(+):120056939-120108944 | Up |
| TC17002196.hg.1 | 0.00552 | 1.512376 | chr17(+):38249265-38249548 | Up |
| 2930629_st | 0.00669 | 1.026 | normgene->exon | Up |
| TC20000962.hg.1 | 0.00781 | 1.209613 | chr20(-):52769988-52790516 | Up |
| TC05002867.hg.1 | 0.008 | 1.010608 | chr5(-):9286515-9286787 | Up |
| TC10001522.hg.1 | 0.0081 | 1.182032 | chr10(-):93388197-93392858 | Up |
| TC08001295.hg.1 | 0.00867 | 1.098695 | chr8(-):68497704-68497734 | Up |
| TC04001188.hg.1 | 0.00923 | 1.090146 | chr4(-):49597627-49597709 | Up |
| TC04001190.hg.1 | 0.00923 | 1.090146 | chr4(-):49600440-49600522 | Up |
| TC17002618.hg.1 | 0.0094 | 1.115883 | chr17(-):38573069-38574167 | Up |
| TC15000159.hg.1 | 0.00972 | 1.240303 | chr15(+):30393356-30393381 | Up |
| TC15000185.hg.1 | 0.00972 | 1.240303 | chr15(+):30862353-30862378 | Up |
| TC15000960.hg.1 | 0.00972 | 1.240303 | chr15(+):100340437-100340462 | Up |
| TC15001139.hg.1 | 0.00972 | 1.240303 | chr15(-):30688271-30688296 | Up |
| TC15001173.hg.1 | 0.00972 | 1.240303 | chr15(-):32729620-32729645 | Up |
| TC07000294.hg.1 | 0.0102 | 1.118223 | chr7(+):45927956-45933267 | Up |
| TC12000189.hg.1 | 0.0103 | 1.011681 | chr12(+):13349602-13369708 | Up |
| 3597940_st | 0.0104 | 1.02089 | normgene->exon | Up |
| 3672401_st | 0.0106 | 1.057453 | normgene->exon | Up |
| TC09002209.hg.1 | 0.0109 | 1.241692 | chr9(+):118916071-118916321 | Up |
| TC01000904.hg.1 | 0.0128 | 1.108147 | chr1(+):101185196-101204601 | Up |
| 2999480_st | 0.016 | 1.321521 | normgene->exon | Up |
| 47420624_st | 0.0198 | 1.005919 | normgene->intron | Up |
| TC21000748.hg.1 | 0.021 | 1.00603 | chr21(+):43724173-43724497 | Up |
| 3305622_st | 0.0221 | 1.797215 | normgene->exon | Up |
| 2909168_st | 0.0327 | 1.036411 | normgene->exon | Up |
| 47421984_st | 0.0389 | 1.007156 | normgene->intron | Up |
| TC15002016.hg.1 | 0.0461 | 1.033327 | chr15(-):102292974-102293005 | Up |
| 2985876_st | 0.0462 | 1.459077 | normgene->exon | Up |
| 3597981_st | 0.0468 | 1.058749 | normgene->exon | Up |
| 47424481_st | 0.0472 | 1.300323 | normgene->intron | Up |
| TC17000542.hg.1 | 2.3E-12 | -4.31978 | chr17(+):41052815-41065386 | Down |
| TC17000230.hg.1 | 3.99E-12 | -4.25125 | chr17(+):19091329-19091544 | Down |
| TC17000226.hg.1 | 3.99E-12 | -4.10635 | chr17(+):18965225-18965440 | Down |
| TC17001231.hg.1 | 3.99E-12 | -4.10635 | chr17(-):18967234-18967449 | Down |
| TC17001233.hg.1 | 3.99E-12 | -3.94263 | chr17(-):19015734-19015949 | Down |
| TC17001238.hg.1 | 1.4E-11 | -3.88137 | chr17(-):19093343-19093558 | Down |
| TC10002662.hg.1 | 4.4E-10 | -1.28603 | chr10(-):61496635-61513203 | Down |
| TCUn_gl000220000001.hg.1 | 1.24E-09 | -1.40962 | chrUn_gl000220 | Down |
| TC14000142.hg.1 | 1.18E-08 | -1.3728 | chr14(+):23654525-23742686 | Down |
| TC10001316.hg.1 | 0.000000013 | -1.87729 | chr10(-):61496748-61513203 | Down |
| TC06001804.hg.1 | 2.05E-08 | -1.15131 | chr6(-):52696453-52710986 | Down |
| TC12001275.hg.1 | 2.78E-08 | -2.43502 | chr12(-):14978503-14996429 | Down |
| TC19000571.hg.1 | 4.41E-08 | -1.52797 | chr19(+):41497204-41524301 | Down |
| TC10000641.hg.1 | 4.57E-08 | -1.06545 | chr10(+):91215821-91227897 | Down |
| TC02002458.hg.1 | 7.27E-08 | -1.13076 | chr2(-):159027593-159313265 | Down |
| TC19001455.hg.1 | 0.000000103 | -1.69722 | chr19(-):36290892-36304201 | Down |
| TC12002777.hg.1 | 0.000000118 | -2.00382 | chr12(-):14996036-14996382 | Down |
| TC06001805.hg.1 | 0.000000119 | -1.13932 | chr6(-):52761437-52774496 | Down |
| TC12002013.hg.1 | 0.00000017 | -1.09177 | chr12(-):117581146-117628336 | Down |
| 3255819_st | 0.000000228 | -1.2498 | normgene->exon | Down |
| TC05001352.hg.1 | 0.000000312 | -3.16488 | chr5(-):54273692-54281491 | Down |
| TC12002776.hg.1 | 0.000000549 | -2.06301 | chr12(-):14982237-14993450 | Down |
| TC06001109.hg.1 | 0.000000549 | -1.61423 | chr6(+):153019030-153045853 | Down |
| TC18000947.hg.1 | 0.000000549 | -2.37551 | chr18(-):59475300-59476953 | Down |
| TC01004517.hg.1 | 0.000000589 | -1.99551 | chr1(+):89647181-89647394 | Down |
| TC13001414.hg.1 | 0.000000829 | -1.06719 | chr13(-):32599444-32605776 | Down |
| TC02004133.hg.1 | 0.000000975 | -3.89096 | chr2(-):28112323-28113981 | Down |
| TC17000033.hg.1 | 0.000000982 | -1.31794 | chr17(+):3377404-3406713 | Down |
| TC05002066.hg.1 | 0.00000126 | -1.17958 | chr5(-):172195093-172198203 | Down |
| TC0X000141.hg.1 | 0.00000153 | -1.13201 | chrX(+):30671476-30749577 | Down |
| TC05000609.hg.1 | 0.00000162 | -2.15299 | chr5(+):126984713-126994322 | Down |
| 3498866_st | 0.00000181 | -1.53425 | normgene->exon | Down |
| TC06001802.hg.1 | 0.00000183 | -2.67145 | chr6(-):52614885-52628367 | Down |
| TC13000547.hg.1 | 0.00000199 | -1.20714 | chr13(-):32598196-32605776 | Down |
| PSR11023102.hg.1 | 0.00000218 | -1.55898 | 19381056 | Down |
| TC20001450.hg.1 | 0.00000236 | -1.0787 | chr20(-):17922245-17922898 | Down |
| TC02003335.hg.1 | 0.00000245 | -1.0349 | chr2(+):74425690-74442424 | Down |
| TC0X001777.hg.1 | 0.00000263 | -1.1186 | chrX(+):75651475-75651742 | Down |
| 2912049_st | 0.00000268 | -1.34955 | normgene->exon | Down |
| TC15001612.hg.1 | 0.00000301 | -1.05642 | chr15(-):70946893-71055932 | Down |
| TC17002016.hg.1 | 0.00000302 | -1.5484 | chr17(+):3379296-3402700 | Down |
| PSR04008156.hg.1 | 0.00000365 | -1.0942 | 19164995 | Down |
| TC06001803.hg.1 | 0.00000461 | -2.14804 | chr6(-):52656178-52668708 | Down |
| TC12002263.hg.1 | 0.00000491 | -1.2004 | chr12(+):14927270-14930936 | Down |
| TC03001455.hg.1 | 0.00000491 | -2.01689 | chr3(-):52485107-52488086 | Down |
| TC04000403.hg.1 | 0.00000499 | -2.19873 | chr4(+):74262831-74287129 | Down |
| PSR18005234.hg.1 | 0.0000051 | -1.08066 | 19514123 | Down |
| TC04002929.hg.1 | 0.00000524 | -1.08073 | chr4(+):15704573-15739936 | Down |
| PSR02004944.hg.1 | 0.00000563 | -1.54729 | 19082110 | Down |
| TC01002708.hg.1 | 0.00000569 | -1.02924 | chr1(-):59246460-59249999 | Down |
| TC19001010.hg.1 | 0.00000594 | -1.29152 | chr19(-):1597154-1605483 | Down |
| TC18000551.hg.1 | 0.00000597 | -1.31178 | chr18(-):59475296-59560992 | Down |
| TC01003653.hg.1 | 0.00000734 | -1.5152 | chr1(-):193147860-193155784 | Down |
| 2824546_st | 0.00000778 | -1.09143 | normgene->exon | Down |
| PSR11032474.hg.1 | 0.00000784 | -1.38716 | 19389030 | Down |
| TC01002849.hg.1 | 0.00000795 | -1.34261 | chr1(-):89646831-89664633 | Down |
| TC09001381.hg.1 | 0.0000084 | -1.43517 | chr9(-):99791959-99822167 | Down |
| PSR11023103.hg.1 | 0.00000976 | -1.40611 | 19381057 | Down |
| TC01003920.hg.1 | 0.00000976 | -1.2219 | chr1(-):228746015-228746133 | Down |
| TC01003921.hg.1 | 0.00000976 | -1.2219 | chr1(-):228748256-228748374 | Down |
| TC01003922.hg.1 | 0.00000976 | -1.2219 | chr1(-):228750497-228750615 | Down |
| TC01003923.hg.1 | 0.00000976 | -1.2219 | chr1(-):228752738-228752856 | Down |
| TC01003924.hg.1 | 0.00000976 | -1.2219 | chr1(-):228754979-228755097 | Down |
| TC01003925.hg.1 | 0.00000976 | -1.2219 | chr1(-):228757194-228757312 | Down |
| TC01003926.hg.1 | 0.00000976 | -1.2219 | chr1(-):228759414-228759532 | Down |
| TC01003927.hg.1 | 0.00000976 | -1.2219 | chr1(-):228761656-228761774 | Down |
| TC01003929.hg.1 | 0.00000976 | -1.2219 | chr1(-):228766137-228766255 | Down |
| TC01003930.hg.1 | 0.00000976 | -1.2219 | chr1(-):228768378-228768496 | Down |
| TC01003931.hg.1 | 0.00000976 | -1.2219 | chr1(-):228770618-228770736 | Down |
| TC01003932.hg.1 | 0.00000976 | -1.2219 | chr1(-):228772843-228772961 | Down |
| TC01003933.hg.1 | 0.00000976 | -1.2219 | chr1(-):228775084-228775202 | Down |
| TC01003934.hg.1 | 0.00000976 | -1.2219 | chr1(-):228777315-228777433 | Down |
| TC01003935.hg.1 | 0.00000976 | -1.2219 | chr1(-):228779556-228779674 | Down |
| TC01003936.hg.1 | 0.00000976 | -1.2219 | chr1(-):228781787-228781905 | Down |
| TC09001337.hg.1 | 0.0000117 | -1.03292 | chr9(-):95255829-95298937 | Down |
| TC19001787.hg.1 | 0.0000124 | -1.40238 | chr19(-):52249023-52255150 | Down |
| 2835461_st | 0.0000141 | -1.23153 | normgene->exon | Down |
| PSR04015456.hg.1 | 0.0000144 | -1.25395 | 19170811 | Down |
| TC01006272.hg.1 | 0.0000162 | -1.33833 | chr1(+):78511586-78604133 | Down |
| TC15002310.hg.1 | 0.0000165 | -1.72885 | chr15(+):80253413-80263461 | Down |
| TC02001734.hg.1 | 0.0000179 | -1.15131 | chr2(-):36581892-36582713 | Down |
| TC10001696.hg.1 | 0.0000185 | -1.06998 | chr10(-):120631561-120631989 | Down |
| TC11000480.hg.1 | 0.0000185 | -1.60581 | chr11(+):58695102-58724543 | Down |
| TC14000471.hg.1 | 0.0000185 | -2.82603 | chr14(+):75745477-75748937 | Down |
| TC0X001533.hg.1 | 0.0000195 | -1.13483 | chrX(-):153652728-153656796 | Down |
| 3644241_st | 0.00002 | -1.18991 | normgene->exon | Down |
| 3606732_st | 0.000021 | -1.31769 | normgene->exon | Down |
| TC01000780.hg.1 | 0.0000211 | -1.74514 | chr1(+):76507376-76531913 | Down |
| PSR15022167.hg.1 | 0.0000216 | -1.02206 | 19465128 | Down |
| TC12001274.hg.1 | 0.0000216 | -1.19306 | chr12(-):14957584-14967116 | Down |
| TC04002626.hg.1 | 0.0000219 | -1.27542 | chr4(-):89178768-89180508 | Down |
| TC15001537.hg.1 | 0.0000238 | -1.4869 | chr15(-):63134323-63136780 | Down |
| TC16001253.hg.1 | 0.0000255 | -1.8995 | chr16(-):71792305-71792390 | Down |
| TC08002421.hg.1 | 0.0000263 | -1.20165 | chr8(-):92968730-92970161 | Down |
| TC15002232.hg.1 | 0.0000266 | -1.56661 | chr15(+):63135274-63136830 | Down |
| TC17000050.hg.1 | 0.0000271 | -1.31044 | chr17(+):4675187-4686508 | Down |
| PSR11018241.hg.1 | 0.0000287 | -1.26221 | 19377240 | Down |
| PSR18005235.hg.1 | 0.0000301 | -1.08808 | 19514124 | Down |
| TC01006108.hg.1 | 0.0000342 | -1.01761 | chr1(-):214522047-214523573 | Down |
| TC09001357.hg.1 | 0.0000359 | -1.31476 | chr9(-):97365415-97402531 | Down |
| TC07001618.hg.1 | 0.0000391 | -2.38729 | chr7(-):95212809-95225925 | Down |
| TC06000978.hg.1 | 0.0000394 | -1.23108 | chr6(+):130334844-130462594 | Down |
| TC02001696.hg.1 | 0.0000394 | -2.40265 | chr2(-):28112323-28113981 | Down |
| TC09002178.hg.1 | 0.0000394 | -1.06609 | chr9(+):111781216-111781846 | Down |
| TC08002148.hg.1 | 0.0000452 | -1.98244 | chr8(+):144298519-144299053 | Down |
| TC03002458.hg.1 | 0.0000483 | -1.14476 | chr3(+):108855561-108868951 | Down |
| TC04001372.hg.1 | 0.0000494 | -1.22431 | chr4(-):89178768-89180508 | Down |
| TC08000004.hg.1 | 0.0000505 | -1.0325 | chr8(+):424455-427966 | Down |
| TC04000599.hg.1 | 0.0000604 | -1.61508 | chr4(+):119199914-119200978 | Down |
| TC20000450.hg.1 | 0.0000604 | -1.34214 | chr20(+):56131260-56141513 | Down |
| TC17001810.hg.1 | 0.0000606 | -1.29825 | chr17(-):64208147-64225556 | Down |
| TC20001544.hg.1 | 0.0000625 | -1.09129 | chr20(-):32291920-32292454 | Down |
| TC06002715.hg.1 | 0.000063 | -1.48973 | chr6(+):31553956-31556686 | Down |
| TC04002157.hg.1 | 0.0000646 | -1.59472 | chr4(+):119199914-119200978 | Down |
| TC04000405.hg.1 | 0.000066 | -1.58573 | chr4(+):74347400-74369718 | Down |
| TC01005554.hg.1 | 0.0000691 | -1.77451 | chr1(-):71472542-71473814 | Down |
| TC12003032.hg.1 | 0.0000757 | -1.0023 | chr12(-):98943554-98943953 | Down |
| TC06003064.hg.1 | 0.000076 | -1.1803 | chr6(+):134309210-134311566 | Down |
| TC12002039.hg.1 | 0.0000806 | -1.41216 | chr12(-):120729566-120729706 | Down |
| TC18000515.hg.1 | 0.000082 | -1.17003 | chr18(-):48321490-48351754 | Down |
| TC01005928.hg.1 | 0.0000942 | -1.18087 | chr1(-):169659806-169677997 | Down |
| TC14001208.hg.1 | 0.0000959 | -1.11436 | chr14(-):64118015-64118217 | Down |
| TC18000129.hg.1 | 0.0000964 | -1.60442 | chr18(+):29171689-29178986 | Down |
| TC17001093.hg.1 | 0.0000996 | -1.06133 | chr17(-):7529552-7531194 | Down |
| TC04002167.hg.1 | 0.000109 | -1.20214 | chr4(+):119957649-119959674 | Down |
| TC11002706.hg.1 | 0.00011 | -1.00838 | chr11(+):62529072-62534182 | Down |
| TC06003720.hg.1 | 0.000112 | -1.0086 | chr6(-):76001574-76003213 | Down |
| TC15002198.hg.1 | 0.000119 | -1.18725 | chr15(+):52107460-52108108 | Down |
| TC08001461.hg.1 | 0.000127 | -1.30203 | chr8(-):99114567-99129469 | Down |
| PSR05025587.hg.1 | 0.000128 | -1.06377 | 19203049 | Down |
| PSR02000273.hg.1 | 0.000152 | -1.06625 | 19078441 | Down |
| TC14000936.hg.1 | 0.000154 | -1.14273 | chr14(-):23242431-23299029 | Down |
| TC19002215.hg.1 | 0.000154 | -1.56894 | chr19(+):52249199-52250247 | Down |
| TC01005268.hg.1 | 0.000158 | -1.22993 | chr1(-):12640653-12677348 | Down |
| TC06001225.hg.1 | 0.00016 | -1.04773 | chr6(-):3138628-3153296 | Down |
| TC07001786.hg.1 | 0.000163 | -1.26679 | chr7(-):116203648-116254874 | Down |
| TC16001930.hg.1 | 0.000167 | -1.1355 | chr16(-):66965958-66968320 | Down |
| TC06001608.hg.1 | 0.000188 | -1.99772 | chr6(-):33842349-33842378 | Down |
| TC17001547.hg.1 | 0.000206 | -1.103 | chr17(-):41043355-41043452 | Down |
| 3090546_st | 0.000223 | -1.00875 | normgene->exon | Down |
| TC10002643.hg.1 | 0.000227 | -1.23413 | chr10(-):52559793-52565728 | Down |
| PSR06024774.hg.1 | 0.000235 | -1.16695 | 19231186 | Down |
| TC14001926.hg.1 | 0.000236 | -1.71471 | chr14(-):23242432-23285101 | Down |
| TC01003261.hg.1 | 0.000239 | -1.31052 | chr1(-):153362508-153363664 | Down |
| TC6_mcf_hap5000046.hg.1 | 0.000253 | -1.14511 | chr6_mcf_hap5 | Down |
| TC14001941.hg.1 | 0.000259 | -1.21929 | chr14(-):27244062-27291394 | Down |
| TC22000715.hg.1 | 0.000274 | -1.01216 | chr22(-):37196728-37215523 | Down |
| TC03001001.hg.1 | 0.00029 | -1.1811 | chr3(+):184043484-184043559 | Down |
| TC09002408.hg.1 | 0.000308 | -1.13353 | chr9(-):14081848-14082201 | Down |
| PSR02004940.hg.1 | 0.000336 | -1.08978 | 19082106 | Down |
| TC13001275.hg.1 | 0.000369 | -1.29315 | chr13(+):107279173-107284309 | Down |
| TC17001970.hg.1 | 0.000403 | -1.20607 | chr17(-):79993757-79995573 | Down |
| TC05000701.hg.1 | 0.000405 | -3.55767 | chr5(+):137801169-137805004 | Down |
| TC17002831.hg.1 | 0.000405 | -1.1619 | chr17(-):79993720-79995579 | Down |
| TC16001990.hg.1 | 0.000406 | -1.65453 | chr16(-):84649611-84650115 | Down |
| TC14001732.hg.1 | 0.000406 | -1.46147 | chr14(+):65400531-65402086 | Down |
| TC19001995.hg.1 | 0.000413 | -1.76898 | chr19(+):12902290-12902585 | Down |
| TC09001201.hg.1 | 0.000414 | -1.10365 | chr9(-):72999503-73029573 | Down |
| 3545877_st | 0.000432 | -1.28371 | normgene->exon | Down |
| TC07003127.hg.1 | 0.000442 | -1.04385 | chr7(-):116203648-116254874 | Down |
| PSR12027032.hg.1 | 0.000485 | -1.02105 | 19412064 | Down |
| TC15002231.hg.1 | 0.000486 | -1.54897 | chr15(+):63133190-63133700 | Down |
| TC06002112.hg.1 | 0.000506 | -1.32207 | chr6(-):132269316-132272518 | Down |
| TC11002391.hg.1 | 0.00053 | -1.76689 | chr11(-):122928785-122928869 | Down |
| PSR05013780.hg.1 | 0.000551 | -1.18007 | 19193895 | Down |
| TC12002835.hg.1 | 0.000555 | -1.69942 | chr12(-):40148896-40150095 | Down |
| TC01003900.hg.1 | 0.000604 | -1.12321 | chr1(-):226723319-226730469 | Down |
| TC04000568.hg.1 | 0.000674 | -1.36376 | chr4(+):110834040-110934118 | Down |
| TC01001346.hg.1 | 0.000678 | -1.38954 | chr1(+):158801107-158819296 | Down |
| TC06002124.hg.1 | 0.000691 | -1.01411 | chr6(-):134308719-134373789 | Down |
| PSR16011591.hg.1 | 0.000702 | -1.36469 | 19474748 | Down |
| TC20001590.hg.1 | 0.000752 | -1.65011 | chr20(-):42985322-42987684 | Down |
| TC08000906.hg.1 | 0.000855 | -1.98397 | chr8(-):6728097-6735544 | Down |
| TC08001960.hg.1 | 0.000924 | -1.19147 | chr8(+):69146910-69149265 | Down |
| TC11002891.hg.1 | 0.000947 | -2.22105 | chr11(+):116703533-116703783 | Down |
| TC11001330.hg.1 | 0.000968 | -1.60431 | chr11(-):5246694-5250625 | Down |
| 3644269_st | 0.00105 | -1.43179 | normgene->exon | Down |
| TCUn_gl000220000002.hg.1 | 0.00106 | -1.10803 | chrUn_gl000220 | Down |
| TC04001262.hg.1 | 0.00106 | -1.04648 | chr4(-):69780640-69780918 | Down |
| TC14000989.hg.1 | 0.00107 | -1.42527 | chr14(-):27244701-27291313 | Down |
| TC14001578.hg.1 | 0.00109 | -1.1113 | chr14(+):20944648-20945246 | Down |
| TC02003832.hg.1 | 0.00113 | -1.60606 | chr2(+):218999678-219000012 | Down |
| TC14001303.hg.1 | 0.00115 | -1.06281 | chr14(-):74523553-74551196 | Down |
| TC16000007.hg.1 | 0.00115 | -1.22158 | chr16(+):222846-223709 | Down |
| TC01001037.hg.1 | 0.00117 | -1.64599 | chr1(+):119911402-119936753 | Down |
| TC12001892.hg.1 | 0.00127 | -1.44556 | chr12(-):103230663-103311381 | Down |
| TC01004452.hg.1 | 0.00127 | -1.25464 | chr1(+):65695311-65697819 | Down |
| TC19000228.hg.1 | 0.0014 | -1.77199 | chr19(+):12902310-12904125 | Down |
| TC05001951.hg.1 | 0.00143 | -1.37125 | chr5(-):150694539-150727151 | Down |
| TC06000377.hg.1 | 0.00144 | -1.19531 | chr6(+):31620187-31625987 | Down |
| TC06002823.hg.1 | 0.00154 | -1.71642 | chr6(+):50061424-50066995 | Down |
| TC01003260.hg.1 | 0.00158 | -2.05016 | chr1(-):153346181-153348125 | Down |
| 3234823_st | 0.0016 | -1.35596 | normgene->exon | Down |
| TC09000358.hg.1 | 0.0016 | -1.2925 | chr9(+):80912043-80945009 | Down |
| TC04001002.hg.1 | 0.00162 | -1.16175 | chr4(-):6672452-6675557 | Down |
| TC04002064.hg.1 | 0.00162 | -1.67466 | chr4(+):74347481-74350046 | Down |
| TC06001607.hg.1 | 0.00167 | -1.22085 | chr6(-):33842074-33842118 | Down |
| TC02003949.hg.1 | 0.00168 | -1.00601 | chr2(-):218149-264392 | Down |
| TC05002437.hg.1 | 0.00172 | -1.21557 | chr5(+):71735735-71736030 | Down |
| TC11002771.hg.1 | 0.00179 | -1.11362 | chr11(+):71713291-71713581 | Down |
| TC01003638.hg.1 | 0.00193 | -1.34611 | chr1(-):186640923-186649559 | Down |
| TC16000008.hg.1 | 0.00201 | -1.57281 | chr16(+):226679-227521 | Down |
| TC0X001278.hg.1 | 0.00208 | -1.02866 | chrX(-):109917084-110039286 | Down |
| TC06000644.hg.1 | 0.00211 | -1.69704 | chr6(+):50061424-50066995 | Down |
| TC05001260.hg.1 | 0.0022 | -1.13589 | chr5(-):34998206-35048240 | Down |
| TC6_cox_hap2000072.hg.1 | 0.0022 | -1.34361 | chr6_cox_hap2 | Down |
| TC6_dbb_hap3000063.hg.1 | 0.0022 | -1.34361 | chr6_dbb_hap3 | Down |
| TC6_mann_hap4000063.hg.1 | 0.0022 | -1.34361 | chr6_mann_hap4 | Down |
| TC6_mcf_hap5000058.hg.1 | 0.0022 | -1.34361 | chr6_mcf_hap5 | Down |
| TC6_qbl_hap6000063.hg.1 | 0.0022 | -1.34361 | chr6_qbl_hap6 | Down |
| TC6_ssto_hap7000059.hg.1 | 0.0023 | -1.32302 | chr6_ssto_hap7 | Down |
| TC14001821.hg.1 | 0.0024 | -1.45056 | chr14(+):94577084-94582176 | Down |
| TC10002485.hg.1 | 0.00246 | -1.23089 | chr10(-):16865965-16866360 | Down |
| TC12001366.hg.1 | 0.00249 | -1.19734 | chr12(-):31768702-31769373 | Down |
| TC05000346.hg.1 | 0.00253 | -1.43783 | chr5(+):72464074-72470970 | Down |
| TC01002246.hg.1 | 0.00304 | -1.26933 | chr1(-):15898194-15911605 | Down |
| TC14001604.hg.1 | 0.00344 | -1.01462 | chr14(+):24464307-24475157 | Down |
| TC14000584.hg.1 | 0.0036 | -1.15628 | chr14(+):94577079-94583033 | Down |
| TC11003023.hg.1 | 0.00377 | -1.24666 | chr11(-):5246697-5246908 | Down |
| TC21000363.hg.1 | 0.00409 | -1.22581 | chr21(-):31586324-31588469 | Down |
| TC01005690.hg.1 | 0.00464 | -1.04275 | chr1(-):110276554-110283660 | Down |
| TC01001749.hg.1 | 0.0047 | -1.08896 | chr1(+):209848670-209849735 | Down |
| TC22000427.hg.1 | 0.00492 | -1.47055 | chr22(+):50925213-50929077 | Down |
| TC01001488.hg.1 | 0.00494 | -1.08995 | chr1(+):171154347-171181822 | Down |
| 2960238_st | 0.00538 | -1.38197 | normgene->exon | Down |
| TC16001729.hg.1 | 0.00538 | -1.72871 | chr16(-):223162-223620 | Down |
| TC12001890.hg.1 | 0.00559 | -2.09392 | chr12(-):102789645-102874423 | Down |
| TC17001341.hg.1 | 0.00659 | -1.04363 | chr17(-):29630788-29641130 | Down |
| TC02004392.hg.1 | 0.00724 | -1.33942 | chr2(-):88422556-88427635 | Down |
| 3374448_st | 0.00743 | -1.0479 | normgene->exon | Down |
| TC04002840.hg.1 | 0.00783 | -1.49684 | chr4(-):175411328-175444044 | Down |
| TC04001753.hg.1 | 0.00835 | -1.0977 | chr4(-):175411328-175444305 | Down |
| TC04000371.hg.1 | 0.00855 | -1.17221 | chr4(+):69917081-69978705 | Down |
| 3183477_st | 0.009 | -1.26578 | normgene->exon | Down |
| TC0X001920.hg.1 | 0.009 | -1.33761 | chrX(+):151928402-151928613 | Down |
| TC12003046.hg.1 | 0.00929 | -1.60534 | chr12(-):102718465-102745141 | Down |
| TC02001498.hg.1 | 0.00938 | -1.3338 | chr2(-):490944-492655 | Down |
| TC01003749.hg.1 | 0.0094 | -1.01618 | chr1(-):205523401-205525763 | Down |
| TC6_dbb_hap3000180.hg.1 | 0.00996 | -1.43097 | chr6_dbb_hap3 | Down |
| TC01004998.hg.1 | 0.0103 | -1.25667 | chr1(+):209849031-209849338 | Down |
| TC11002528.hg.1 | 0.0107 | -1.50396 | chr11(+):3536784-3542040 | Down |
| TC01001254.hg.1 | 0.0113 | -1.64204 | chr1(+):153330330-153333503 | Down |
| TC11002606.hg.1 | 0.0113 | -1.19118 | chr11(+):27148999-27149235 | Down |
| TC15002182.hg.1 | 0.0116 | -1.14663 | chr15(+):45654024-45654249 | Down |
| TC03002341.hg.1 | 0.0118 | -1.03657 | chr3(+):52009042-52023218 | Down |
| PSR19000800.hg.1 | 0.0122 | -1.19496 | 19517255 | Down |
| TC01001174.hg.1 | 0.0127 | -1.2523 | chr1(+):149822628-149823191 | Down |
| TC02004144.hg.1 | 0.0131 | -1.05312 | chr2(-):29319709-29320391 | Down |
| TC11001813.hg.1 | 0.0137 | -1.17695 | chr11(-):58476230-58499447 | Down |
| TC01006058.hg.1 | 0.0138 | -1.22538 | chr1(-):205523401-205525763 | Down |
| TC01001695.hg.1 | 0.0144 | -1.00702 | chr1(+):204100190-204101094 | Down |
| TC05001237.hg.1 | 0.0145 | -1.64333 | chr5(-):32646670-32652082 | Down |
| TC02001707.hg.1 | 0.0157 | -1.0231 | chr2(-):29319709-29320391 | Down |
| TC19002559.hg.1 | 0.0166 | -1.08751 | chr19(-):51874876-51875969 | Down |
| 3080354_st | 0.0169 | -1.04575 | normgene->exon | Down |
| TC08000259.hg.1 | 0.0178 | -1.17072 | chr8(+):33370993-33371096 | Down |
| TC03001024.hg.1 | 0.019 | -1.75205 | chr3(+):186435065-186462199 | Down |
| TC19000544.hg.1 | 0.0197 | -1.81316 | chr19(+):39897487-39900047 | Down |
| 3447838_st | 0.0199 | -1.28791 | normgene->exon | Down |
| TC04002581.hg.1 | 0.02 | -1.00679 | chr4(-):69914349-69915748 | Down |
| TC01002166.hg.1 | 0.0229 | -1.04895 | chr1(-):8064464-8086393 | Down |
| TC19001621.hg.1 | 0.0231 | -2.74224 | chr19(-):45976341-45978414 | Down |
| TC01002412.hg.1 | 0.0274 | -1.50567 | chr1(-):27992572-27998729 | Down |
| TC19002172.hg.1 | 0.0282 | -2.87636 | chr19(+):45978197-45978439 | Down |
| 3437245_st | 0.0288 | -1.34356 | normgene->exon | Down |
| TC12001217.hg.1 | 0.0303 | -1.35258 | chr12(-):10331631-10342707 | Down |
| TC12002066.hg.1 | 0.0307 | -1.18598 | chr12(-):122277433-122326517 | Down |
| TC19002521.hg.1 | 0.0343 | -2.26834 | chr19(-):45976341-45978414 | Down |
| 47419822_st | 0.0352 | -1.00611 | normgene->intron | Down |
| TC11000478.hg.1 | 0.0401 | -1.13988 | chr11(+):58645775-58660445 | Down |
| TC03001719.hg.1 | 0.0418 | -1.20273 | chr3(-):124624289-124672663 | Down |

**Supplementary Table 3. The items of BP in GO enrichment of the targets of YSHS granule against FSGS.**

| **ID** | **Description** | **Count** | **P Value** |
| --- | --- | --- | --- |
| GO:0035630 | Bone mineralization involved in bone maturation | 2 | 3.88E-05 |
| GO:0034308 | Primary alcohol metabolic process | 3 | 5.22E-05 |
| GO:0098869 | Cellular oxidant detoxification | 3 | 6.86E-05 |
| GO:1990748 | Cellular detoxification | 3 | 9.27E-05 |
| GO:0043931 | Ossification involved in bone maturation | 2 | 0.000111 |
| GO:0097237 | Cellular response to toxic substance | 3 | 0.000113 |
| GO:0043500 | Muscle adaptation | 3 | 0.000122 |
| GO:1901685 | Glutathione derivative metabolic process | 2 | 0.000135 |
| GO:1901687 | Glutathione derivative biosynthetic process | 2 | 0.000135 |
| GO:0070977 | Bone maturation | 2 | 0.000148 |
| GO:0098754 | Detoxification | 3 | 0.000163 |
| GO:0043567 | Regulation of insulin-like growth factor receptor signaling pathway | 2 | 0.00019 |
| GO:0006066 | Alcohol metabolic process | 4 | 0.000195 |
| GO:0048799 | Animal organ maturation | 2 | 0.000205 |
| GO:0009267 | Cellular response to starvation | 3 | 0.000267 |
| GO:0048009 | Insulin-like growth factor receptor signaling pathway | 2 | 0.000408 |
| GO:0006575 | Cellular modified amino acid metabolic process | 3 | 0.000418 |
| GO:0042594 | Response to starvation | 3 | 0.00053 |
| GO:0006006 | Glucose metabolic process | 3 | 0.000592 |
| GO:0031669 | Cellular response to nutrient levels | 3 | 0.00065 |
| GO:0042445 | Hormone metabolic process | 3 | 0.00065 |
| GO:2000725 | Regulation of cardiac muscle cell differentiation | 2 | 0.000707 |
| GO:0050435 | Amyloid-beta metabolic process | 2 | 0.000736 |
| GO:0048738 | Cardiac muscle tissue development | 3 | 0.000739 |
| GO:0071695 | Anatomical structure maturation | 3 | 0.000777 |
| GO:0006749 | Glutathione metabolic process | 2 | 0.000824 |
| GO:0009636 | Response to toxic substance | 3 | 0.00093 |
| GO:0031668 | Cellular response to extracellular stimulus | 3 | 0.000962 |
| GO:0019318 | Hexose metabolic process | 3 | 0.000973 |
| GO:1901617 | Organic hydroxy compound biosynthetic process | 3 | 0.001018 |
| GO:0055008 | Cardiac muscle tissue morphogenesis | 2 | 0.001121 |
| GO:0045669 | Positive regulation of osteoblast differentiation | 2 | 0.001229 |
| GO:1905207 | Regulation of cardiocyte differentiation | 2 | 0.001229 |
| GO:0016054 | Organic acid catabolic process | 3 | 0.001316 |
| GO:0046395 | Carboxylic acid catabolic process | 3 | 0.001316 |
| GO:0021700 | Developmental maturation | 3 | 0.001384 |
| GO:0005996 | Monosaccharide metabolic process | 3 | 0.001512 |
| GO:0060415 | Muscle tissue morphogenesis | 2 | 0.001625 |
| GO:0048644 | Muscle organ morphogenesis | 2 | 0.001935 |
| GO:0048708 | Astrocyte differentiation | 2 | 0.001935 |
| GO:0071496 | Cellular response to external stimulus | 3 | 0.001994 |
| GO:0034637 | Cellular carbohydrate biosynthetic process | 2 | 0.002075 |
| GO:0032496 | Response to lipopolysaccharide | 3 | 0.002136 |
| GO:0006029 | Proteoglycan metabolic process | 2 | 0.00227 |
| GO:0045778 | Positive regulation of ossification | 2 | 0.002472 |
| GO:0051591 | Response to cAMP | 2 | 0.002472 |
| GO:0002237 | Response to molecule of bacterial origin | 3 | 0.002561 |
| GO:0051251 | Positive regulation of lymphocyte activation | 3 | 0.002582 |
| GO:0007159 | Leukocyte cell-cell adhesion | 3 | 0.002728 |
| GO:0050848 | Regulation of calcium-mediated signaling | 2 | 0.002792 |
| GO:0043502 | Regulation of muscle adaptation | 2 | 0.002903 |
| GO:0055024 | Regulation of cardiac muscle tissue development | 2 | 0.002903 |
| GO:0001823 | Mesonephros development | 2 | 0.002959 |
| GO:0030593 | Neutrophil chemotaxis | 2 | 0.002959 |
| GO:0014706 | Striated muscle tissue development | 3 | 0.003291 |
| GO:0002696 | Positive regulation of leukocyte activation | 3 | 0.003713 |
| GO:0007517 | Muscle organ development | 3 | 0.003739 |
| GO:0060537 | Muscle tissue development | 3 | 0.003791 |
| GO:0030282 | Bone mineralization | 2 | 0.003798 |
| GO:0006805 | Xenobiotic metabolic process | 2 | 0.003862 |
| GO:0051153 | Regulation of striated muscle cell differentiation | 2 | 0.003926 |
| GO:0120254 | Olefinic compound metabolic process | 2 | 0.004056 |
| GO:0050867 | Positive regulation of cell activation | 3 | 0.004111 |
| GO:1990266 | Neutrophil migration | 2 | 0.004122 |
| GO:0071466 | Cellular response to xenobiotic stimulus | 2 | 0.004188 |
| GO:0050727 | Regulation of inflammatory response | 3 | 0.004222 |
| GO:0055007 | Cardiac muscle cell differentiation | 2 | 0.004389 |
| GO:0071621 | Granulocyte chemotaxis | 2 | 0.004458 |
| GO:0002576 | Platelet degranulation | 2 | 0.004596 |
| GO:0022407 | Regulation of cell-cell adhesion | 3 | 0.004623 |
| GO:0009410 | Response to xenobiotic stimulus | 2 | 0.004665 |
| GO:0045667 | Regulation of osteoblast differentiation | 2 | 0.004735 |
| GO:0034754 | Cellular hormone metabolic process | 2 | 0.004949 |
| GO:0046683 | Response to organophosphorus | 2 | 0.004949 |
| GO:0044282 | Small molecule catabolic process | 3 | 0.005015 |
| GO:0050671 | Positive regulation of lymphocyte proliferation | 2 | 0.005093 |
| GO:0032946 | Positive regulation of mononuclear cell proliferation | 2 | 0.005167 |
| GO:0072329 | Monocarboxylic acid catabolic process | 2 | 0.00524 |
| GO:0008584 | Male gonad development | 2 | 0.005464 |
| GO:0003012 | Muscle system process | 3 | 0.005493 |
| GO:0046546 | Development of primary male sexual characteristics | 2 | 0.005539 |
| GO:0031667 | Response to nutrient levels | 3 | 0.005692 |
| GO:0051384 | Response to glucocorticoid | 2 | 0.005924 |
| GO:0014074 | Response to purine-containing compound | 2 | 0.006081 |
| GO:0070665 | Positive regulation of leukocyte proliferation | 2 | 0.006161 |
| GO:0097530 | Granulocyte migration | 2 | 0.006161 |
| GO:0043312 | Neutrophil degranulation | 3 | 0.006172 |
| GO:0002283 | Neutrophil activation involved in immune response | 3 | 0.006278 |
| GO:0006090 | Pyruvate metabolic process | 2 | 0.006321 |
| GO:0016202 | Regulation of striated muscle tissue development | 2 | 0.006402 |
| GO:0000187 | Activation of MAPK activity | 2 | 0.006647 |
| GO:1901861 | Regulation of muscle tissue development | 2 | 0.006647 |
| GO:0048634 | Regulation of muscle organ development | 2 | 0.00673 |
| GO:0035051 | Cardiocyte differentiation | 2 | 0.006813 |
| GO:0031214 | Biomineral tissue development | 2 | 0.007236 |
| GO:0110148 | Biomineralization | 2 | 0.007236 |
| GO:0031960 | Response to corticosteroid | 2 | 0.007322 |
| GO:0046165 | Alcohol biosynthetic process | 2 | 0.007322 |
| GO:0046661 | Male sex differentiation | 2 | 0.007322 |
| GO:0003129 | Heart induction | 1 | 0.007924 |
| GO:0003307 | Regulation of Wnt signaling pathway involved in heart development | 1 | 0.007924 |
| GO:0031943 | Regulation of glucocorticoid metabolic process | 1 | 0.007924 |
| GO:0032341 | Aldosterone metabolic process | 1 | 0.007924 |
| GO:0032342 | Aldosterone biosynthetic process | 1 | 0.007924 |
| GO:0034651 | Cortisol biosynthetic process | 1 | 0.007924 |
| GO:0042756 | Drinking behavior | 1 | 0.007924 |
| GO:0043380 | Regulation of memory T cell differentiation | 1 | 0.007924 |
| GO:0070327 | Thyroid hormone transport | 1 | 0.007924 |
| GO:1902645 | Tertiary alcohol biosynthetic process | 1 | 0.007924 |
| GO:0030307 | Positive regulation of cell growth | 2 | 0.007936 |
| GO:0051099 | Positive regulation of binding | 2 | 0.008207 |
| GO:0006558 | L-phenylalanine metabolic process | 1 | 0.008714 |
| GO:0006559 | L-phenylalanine catabolic process | 1 | 0.008714 |
| GO:0006570 | Tyrosine metabolic process | 1 | 0.008714 |
| GO:0014889 | Muscle atrophy | 1 | 0.008714 |
| GO:0034650 | Cortisol metabolic process | 1 | 0.008714 |
| GO:0042482 | Positive regulation of odontogenesis | 1 | 0.008714 |
| GO:0043379 | Memory T cell differentiation | 1 | 0.008714 |
| GO:0048102 | Autophagic cell death | 1 | 0.008714 |
| GO:0060281 | Regulation of oocyte development | 1 | 0.008714 |
| GO:0090030 | Regulation of steroid hormone biosynthetic process | 1 | 0.008714 |
| GO:0090715 | Immunological memory formation process | 1 | 0.008714 |
| GO:1902221 | Erythrose 4-phosphate/phosphoenolpyruvate family amino acid metabolic process | 1 | 0.008714 |
| GO:1902222 | Erythrose 4-phosphate/phosphoenolpyruvate family amino acid catabolic process | 1 | 0.008714 |
| GO:1905879 | Regulation of oogenesis | 1 | 0.008714 |
| GO:0051147 | Regulation of muscle cell differentiation | 2 | 0.00933 |
| GO:0002002 | Regulation of angiotensin levels in blood | 1 | 0.009502 |
| GO:0002003 | Angiotensin maturation | 1 | 0.009502 |
| GO:0033690 | Positive regulation of osteoblast proliferation | 1 | 0.009502 |
| GO:0051657 | Maintenance of organelle location | 1 | 0.009502 |
| GO:0071468 | Cellular response to acidic pH | 1 | 0.009502 |
| GO:0044272 | Sulfur compound biosynthetic process | 2 | 0.009916 |
| GO:0006705 | Mineralocorticoid biosynthetic process | 1 | 0.01029 |
| GO:0008212 | Mineralocorticoid metabolic process | 1 | 0.01029 |
| GO:0021978 | Telencephalon regionalization | 1 | 0.01029 |
| GO:0021984 | Adenohypophysis development | 1 | 0.01029 |
| GO:0034309 | Primary alcohol biosynthetic process | 1 | 0.01029 |
| GO:0043568 | Positive regulation of insulin-like growth factor receptor signaling pathway | 1 | 0.01029 |
| GO:0048711 | Positive regulation of astrocyte differentiation | 1 | 0.01029 |
| GO:0009749 | Response to glucose | 2 | 0.010417 |
| GO:0016052 | Carbohydrate catabolic process | 2 | 0.010722 |
| GO:0009746 | Response to hexose | 2 | 0.010928 |
| GO:0003306 | Wnt signaling pathway involved in heart development | 1 | 0.011078 |
| GO:0034392 | Negative regulation of smooth muscle cell apoptotic process | 1 | 0.011078 |
| GO:0061051 | Positive regulation of cell growth involved in cardiac muscle cell development | 1 | 0.011078 |
| GO:0070486 | Leukocyte aggregation | 1 | 0.011078 |
| GO:0090713 | Immunological memory process | 1 | 0.011078 |
| GO:1902430 | Negative regulation of amyloid-beta formation | 1 | 0.011078 |
| GO:0060348 | Bone development | 2 | 0.011136 |
| GO:0034284 | Response to monosaccharide | 2 | 0.011451 |
| GO:0046890 | Regulation of lipid biosynthetic process | 2 | 0.011451 |
| GO:0014733 | Regulation of skeletal muscle adaptation | 1 | 0.011864 |
| GO:0019321 | Pentose metabolic process | 1 | 0.011864 |
| GO:0046184 | Aldehyde biosynthetic process | 1 | 0.011864 |
| GO:0070365 | Hepatocyte differentiation | 1 | 0.011864 |
| GO:2000846 | Regulation of corticosteroid hormone secretion | 1 | 0.011864 |
| GO:2001044 | Regulation of integrin-mediated signaling pathway | 1 | 0.011864 |
| GO:0050679 | Positive regulation of epithelial cell proliferation | 2 | 0.011877 |
| GO:0030278 | Regulation of ossification | 2 | 0.011985 |
| GO:0016051 | Carbohydrate biosynthetic process | 2 | 0.012093 |
| GO:0050870 | Positive regulation of T cell activation | 2 | 0.012202 |
| GO:0051651 | Maintenance of location in cell | 2 | 0.01242 |
| GO:0002523 | Leukocyte migration involved in inflammatory response | 1 | 0.012651 |
| GO:0017014 | Protein nitrosylation | 1 | 0.012651 |
| GO:0018119 | Peptidyl-cysteine S-nitrosylation | 1 | 0.012651 |
| GO:0035930 | Corticosteroid hormone secretion | 1 | 0.012651 |
| GO:0042487 | Regulation of odontogenesis of dentin-containing tooth | 1 | 0.012651 |
| GO:0045725 | Positive regulation of glycogen biosynthetic process | 1 | 0.012651 |
| GO:0051238 | Sequestering of metal ion | 1 | 0.012651 |
| GO:0070886 | Positive regulation of calcineurin-NFAT signaling cascade | 1 | 0.012651 |
| GO:0106058 | Positive regulation of calcineurin-mediated signaling | 1 | 0.012651 |
| GO:1903729 | Regulation of plasma membrane organization | 1 | 0.012651 |
| GO:0050670 | Regulation of lymphocyte proliferation | 2 | 0.012751 |
| GO:0032944 | Regulation of mononuclear cell proliferation | 2 | 0.012973 |
| GO:0019722 | Calcium-mediated signaling | 2 | 0.013085 |
| GO:0097529 | Myeloid leukocyte migration | 2 | 0.013085 |
| GO:0008406 | Gonad development | 2 | 0.013198 |
| GO:0003128 | Heart field specification | 1 | 0.013436 |
| GO:0010566 | Regulation of ketone biosynthetic process | 1 | 0.013436 |
| GO:0046459 | Short-chain fatty acid metabolic process | 1 | 0.013436 |
| GO:0070875 | Positive regulation of glycogen metabolic process | 1 | 0.013436 |
| GO:1902931 | Negative regulation of alcohol biosynthetic process | 1 | 0.013436 |
| GO:1902992 | Negative regulation of amyloid precursor protein catabolic process | 1 | 0.013436 |
| GO:0045137 | Development of primary sexual characteristics | 2 | 0.013881 |
| GO:0010001 | Glial cell differentiation | 2 | 0.013996 |
| GO:0001649 | Osteoblast differentiation | 2 | 0.014112 |
| GO:0034375 | High-density lipoprotein particle remodeling | 1 | 0.014222 |
| GO:0042136 | Neurotransmitter biosynthetic process | 1 | 0.014222 |
| GO:0046885 | Regulation of hormone biosynthetic process | 1 | 0.014222 |
| GO:2000831 | Regulation of steroid hormone secretion | 1 | 0.014222 |
| GO:0030595 | Leukocyte chemotaxis | 2 | 0.014228 |
| GO:0009743 | Response to carbohydrate | 2 | 0.014345 |
| GO:1903039 | Positive regulation of leukocyte cell-cell adhesion | 2 | 0.014579 |
| GO:0001991 | Regulation of systemic arterial blood pressure by circulatory renin-angiotensin | 1 | 0.015006 |
| GO:0006704 | Glucocorticoid biosynthetic process | 1 | 0.015006 |
| GO:0150079 | Negative regulation of neuroinflammatory response | 1 | 0.015006 |
| GO:1905288 | Vascular associated smooth muscle cell apoptotic process | 1 | 0.015006 |
| GO:1905459 | Regulation of vascular associated smooth muscle cell apoptotic process | 1 | 0.015006 |
| GO:0070663 | Regulation of leukocyte proliferation | 2 | 0.015173 |
| GO:0001832 | Blastocyst growth | 1 | 0.01579 |
| GO:0002544 | Chronic inflammatory response | 1 | 0.01579 |
| GO:0006144 | Purine nucleobase metabolic process | 1 | 0.01579 |
| GO:0060039 | Pericardium development | 1 | 0.01579 |
| GO:1902644 | Tertiary alcohol metabolic process | 1 | 0.01579 |
| GO:0009713 | Catechol-containing compound biosynthetic process | 1 | 0.016573 |
| GO:0035929 | Steroid hormone secretion | 1 | 0.016573 |
| GO:0042423 | Catecholamine biosynthetic process | 1 | 0.016573 |
| GO:0043651 | Linoleic acid metabolic process | 1 | 0.016573 |
| GO:0060008 | Sertoli cell differentiation | 1 | 0.016573 |
| GO:0072111 | Cell proliferation involved in kidney development | 1 | 0.016573 |
| GO:0090201 | Negative regulation of release of cytochrome c from mitochondria | 1 | 0.016573 |
| GO:0090257 | Regulation of muscle system process | 2 | 0.017142 |
| GO:0006071 | Glycerol metabolic process | 1 | 0.017356 |
| GO:0010560 | Positive regulation of glycoprotein biosynthetic process | 1 | 0.017356 |
| GO:0071467 | Cellular response to pH | 1 | 0.017356 |
| GO:0003007 | Heart morphogenesis | 2 | 0.017396 |
| GO:0050730 | Regulation of peptidyl-tyrosine phosphorylation | 2 | 0.018036 |
| GO:0006063 | Uronic acid metabolic process | 1 | 0.018138 |
| GO:0016338 | Calcium-independent cell-cell adhesion via plasma membrane cell-adhesion molecules | 1 | 0.018138 |
| GO:0019585 | Glucuronate metabolic process | 1 | 0.018138 |
| GO:1901522 | Positive regulation of transcription from RNA polymerase II promoter involved in cellular response to chemical stimulus | 1 | 0.018138 |
| GO:2000726 | Negative regulation of cardiac muscle cell differentiation | 1 | 0.018138 |
| GO:0043406 | Positive regulation of MAP kinase activity | 2 | 0.018166 |
| GO:0001759 | Organ induction | 1 | 0.01892 |
| GO:0003181 | Atrioventricular valve morphogenesis | 1 | 0.01892 |
| GO:0021871 | Forebrain regionalization | 1 | 0.01892 |
| GO:0045821 | Positive regulation of glycolytic process | 1 | 0.01892 |
| GO:0045927 | Positive regulation of growth | 2 | 0.019481 |
| GO:0003071 | Renal system process involved in regulation of systemic arterial blood pressure | 1 | 0.019701 |
| GO:0010447 | Response to acidic pH | 1 | 0.019701 |
| GO:0019400 | Alditol metabolic process | 1 | 0.019701 |
| GO:0046697 | Decidualization | 1 | 0.019701 |
| GO:0061050 | Regulation of cell growth involved in cardiac muscle cell development | 1 | 0.019701 |
| GO:2000679 | Positive regulation of transcription regulatory region DNA binding | 1 | 0.019701 |
| GO:0022409 | Positive regulation of cell-cell adhesion | 2 | 0.020154 |
| GO:0007548 | Sex differentiation | 2 | 0.02029 |
| GO:0003081 | Regulation of systemic arterial blood pressure by renin-angiotensin | 1 | 0.020481 |
| GO:0003171 | Atrioventricular valve development | 1 | 0.020481 |
| GO:0006700 | C21-steroid hormone biosynthetic process | 1 | 0.020481 |
| GO:0008211 | Glucocorticoid metabolic process | 1 | 0.020481 |
| GO:0035902 | Response to immobilization stress | 1 | 0.020481 |
| GO:0042104 | Positive regulation of activated T cell proliferation | 1 | 0.020481 |
| GO:0043501 | Skeletal muscle adaptation | 1 | 0.020481 |
| GO:0071377 | Cellular response to glucagon stimulus | 1 | 0.020481 |
| GO:1903020 | Positive regulation of glycoprotein metabolic process | 1 | 0.020481 |
| GO:0001822 | Kidney development | 2 | 0.020699 |
| GO:0046651 | Lymphocyte proliferation | 2 | 0.020699 |
| GO:0032943 | Mononuclear cell proliferation | 2 | 0.021112 |
| GO:0034698 | Response to gonadotropin | 1 | 0.021261 |
| GO:0042481 | Regulation of odontogenesis | 1 | 0.021261 |
| GO:1905208 | Negative regulation of cardiocyte differentiation | 1 | 0.021261 |
| GO:0072001 | Renal system development | 2 | 0.021949 |
| GO:0009074 | Aromatic amino acid family catabolic process | 1 | 0.022041 |
| GO:0010894 | Negative regulation of steroid biosynthetic process | 1 | 0.022041 |
| GO:0043901 | Negative regulation of multi-organism process | 1 | 0.022041 |
| GO:0051450 | Myoblast proliferation | 1 | 0.022041 |
| GO:0044262 | Cellular carbohydrate metabolic process | 2 | 0.022231 |
| GO:0051146 | Striated muscle cell differentiation | 2 | 0.022372 |
| GO:0031647 | Regulation of protein stability | 2 | 0.022514 |
| GO:2000727 | Positive regulation of cardiac muscle cell differentiation | 1 | 0.022819 |
| GO:0005979 | Regulation of glycogen biosynthetic process | 1 | 0.023597 |
| GO:0010962 | Regulation of glucan biosynthetic process | 1 | 0.023597 |
| GO:0033688 | Regulation of osteoblast proliferation | 1 | 0.023597 |
| GO:0042133 | Neurotransmitter metabolic process | 1 | 0.023597 |
| GO:0045939 | Negative regulation of steroid metabolic process | 1 | 0.023597 |
| GO:0042063 | Gliogenesis | 2 | 0.0241 |
| GO:0034368 | Protein-lipid complex remodeling | 1 | 0.024375 |
| GO:0034369 | Plasma lipoprotein particle remodeling | 1 | 0.024375 |
| GO:0048710 | Regulation of astrocyte differentiation | 1 | 0.024375 |
| GO:0060914 | Heart formation | 1 | 0.024375 |
| GO:0071549 | Cellular response to dexamethasone stimulus | 1 | 0.024375 |
| GO:1900745 | Positive regulation of p38MAPK cascade | 1 | 0.024375 |
| GO:0060326 | Cell chemotaxis | 2 | 0.024688 |
| GO:0070661 | Leukocyte proliferation | 2 | 0.024984 |
| GO:0003016 | Respiratory system process | 1 | 0.025152 |
| GO:0032350 | Regulation of hormone metabolic process | 1 | 0.025152 |
| GO:0034367 | Protein-containing complex remodeling | 1 | 0.025152 |
| GO:0060317 | Cardiac epithelial to mesenchymal transition | 1 | 0.025152 |
| GO:0061036 | Positive regulation of cartilage development | 1 | 0.025152 |
| GO:0007568 | Aging | 2 | 0.025882 |
| GO:0002691 | Regulation of cellular extravasation | 1 | 0.025928 |
| GO:0061311 | Cell surface receptor signaling pathway involved in heart development | 1 | 0.025928 |
| GO:0090022 | Regulation of neutrophil chemotaxis | 1 | 0.025928 |
| GO:0120255 | Olefinic compound biosynthetic process | 1 | 0.025928 |
| GO:1902003 | Regulation of amyloid-beta formation | 1 | 0.025928 |
| GO:0009914 | Hormone transport | 2 | 0.026488 |
| GO:0051235 | Maintenance of location | 2 | 0.02664 |
| GO:0010922 | Positive regulation of phosphatase activity | 1 | 0.026704 |
| GO:0032148 | Activation of protein kinase B activity | 1 | 0.026704 |
| GO:0070371 | ERK1 and ERK2 cascade | 2 | 0.026793 |
| GO:1903037 | Regulation of leukocyte cell-cell adhesion | 2 | 0.027407 |
| GO:0003203 | Endocardial cushion morphogenesis | 1 | 0.027479 |
| GO:0006739 | NADP metabolic process | 1 | 0.027479 |
| GO:0009112 | Nucleobase metabolic process | 1 | 0.027479 |
| GO:0010259 | Multicellular organism aging | 1 | 0.027479 |
| GO:0014904 | Myotube cell development | 1 | 0.027479 |
| GO:0016486 | Peptide hormone processing | 1 | 0.027479 |
| GO:0033687 | Osteoblast proliferation | 1 | 0.027479 |
| GO:0034390 | Smooth muscle cell apoptotic process | 1 | 0.027479 |
| GO:0034391 | Regulation of smooth muscle cell apoptotic process | 1 | 0.027479 |
| GO:0070884 | Regulation of calcineurin-NFAT signaling cascade | 1 | 0.027479 |
| GO:0140448 | Signaling receptor ligand precursor processing | 1 | 0.027479 |
| GO:0001655 | Urogenital system development | 2 | 0.027562 |
| GO:0032147 | Activation of protein kinase activity | 2 | 0.027717 |
| GO:0050863 | Regulation of T cell activation | 2 | 0.027872 |
| GO:0001893 | Maternal placenta development | 1 | 0.028254 |
| GO:0010092 | Specification of animal organ identity | 1 | 0.028254 |
| GO:0031128 | Developmental induction | 1 | 0.028254 |
| GO:0033762 | Response to glucagon | 1 | 0.028254 |
| GO:0070873 | Regulation of glycogen metabolic process | 1 | 0.028254 |
| GO:0106056 | Regulation of calcineurin-mediated signaling | 1 | 0.028254 |
| GO:0006882 | Cellular zinc ion homeostasis | 1 | 0.029028 |
| GO:0090322 | Regulation of superoxide metabolic process | 1 | 0.029028 |
| GO:0043405 | Regulation of MAP kinase activity | 2 | 0.029444 |
| GO:0003156 | Regulation of animal organ formation | 1 | 0.029802 |
| GO:0003298 | Physiological muscle hypertrophy | 1 | 0.029802 |
| GO:0003301 | Physiological cardiac muscle hypertrophy | 1 | 0.029802 |
| GO:0032885 | Regulation of polysaccharide biosynthetic process | 1 | 0.029802 |
| GO:0061049 | Cell growth involved in cardiac muscle cell development | 1 | 0.029802 |
| GO:0071902 | Positive regulation of protein serine/threonine kinase activity | 2 | 0.029923 |
| GO:0048545 | Response to steroid hormone | 2 | 0.030083 |
| GO:0030049 | Muscle filament sliding | 1 | 0.030575 |
| GO:0032691 | Negative regulation of interleukin-1 beta production | 1 | 0.030575 |
| GO:0033275 | Actin-myosin filament sliding | 1 | 0.030575 |
| GO:0034205 | Amyloid-beta formation | 1 | 0.030575 |
| GO:0046326 | Positive regulation of glucose import | 1 | 0.030575 |
| GO:0055069 | Zinc ion homeostasis | 1 | 0.030575 |
| GO:0001990 | Regulation of systemic arterial blood pressure by hormone | 1 | 0.031347 |
| GO:0008207 | C21-steroid hormone metabolic process | 1 | 0.031347 |
| GO:0009072 | Aromatic amino acid family metabolic process | 1 | 0.031347 |
| GO:0010613 | Positive regulation of cardiac muscle hypertrophy | 1 | 0.031347 |
| GO:0030501 | Positive regulation of bone mineralization | 1 | 0.031347 |
| GO:0043403 | Skeletal muscle tissue regeneration | 1 | 0.031347 |
| GO:0140353 | Lipid export from cell | 1 | 0.031347 |
| GO:1902895 | Positive regulation of pri-miRNA transcription by RNA polymerase II | 1 | 0.031347 |
| GO:1902991 | Regulation of amyloid precursor protein catabolic process | 1 | 0.031347 |
| GO:0014742 | Positive regulation of muscle hypertrophy | 1 | 0.032119 |
| GO:0030890 | Positive regulation of B cell proliferation | 1 | 0.032119 |
| GO:0050832 | Defense response to fungus | 1 | 0.032119 |
| GO:0071548 | Response to dexamethasone | 1 | 0.032119 |
| GO:1905209 | Positive regulation of cardiocyte differentiation | 1 | 0.032119 |
| GO:2000826 | Regulation of heart morphogenesis | 1 | 0.032119 |
| GO:0001666 | Response to hypoxia | 2 | 0.032198 |
| GO:0009268 | Response to pH | 1 | 0.03289 |
| GO:0010907 | Positive regulation of glucose metabolic process | 1 | 0.03289 |
| GO:0021983 | Pituitary gland development | 1 | 0.03289 |
| GO:0046006 | Regulation of activated T cell proliferation | 1 | 0.03289 |
| GO:1902622 | Regulation of neutrophil migration | 1 | 0.03289 |
| GO:0046394 | Carboxylic acid biosynthetic process | 2 | 0.033528 |
| GO:0051098 | Regulation of binding | 2 | 0.033528 |
| GO:0003009 | Skeletal muscle contraction | 1 | 0.033661 |
| GO:0019432 | Triglyceride biosynthetic process | 1 | 0.033661 |
| GO:0040019 | Positive regulation of embryonic development | 1 | 0.033661 |
| GO:0050850 | Positive regulation of calcium-mediated signaling | 1 | 0.033661 |
| GO:0051154 | Negative regulation of striated muscle cell differentiation | 1 | 0.033661 |
| GO:0150077 | Regulation of neuroinflammatory response | 1 | 0.033661 |
| GO:0016053 | Organic acid biosynthetic process | 2 | 0.033696 |
| GO:0006790 | Sulfur compound metabolic process | 2 | 0.034202 |
| GO:0036293 | Response to decreased oxygen levels | 2 | 0.034202 |
| GO:0010463 | Mesenchymal cell proliferation | 1 | 0.034431 |
| GO:0032881 | Regulation of polysaccharide metabolic process | 1 | 0.034431 |
| GO:0033173 | Calcineurin-NFAT signaling cascade | 1 | 0.034431 |
| GO:0042181 | Ketone biosynthetic process | 1 | 0.034431 |
| GO:0044060 | Regulation of endocrine process | 1 | 0.034431 |
| GO:0045687 | Positive regulation of glial cell differentiation | 1 | 0.034431 |
| GO:0018108 | Peptidyl-tyrosine phosphorylation | 2 | 0.03471 |
| GO:0003197 | Endocardial cushion development | 1 | 0.0352 |
| GO:0010828 | Positive regulation of glucose transmembrane transport | 1 | 0.0352 |
| GO:0014002 | Astrocyte development | 1 | 0.0352 |
| GO:0045616 | Regulation of keratinocyte differentiation | 1 | 0.0352 |
| GO:0046189 | Phenol-containing compound biosynthetic process | 1 | 0.0352 |
| GO:0055026 | Negative regulation of cardiac muscle tissue development | 1 | 0.0352 |
| GO:1904646 | Cellular response to amyloid-beta | 1 | 0.0352 |
| GO:0006959 | Humoral immune response | 2 | 0.035222 |
| GO:0018212 | Peptidyl-tyrosine modification | 2 | 0.035222 |
| GO:0045666 | Positive regulation of neuron differentiation | 2 | 0.035736 |
| GO:0005978 | Glycogen biosynthetic process | 1 | 0.035969 |
| GO:0009250 | Glucan biosynthetic process | 1 | 0.035969 |
| GO:0032692 | Negative regulation of interleukin-1 production | 1 | 0.035969 |
| GO:0050798 | Activated T cell proliferation | 1 | 0.035969 |
| GO:0051646 | Mitochondrion localization | 1 | 0.035969 |
| GO:1900744 | Regulation of p38MAPK cascade | 1 | 0.035969 |
| GO:0001701 | In utero embryonic development | 2 | 0.036254 |
| GO:0010559 | Regulation of glycoprotein biosynthetic process | 1 | 0.036738 |
| GO:0042572 | Retinol metabolic process | 1 | 0.036738 |
| GO:0048599 | Oocyte development | 1 | 0.036738 |
| GO:0060986 | Endocrine hormone secretion | 1 | 0.036738 |
| GO:1903115 | Regulation of actin filament-based movement | 1 | 0.036738 |
| GO:0042692 | Muscle cell differentiation | 2 | 0.037473 |
| GO:0010862 | Positive regulation of pathway-restricted SMAD protein phosphorylation | 1 | 0.037505 |
| GO:0014911 | Positive regulation of smooth muscle cell migration | 1 | 0.037505 |
| GO:0055010 | Ventricular cardiac muscle tissue morphogenesis | 1 | 0.037505 |
| GO:0055023 | Positive regulation of cardiac muscle tissue growth | 1 | 0.037505 |
| GO:0070169 | Positive regulation of biomineral tissue development | 1 | 0.037505 |
| GO:0110151 | Positive regulation of biomineralization | 1 | 0.037505 |
| GO:0002067 | Glandular epithelial cell differentiation | 1 | 0.038273 |
| GO:0006692 | Prostanoid metabolic process | 1 | 0.038273 |
| GO:0006693 | Prostaglandin metabolic process | 1 | 0.038273 |
| GO:0018198 | Peptidyl-cysteine modification | 1 | 0.038273 |
| GO:0042398 | Cellular modified amino acid biosynthetic process | 1 | 0.038273 |
| GO:0090199 | Regulation of release of cytochrome c from mitochondria | 1 | 0.038273 |
| GO:0097720 | Calcineurin-mediated signaling | 1 | 0.038273 |
| GO:0120178 | Steroid hormone biosynthetic process | 1 | 0.038273 |
| GO:0030198 | Extracellular matrix organization | 2 | 0.038354 |
| GO:0050678 | Regulation of epithelial cell proliferation | 2 | 0.038354 |
| GO:0006631 | Fatty acid metabolic process | 2 | 0.038531 |
| GO:0043062 | Extracellular structure organization | 2 | 0.038531 |
| GO:0070482 | Response to oxygen levels | 2 | 0.038531 |
| GO:1901653 | Cellular response to peptide | 2 | 0.038886 |
| GO:0003044 | Regulation of systemic arterial blood pressure mediated by a chemical signal | 1 | 0.039039 |
| GO:0010718 | Positive regulation of epithelial to mesenchymal transition | 1 | 0.039039 |
| GO:0019674 | NAD metabolic process | 1 | 0.039039 |
| GO:0031648 | Protein destabilization | 1 | 0.039039 |
| GO:0042987 | Amyloid precursor protein catabolic process | 1 | 0.039039 |
| GO:0045981 | Positive regulation of nucleotide metabolic process | 1 | 0.039039 |
| GO:1900544 | Positive regulation of purine nucleotide metabolic process | 1 | 0.039039 |
| GO:1903580 | Positive regulation of ATP metabolic process | 1 | 0.039039 |
| GO:1904707 | Positive regulation of vascular associated smooth muscle cell proliferation | 1 | 0.039039 |
| GO:0046460 | Neutral lipid biosynthetic process | 1 | 0.039805 |
| GO:0046463 | Acylglycerol biosynthetic process | 1 | 0.039805 |
| GO:0048146 | Positive regulation of fibroblast proliferation | 1 | 0.039805 |
| GO:0071827 | Plasma lipoprotein particle organization | 1 | 0.039805 |
| GO:1902893 | Regulation of pri-miRNA transcription by RNA polymerase II | 1 | 0.039805 |
| GO:0032970 | Regulation of actin filament-based process | 2 | 0.04014 |
| GO:0003179 | Heart valve morphogenesis | 1 | 0.040571 |
| GO:0009994 | Oocyte differentiation | 1 | 0.040571 |
| GO:0038066 | P38MAPK cascade | 1 | 0.040571 |
| GO:0060421 | Positive regulation of heart growth | 1 | 0.040571 |
| GO:0071622 | Regulation of granulocyte chemotaxis | 1 | 0.040571 |
| GO:0072132 | Mesenchyme morphogenesis | 1 | 0.040571 |
| GO:2000677 | Regulation of transcription regulatory region DNA binding | 1 | 0.040571 |
| GO:0061614 | Pri-miRNA transcription by RNA polymerase II | 1 | 0.041336 |
| GO:0071320 | Cellular response to cAMP | 1 | 0.041336 |
| GO:0001503 | Ossification | 2 | 0.041409 |
| GO:2001233 | Regulation of apoptotic signaling pathway | 2 | 0.041591 |
| GO:0009100 | Glycoprotein metabolic process | 2 | 0.041957 |
| GO:0006584 | Catecholamine metabolic process | 1 | 0.0421 |
| GO:0009620 | Response to fungus | 1 | 0.0421 |
| GO:0009712 | Catechol-containing compound metabolic process | 1 | 0.0421 |
| GO:1903018 | Regulation of glycoprotein metabolic process | 1 | 0.0421 |
| GO:0003229 | Ventricular cardiac muscle tissue development | 1 | 0.042864 |
| GO:0014888 | Striated muscle adaptation | 1 | 0.042864 |
| GO:0032720 | Negative regulation of tumor necrosis factor production | 1 | 0.042864 |
| GO:0050879 | Multicellular organismal movement | 1 | 0.042864 |
| GO:0050881 | Musculoskeletal movement | 1 | 0.042864 |
| GO:0070741 | Response to interleukin-6 | 1 | 0.042864 |
| GO:0071825 | Protein-lipid complex subunit organization | 1 | 0.042864 |
| GO:0001558 | Regulation of cell growth | 2 | 0.042878 |
| GO:0060563 | Neuroepithelial cell differentiation | 1 | 0.043627 |
| GO:1904645 | Response to amyloid-beta | 1 | 0.043627 |
| GO:0045785 | Positive regulation of cell adhesion | 2 | 0.044367 |
| GO:0045840 | Positive regulation of mitotic nuclear division | 1 | 0.04439 |
| GO:0048016 | Inositol phosphate-mediated signaling | 1 | 0.04439 |
| GO:1903556 | Negative regulation of tumor necrosis factor superfamily cytokine production | 1 | 0.04439 |
| GO:0019216 | Regulation of lipid metabolic process | 2 | 0.04493 |
| GO:0010656 | Negative regulation of muscle cell apoptotic process | 1 | 0.045152 |
| GO:0035306 | Positive regulation of dephosphorylation | 1 | 0.045152 |
| GO:0051055 | Negative regulation of lipid biosynthetic process | 1 | 0.045152 |
| GO:0001658 | Branching involved in ureteric bud morphogenesis | 1 | 0.045913 |
| GO:0071385 | Cellular response to glucocorticoid stimulus | 1 | 0.045913 |
| GO:0001836 | Release of cytochrome c from mitochondria | 1 | 0.046674 |
| GO:0010823 | Negative regulation of mitochondrion organization | 1 | 0.046674 |
| GO:0043388 | Positive regulation of DNA binding | 1 | 0.046674 |
| GO:2000242 | Negative regulation of reproductive process | 1 | 0.046674 |
| GO:0048608 | Reproductive structure development | 2 | 0.047211 |
| GO:0048732 | Gland development | 2 | 0.047211 |
| GO:0003170 | Heart valve development | 1 | 0.047435 |
| GO:0045843 | Negative regulation of striated muscle tissue development | 1 | 0.047435 |
| GO:0046324 | Regulation of glucose import | 1 | 0.047435 |
| GO:0061458 | Reproductive system development | 2 | 0.047981 |
| GO:0010676 | Positive regulation of cellular carbohydrate metabolic process | 1 | 0.048194 |
| GO:0030888 | Regulation of B cell proliferation | 1 | 0.048194 |
| GO:0048635 | Negative regulation of muscle organ development | 1 | 0.048194 |
| GO:0055025 | Positive regulation of cardiac muscle tissue development | 1 | 0.048194 |
| GO:0060393 | Regulation of pathway-restricted SMAD protein phosphorylation | 1 | 0.048194 |
| GO:0071384 | Cellular response to corticosteroid stimulus | 1 | 0.048194 |
| GO:2001244 | Positive regulation of intrinsic apoptotic signaling pathway | 1 | 0.048194 |
| GO:0006081 | Cellular aldehyde metabolic process | 1 | 0.048954 |
| GO:0046622 | Positive regulation of organ growth | 1 | 0.048954 |
| GO:0050673 | Epithelial cell proliferation | 2 | 0.049145 |
| GO:0030166 | Proteoglycan biosynthetic process | 1 | 0.049712 |
| GO:0046173 | Polyol biosynthetic process | 1 | 0.049712 |
| GO:0060135 | Maternal process involved in female pregnancy | 1 | 0.049712 |
| GO:1901862 | Negative regulation of muscle tissue development | 1 | 0.049712 |
| GO:0019932 | Second-messenger-mediated signaling | 2 | 0.049731 |
| GO:0062012 | Regulation of small molecule metabolic process | 2 | 0.049731 |

**Supplementary Table 4. The items of CC in GO enrichment of the targets of YSHS granule against FSGS.**

| **ID** | **Description** | **Count** | **P Value** |
| --- | --- | --- | --- |
| GO:0034774 | secretory granule lumen | 4 | 8.53E-05 |
| GO:0060205 | cytoplasmic vesicle lumen | 4 | 8.94E-05 |
| GO:0031983 | vesicle lumen | 4 | 9.16E-05 |
| GO:0031093 | platelet alpha granule lumen | 2 | 0.001179 |
| GO:0031091 | platelet alpha granule | 2 | 0.002161 |
| GO:0005902 | microvillus | 2 | 0.002256 |
| GO:0005775 | vacuolar lumen | 2 | 0.007572 |
| GO:0001931 | uropod | 1 | 0.009927 |
| GO:0031254 | cell trailing edge | 1 | 0.009927 |
| GO:0098858 | actin-based cell projection | 2 | 0.012009 |
| GO:0005865 | striated muscle thin filament | 1 | 0.016746 |
| GO:0036379 | myofilament | 1 | 0.019762 |
| GO:0045177 | apical part of cell | 2 | 0.042448 |

**Supplementary Table 5. The items of MF in GO enrichment of the targets of YSHS granule against FSGS.**

| **ID** | **Description** | **Count** | **P Value** |
| --- | --- | --- | --- |
| GO:0005504 | fatty acid binding | 3 | 3.97E-06 |
| GO:0031406 | carboxylic acid binding | 4 | 2.14E-05 |
| GO:0033293 | monocarboxylic acid binding | 3 | 2.55E-05 |
| GO:0043177 | organic acid binding | 4 | 2.65E-05 |
| GO:0016209 | antioxidant activity | 3 | 4.34E-05 |
| GO:0005159 | insulin-like growth factor receptor binding | 2 | 7.43E-05 |
| GO:0004364 | glutathione transferase activity | 2 | 0.000185 |
| GO:0016765 | transferase activity, transferring alkyl or aryl (other than methyl) groups | 2 | 0.000936 |
| GO:0048306 | calcium-dependent protein binding | 2 | 0.002141 |
| GO:0016616 | oxidoreductase activity, acting on the CH-OH group of donors, NAD or NADP as acceptor | 2 | 0.004211 |
| GO:0005179 | hormone activity | 2 | 0.004349 |
| GO:0016614 | oxidoreductase activity, acting on CH-OH group of donors | 2 | 0.004922 |
| GO:0048018 | receptor ligand activity | 3 | 0.006661 |
| GO:0030546 | signaling receptor activator activity | 3 | 0.006852 |
| GO:0008083 | growth factor activity | 2 | 0.007541 |
| GO:0005527 | macrolide binding | 1 | 0.008957 |
| GO:0015643 | toxic substance binding | 1 | 0.008957 |
| GO:0050786 | RAGE receptor binding | 1 | 0.008957 |
| GO:0035325 | Toll-like receptor binding | 1 | 0.009767 |
| GO:0016829 | lyase activity | 2 | 0.010143 |
| GO:0039706 | co-receptor binding | 1 | 0.010577 |
| GO:0070700 | BMP receptor binding | 1 | 0.010577 |
| GO:0016863 | intramolecular oxidoreductase activity, transposing C=C bonds | 1 | 0.011386 |
| GO:0036041 | long-chain fatty acid binding | 1 | 0.012195 |
| GO:0003953 | NAD+ nucleosidase activity | 1 | 0.013003 |
| GO:0019211 | phosphatase activator activity | 1 | 0.013003 |
| GO:0050135 | NAD(P)+ nucleosidase activity | 1 | 0.013003 |
| GO:0061809 | NAD+ nucleotidase, cyclic ADP-ribose generating | 1 | 0.013003 |
| GO:0004745 | retinol dehydrogenase activity | 1 | 0.016229 |
| GO:0004602 | glutathione peroxidase activity | 1 | 0.017034 |
| GO:0009975 | cyclase activity | 1 | 0.017838 |
| GO:0016849 | phosphorus-oxygen lyase activity | 1 | 0.017838 |
| GO:0005158 | insulin receptor binding | 1 | 0.018642 |
| GO:0016769 | transferase activity, transferring nitrogenous groups | 1 | 0.018642 |
| GO:0070696 | transmembrane receptor protein serine/threonine kinase binding | 1 | 0.019445 |
| GO:0004190 | aspartic-type endopeptidase activity | 1 | 0.020248 |
| GO:0070001 | aspartic-type peptidase activity | 1 | 0.02105 |
| GO:0033612 | receptor serine/threonine kinase binding | 1 | 0.023452 |
| GO:1901567 | fatty acid derivative binding | 1 | 0.023452 |
| GO:0016799 | hydrolase activity, hydrolyzing N-glycosyl compounds | 1 | 0.027443 |
| GO:0016831 | carboxy-lyase activity | 1 | 0.028239 |
| GO:0019825 | oxygen binding | 1 | 0.029035 |
| GO:0004177 | aminopeptidase activity | 1 | 0.036959 |
| GO:0016860 | intramolecular oxidoreductase activity | 1 | 0.040112 |
| GO:0016830 | carbon-carbon lyase activity | 1 | 0.040899 |
| GO:0004601 | peroxidase activity | 1 | 0.041685 |
| GO:0030170 | pyridoxal phosphate binding | 1 | 0.04404 |
| GO:0070279 | vitamin B6 binding | 1 | 0.04404 |
| GO:0016684 | oxidoreductase activity, acting on peroxide as acceptor | 1 | 0.044823 |
| GO:0005507 | copper ion binding | 1 | 0.047952 |
| GO:0016655 | oxidoreductase activity, acting on NAD(P)H, quinone or similar compound as acceptor | 1 | 0.048733 |
| GO:0004175 | endopeptidase activity | 2 | 0.049012 |

**Supplementary Table 6. KEGG pathway enrichment of the targets of YSHS granule against FSGS.**

| **ID** | **Description** | **Count** | **P Value** |
| --- | --- | --- | --- |
| hsa00480 | Glutathione metabolism | 2 | 0.004832 |
| hsa00982 | Drug metabolism - cytochrome P450 | 2 | 0.007616 |
| hsa01524 | Platinum drug resistance | 2 | 0.007822 |
| hsa04918 | Thyroid hormone synthesis | 2 | 0.008242 |
| hsa00980 | Metabolism of xenobiotics by cytochrome P450 | 2 | 0.00889 |
| hsa00983 | Drug metabolism - other enzymes | 2 | 0.009335 |
| hsa05204 | Chemical carcinogenesis | 2 | 0.010021 |
| hsa05410 | Hypertrophic cardiomyopathy | 2 | 0.011705 |
| hsa05414 | Dilated cardiomyopathy | 2 | 0.013242 |
| hsa04152 | AMPK signaling pathway | 2 | 0.02021 |
| hsa04068 | FoxO signaling pathway | 2 | 0.023821 |
| hsa05418 | Fluid shear stress and atherosclerosis | 2 | 0.026604 |
| hsa00360 | Phenylalanine metabolism | 1 | 0.031099 |
| hsa05225 | Hepatocellular carcinoma | 2 | 0.037728 |
| hsa04614 | Renin-angiotensin system | 1 | 0.041858 |
| hsa04964 | Proximal tubule bicarbonate reclamation | 1 | 0.041858 |
| hsa00790 | Folate biosynthesis | 1 | 0.047195 |
